# Enhanced Early Detection of Colorectal Cancer via Blood Biomarker Combinations Identified Through Extracellular Vesicle Isolation and Artificial Intelligence Analysis

**DOI:** 10.1002/jev2.70088

**Published:** 2025-06-13

**Authors:** Bonhan Koo, Young Il Kim, Minju Lee, Seok‐Byung Lim, Yong Shin

**Affiliations:** ^1^ Department of Biotechnology, College of Life Science and Biotechnology Yonsei University Seodaemun‐gu Republic of Korea; ^2^ Division of Colon and Rectal Surgery, Department of Surgery, Asan Medical Center University of Ulsan College of Medicine Songpa‐gu Republic of Korea

**Keywords:** artificial intelligence analysis, biomarker combinations, blood biomarkers, early diagnostics, extracellular vesicle isolation

## Abstract

Colorectal cancer (CRC) remains a major cause of cancer‐related deaths worldwide, with early detection being crucial for improving survival rates. Despite the potential of extracellular vesicles (EVs) as blood biomarkers for CRC diagnosis, their clinical utility is limited due to complex and time‐consuming isolation methods, unverified biomarkers and low diagnostic performance. Here, we introduce the ZAHV‐AI system, which combines the zeolite‐amine and homobifunctional hydrazide‐based extracellular vesicle isolation (ZAHVIS) platform with AI‐driven analysis for enhanced CRC diagnosis. The ZAHVIS platform enables simple, rapid and cost‐effective EV isolation and one‐step extraction of EV‐derived proteins and nucleic acids (NAs), providing a streamlined approach. Using blood plasma samples from 80 CRC patients across all stages and 20 healthy individuals, we identified four EV‐derived miRNA blood biomarkers (miR‐23a‐3p, miR‐92a‐3p, miR‐125a‐3p and miR‐150‐5p) by confirming statistical significance with relative quantification (RQ) values from real‐time PCR and integrated these with carcinoembryonic antigen (CEA) levels into an AI‐driven diagnostic model. Among 31 combinations used to identify optimal sets, optimal combination (miR‐23a‐3p, miR‐92a‐3p, miR‐150‐5p and CEA) for overall CRC achieved an area under the curve (AUC) of 0.9861, outperforming individual markers and conventional CEA tests. Notably, the system achieved perfect performance in detecting stages 0–1 (AUC: 1.0) and demonstrated high accuracy for stage 2 (AUC: 0.9722) and early‐stage CRC (AUC: 0.9861), using stage‐specific optimal combinations. Therefore, the ZAHV‐AI system offers a reliable and clinically relevant tool for CRC diagnostics, significantly enhancing early detection and monitoring capabilities.

## Graphical abstract

The ZAHV‐AI system integrates the ZAHVIS platform for efficient EV isolation with AI‐driven analysis, enhancing CRC diagnostics. The ZAHVIS platform provides rapid, cost‐effective EV isolation and one‐step extraction of EV‐derived molecules. AI‐driven analysis optimizes blood biomarker combinations, significantly improving diagnostic accuracy, particularly in early‐stage CRC. This integrated approach offers a robust, streamlined solution for reliable CRC detection in clinical practice.

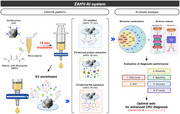

## 1 Introduction

Colorectal cancer (CRC) is a major global health concern, ranking third in new cases and second in cancer‐related deaths (Bray et al. 2024; Dekker et al. 2019; Siegel et al. 2023). Its high fatality rate is largely due to the risks of metastasis and recurrence (Ganesh et al. 2019; Van Cutsem et al. 2016). The progression from normal colonic epithelium to cancer can take 10–20 years, making early‐stage detection challenging (Rawla et al. 2019). However, early detection is crucial for timely treatment and can greatly reduce CRC mortality rates. For example, the 5‐year relative survival rate is approximately 90% for stages 1 and 2, compared to less than 20% for stages 3 and 4 (Biller and Schrag 2021; Petrelli et al. 2017). Colonoscopy with biopsy, the gold standard for CRC diagnosis, offers the advantage of direct visualization of the entire colon and the ability to immediately detect and remove suspicious growths or precancerous polyps (Amri et al. 2013; Bretthauer et al. 2022). However, its invasiveness leads to patient discomfort and anxiety, and it presents challenges related to cost and accessibility. Only 54% of individuals with high‐risk adenomas adhere to the recommended interval for repeated colonoscopy screenings (Murphy et al. 2016). As a less invasive alternative, blood‐based tests using conventional tumour markers, such as carcinoembryonic antigen (CEA), have been explored primarily for screening purposes (Hall et al. 2019). CEA, a glycoprotein in the human digestive system, serves as a crucial prognostic marker for CRC, with elevated levels (> 5.0 ng mL^−1^) typically observed in CRC patients compared to healthy individuals (< 2.5 ng mL^−1^ for non‐smokers and < 5.0 ng mL^−1^ for smokers) (Ruibal Morell 1992; Sajid et al. 2007). Nevertheless, CEA testing can yield false positives due to benign conditions and has limited sensitivity for early‐stage disease detection, underscoring the pressing need for improved diagnostic methods and blood biomarkers to enhance CRC detection.

Recent advances in liquid biopsy have highlighted the potential of extracellular vesicles (EVs) (van Niel et al. 2018; Wei et al. 2020; Yu et al. 2022). The significance of EVs in various cancer has grown substantially, not only for their potential in enhancing diagnostic accuracy but also for their roles in understanding disease progression, metastasis and therapeutic response (Pucci et al. 2019; Wang et al. 2022; Xu et al. 2018). Present in body fluids like plasma, serum, saliva and urine, EVs are enriched with bioactive molecules such as proteins, nucleic acids (NAs) and lipids reflecting the physiological state of their parent cells (Peinado et al. 2011; Witwer et al. 2013; Xu et al. 2016). Among these molecules, non‐coding RNAs have emerged as critical regulators in cancer biology due to their involvement in gene expression and signalling pathways (Grillone et al. 2020; Xie et al. 2019). Specifically, microRNAs (miRNAs) and circular RNAs (circRNAs) have gained significant interest due to their enhanced stability in bodily fluids, particularly when enclosed in EVs, which protect them from enzymatic degradation, as well as their unique expression profiles in cancer (Chen and Yang 2015; Kristensen et al. 2019; Ogata‐Kawata et al. 2014; Palmero et al. 2011; Wang et al. 2019). miRNAs are small non‐coding RNAs that regulate gene expression, while circRNAs are a type of non‐coding RNA with covalently closed loop structures that can act as miRNA sponges, influencing cancer progression. These properties make EV‐derived miRNAs and circRNAs promising biomarkers for CRC diagnosis, offering the potential for more accurate and early detection (Ferracin et al. 2010; Vakhshiteh et al. 2021; Verduci et al. 2021). However, their reliability as CRC biomarkers remains underexplored, necessitating further research.

Effective isolation of EVs is crucial for obtaining reliable information on the detection sensitivity and accuracy of EV‐derived biomarkers. Traditional methods like ultracentrifugation (UC) and the total exosome isolation (TEI) method, while commonly used, present significant complexities and challenges for clinical application. UC effectively isolates EVs with high purity, but it requires expensive equipment, is labour‐intensive, and can damage EVs due to intense centrifugal forces, affecting the integrity of EV‐derived biomarkers (Tang et al. 2017). Additionally, UC is time‐consuming, requiring several hours to complete multiple centrifugation steps. The TEI method, using a proprietary polymer to precipitate EVs from biological fluids and collecting them by low‐speed centrifugation, offers a simpler and faster alternative. However, TEI has drawbacks, including potential co‐precipitation of non‐EV contaminants and polymer interference with downstream applications like proteomic and RNA analyses (Patel et al. 2019). Size exclusion chromatography (SEC) has recently gained attention for its simplicity, speed and clinical applicability, enabling rapid processing of clinical samples through standardized, commercially available protocols (Boing et al. 2014; Lobb et al. 2015). However, SEC requires collecting multiple fractions to isolate EVs, which can be cumbersome without specialized equipment like an auto fraction collector (Sidhom et al. 2020). When fractions are collected manually, the process is time‐consuming, introduces variability and errors, and reduces reproducibility. Additionally, EVs are distributed across large elution volumes (hundreds of microlitres to millilitres), making SEC more suitable for purification than enrichment. This extensive elution volume can decrease detection sensitivity in downstream analyses that require high EV concentrations, often necessitating additional enrichment steps. These inherent limitations highlight the need for more efficient, reliable and clinically feasible EV isolation techniques.

In this study, we developed an advanced system called ZAHV‐AI, which stands for zeolite‐amine and homobifunctional hydrazide‐based extracellular vesicle isolation (ZAHVIS) and artificial intelligence (AI)‐driven analysis. This system integrates EV isolation with deep learning‐based AI analysis to improve CRC diagnostic accuracy. The ZAHVIS platform uses zeolite‐amine (ZA) and malonic acid dihydrazide (MDH) for efficient EV capture via electrostatic and covalent interactions, followed by syringe filters to effectively filter the ZA with captured EVs. This streamlined one‐step platform is designed for speed, efficiency and cost‐effectiveness, enabling EV isolation in 15 min and the extraction of EV‐derived proteins or NAs with EV enrichment in 30–35 min, thereby enhancing the convenience of downstream analyses. The ZAHV‐AI system leverages AI‐driven analysis to enhance CRC diagnosis by utilizing relative quantification (RQ) values of biomarker candidates through real‐time PCR. By employing optimized deep neural networks, the system refines the process by generating, training and testing various combinations of cellular and clinically validated biomarkers. Our study assessed blood‐derived EVs from a cohort of 100 individuals, including 80 CRC patients (20 in each of stages 0–1, 2, 3 and 4) and 20 healthy control (HC) individuals. To identify potential biomarkers, we selected 15 miRNAs (miR‐19a‐3p (Yu et al. 2020), miR‐21‐5p (Du et al. 2014), miR‐23a‐3p (Jahid et al. 2012; Yong et al. 2014), miR‐92a‐3p (Wei et al. 2019), miR‐99b‐5p (Zhao et al. 2019), miR‐122‐5p (Sun et al. 2020), miR‐125a‐3p (Wang et al. 2017), miR‐141‐3p (Meltzer et al. 2019), miR‐150‐5p (Bakhsh et al. 2024; Zhao et al. 2019), miR‐181a‐5p (Zhao et al. 2022), miR‐182‐5p (Liu et al. 2013), miR‐200b‐3p (Zhang et al. 2018), miR‐222‐5p (Li et al. 2021), miR‐223‐3p (Wang et al. 2023), miR‐1246 (Cooks et al. 2018)) and three circRNAs (hsa_circ_0008558, circLONP2 (Han et al. 2020); hsa_circ_0101802, circPNN (Xie et al. 2020); hsa_circ_0087960, circLPAR1 (Zheng et al. 2022)) based on previous research. Utilizing the ZAHVIS platform, we concentrated EVs and extracted EV‐derived RNAs from the cell culture media of HCT116 cancer cells and CCD‐18co normal cells, and from blood plasma. Although seven miRNAs and three circRNAs were significant in cell line models, only four miRNAs (miR‐23a‐3p, miR‐92a‐3p, miR‐125a‐3p and miR‐150‐5p) were relevant in blood plasma samples. Among these, miR‐150‐5p emerged as the highest‐performing marker with an area under the curve (AUC) of 0.87, compared to 0.75 for CEA. Despite these promising values, significant diagnostic limitations remain due to considerable overlap between CRC and HC samples, making it difficult to use individual biomarkers as reliable diagnostic tools. Notably, the ZAHV‐AI system analyses 31 different combinations to identify the optimal blood biomarker set, achieving exceptional diagnostic performance for CRC across various stages. The combination of miR‐23a‐3p, miR‐92a‐3p, miR‐150‐5p and CEA yielded an AUC of 0.9861 for overall CRC. Stage‐specific outcomes demonstrated high diagnostic performance with various optimal combinations, particularly for early‐stage CRC (AUC: 0.9861) and advanced‐stage CRC (AUC: 0.9583). In addition, the system achieved perfect diagnostic performance (AUC: 1.0) in stages 0–1, 3 and 4, and high performance (AUC: 0.9722) in stage 2. These findings underscore the potential of the ZAHV‐AI system to enhance CRC diagnostics by integrating efficient EV isolation with robust AI analysis, providing precise, accessible and reliable tools for early detection and monitoring in the clinical applications.

## 2 Materials and Methods

### 2.1 Materials

The following materials were obtained from specific suppliers. Zeolite (96096), 3‐Aminopropyl(diethoxy)methylsilane (APDMS, 371890), glutaraldehyde (340855), sodium bicarbonate (S5761) and Apolipoprotein B (ApoB) from human plasma (A5353) were procured from Sigma–Aldrich (St. Louis, MO, USA). Malonic acid dihydrazide (MDH, M3206) was sourced from Tokyo Chemical Industry (Tokyo, Japan). Kovax 1–30 mL and BD 1 mL syringe (309628) were obtained from Korea Vaccine (Ansan‐si, Gyeonggi‐do, South Korea) and Becton, Dickinson and Company (Franklin Lakes, NJ, USA), respectively. Polytetrafluoroethylene (PTFE) 3 µm syringe filter (18140), PTFE 1 µm syringe filter (16278), polyvinylidene fluoride (PVDF) 3 µm syringe filter (18215), PVDF 1 µm syringe filter (18214) were obtained from Tisch Scientific (Cleves, OH, USA). PVDF 0.22 µm syringe filter (FJ25ASCCA002DL01) and PVDF 0.45 µm syringe filter (FJ25ASCCA004FL01) were supplied by GVS Filter Technology (Bologna, Italy). 100X antibiotic‐antimycotic (15240062), exosome‐depleted foetal bovine serum (FBS, A2720803), Dulbecco's modified Eagle medium (DMEM, 41965039) and RPMI 1640 Medium (A1049101) were sourced from Gibco (Waltham, MA, USA). Invitrogen (Waltham, MA, USA) supplied the Total Exosome Isolation (TEI) Reagent (4478359), SuperScript IV First‐Strand Synthesis System (18091050), and Total Exosome RNA & Protein Isolation Kit (4478545). The qEVoriginal column (ICO‐35) for size exclusion chromatography (SEC) was obtained from Izon Science (Christchurch, New Zealand). Pooled Human Plasma (IPLAWBLIH50ML) was sourced from Innovative Research (Novi, MI, USA). The antibodies used were Rabbit Anti‐CD9 antibody (ab236630), Mouse Anti‐CD81 antibody (ab79559), Rabbit Anti‐CD63 antibody (ab134045), Rabbit Anti‐ARF6 antibody (ab131261), Rabbit Anti‐GRP78 antibody (ab108615), Rabbit Anti‐GM130 antibody (ab52649), Rabbit Anti‐ApoB antibody (ab139401), Rabbit Anti‐Apolipoprotein E (ApoE) antibody (ab183597), Goat Anti‐Rabbit IgG/HRP antibody (ab205718), Goat Anti‐Mouse IgG/HRP antibody (ab6789) and Donkey Anti‐Rabbit IgG/Gold antibody (ab39597) from Abcam (Cambridge, UK). Uranyl Acetate Alternative (19485) was purchased from Ted Pella (Redding, CA, USA). Rabbit Anti‐Calnexin antibody (2679S) was obtained from Cell Signalling Technology (Danvers, MA, USA), and Mouse Anti‐Human Serum Albumin (HSA) Antibody (MAB1455) was sourced from R&D Systems (Minneapolis, MN, USA). 10X RIPA lysis buffer (20‐188) was obtained from Merck Millipore (Burlington, MA, USA). RIPA lysis and extraction buffer (89901) and Proteinase K (EO0491) from ThermoFisher Scientific (Waltham, MA, USA), RNase‐free DNase set (79254) from Qiagen (Hilden, Germany), and NP‐40 lysis buffer (J60766) from Alfa Aesar (Haverhill, MA, USA) were used. Mir‐X miRNA qRT‐PCR TB Green Kit (638314) was obtained from Takara Bio (Shiga, Japan) and Brilliant III SYBR Green QPCR Master Mix (600882) was supplied by Agilent Technologies (Santa Clara, CA, USA). The oligonucleotides used were synthesized by BIONICS (Seoul, South Korea) and Macrogen (Seoul, South Korea).

### 2.2 Synthesis of the ZA Particles

The synthesis of ZA particles for use in the ZAHVIS platform was conducted with slight modifications to a previously established protocol (Koo, Kim, Jang, et al. 2023). Initially, 3 g of zeolite were subjected to a dual washing process, involving agitation at 550 rpm for 10 min in deionized water (DW) and 95% ethanol. To ensure uniform particle size, larger zeolite particles that precipitated within 1 min were removed during the first wash. The remaining zeolite suspension was then centrifuged at 1000 rpm for 10 s to eliminate smaller particles, followed by the disposal of the supernatant. The purified zeolite, with an average diameter of approximately 3.5 µm, was subsequently functionalized with amine groups by incubating it in a 2% (v/v) APDMS solution in 95% ethanol under constant stirring at 450 rpm for 4 h. The functionalized ZA particles were washed three times (5 min each at 550 rpm) using DW and 95% ethanol to remove unreacted residues. Finally, the ZA particles were dried in a vacuum chamber for over 24 h to ensure complete solvent removal and stored at room temperature until further use. These ZA particles were then utilized in the ZAHVIS platform for optimization and efficient EV isolation.

### 2.3 Workflow of the ZAHVIS Platform

The ZAHVIS platform is an integrated tool for EV isolation, EV‐derived protein extraction and EV‐derived NA extraction, all with EV enrichment. Initially, 5 mg mL^−1^ of ZA and 25 mg mL^−1^ of MDH were added to biological sample (10 mL of cell culture media or 1 mL of blood plasma) and incubated for 10 min. The mixture was then transferred to a Kovax syringe fitted with a PVDF 0.45 µm syringe filter, and unbound waste was removed by gently pressing the syringe. The EVs bound to ZA and MDH on the filter surface were washed with 3 mL of PBS, followed by air injection to eliminate residual PBS. Following this preparation, EVs could be processed in three ways depending on the intended analysis. For EV isolation, 300 µL of high pH elution buffer (10 mM sodium bicarbonate, pH 10.4) was injected into the filter and incubated for 1 min. The detached EVs were collected by air injection into sterile tubes and either used immediately or stored at 4°C for short‐term use and −80°C for long‐term storage. For protein extraction, 300 µL of RIPA lysis buffer was injected, followed by a 20‐min incubation at 4°C. The extracted proteins were collected by air injection and stored at −20°C or used directly. For RNA extraction, 300 µL of NP‐40 lysis buffer containing 7.5 mg MDH (2.5 mg per 100 µL), 3 µL of proteinase K and 10 µL of RNase‐free DNase I was injected and incubated at room temperature for 20 min. During this step, released RNAs bound to ZA and MDH on the filter surface, while unbound proteins and other molecules were removed by air injection, followed by PBS washing and air drying. Finally, RNA was eluted by injecting 300 µL of high pH elution buffer, incubated for 1 min, and collected by air injection. The isolated RNA was used immediately or stored at −80°C.

### 2.4 Cell Line Models

The HCT116 (ATCC CCL‐247) human colorectal carcinoma cell line and the CCD‐18co (KCLB 21459) normal human colon cell line were acquired from the American Type Culture Collection and the Korean Cell Line Bank, respectively. Both cell lines were cultured under standard conditions at a temperature of 37°C and a CO_2_ concentration of 5%. The DMEM media was supplemented with 10% exosome‐depleted FBS and 1% antibiotic‐antimycotic solution. To isolate EVs from both HCT116 and CCD‐18co cells, the cells were grown until they reached approximately 80% confluence. The cell cultures were then subjected to centrifugation at 400 *g* for 30 min at a temperature of 4°C to obtain the cell‐free supernatant. After filtration using a PVDF 0.22 µm syringe filter, these supernatants were either immediately used for EV isolation or stored at a temperature of −20°C for a maximum of 4 weeks.

### 2.5 EV Isolation Methods

For comparison with the ZAHVIS platform, conventional EV isolation methods including ultracentrifugation (UC), total exosome isolation (TEI) and size‐exclusion chromatography (SEC) were employed. For UC, 10 mL of cell culture media was centrifuged at 300 g for 10 min to remove cell debris, followed by 2000 g for 20 min to eliminate apoptotic bodies. The supernatant was transferred to a new tube and further centrifuged at 10,000 *g* for 30 min to remove larger vesicles and remaining debris. The final supernatant was subjected to ultracentrifugation at 110,000 g for 90 min at 4°C, and the resulting EV pellet was resuspended in 300 µL of PBS. Similarly, for TEI, 10 mL of cell culture media was mixed with 5 mL of TEI reagent and incubated overnight at 4°C. After centrifugation at 10,000 g for 60 min at 4°C, the supernatant was discarded and the EV pellet was resuspended in 300 µL of PBS. For SEC, the chromatography column was equilibrated to room temperature and flushed with two column volumes of PBS. A total of 500 µL of cell culture media was loaded onto the column, and 24 fractions (500 µL each) were sequentially eluted. The EV‐containing fractions (fractions 7–10) were pooled and used directly for downstream analysis. The isolated EV samples were either used immediately or stored at 4°C for short‐term use and −80°C for long‐term storage. For protein analysis, EV pellets obtained from UC and TEI were lysed using 1X RIPA lysis buffer, while SEC‐derived EVs, due to dilution during fractionation, were lysed using 10X RIPA lysis buffer. EV‐derived protein samples were either analysed immediately or stored at −20°C. For EV‐derived NA analysis, RNA was extracted from EV samples obtained using ZAHVIS, UC, TEI and SEC methods, employing the Total Exosome RNA & Protein Isolation Kit according to the manufacturer's protocol. The extracted RNA was either used immediately or stored at −80°C.

### 2.6 EV Characterization

The morphology of the EVs obtained through the ZAHVIS platform, UC, TEI and SEC was evaluated using TEM. A formvar/carbon‐coated copper grid (coated side down) was placed onto each droplet and incubated at room temperature for 5 min. Excess liquid was removed with filter paper, and the grids were transferred to 2.5% glutaraldehyde for 2 min for fixation. After washing twice with DW for 30 s each, the grids were either processed for direct TEM analysis or subjected to immunogold labelling. For CD9 labelling, the grids were blocked with 5% BSA for 20 min, incubated with anti‐CD9 primary antibody (1:25 in PBS) for 30 min at room temperature, and washed three times with PBS. The grids were then incubated with gold‐conjugated secondary antibody (1:100 in PBS) for 20 min, followed by three additional PBS washes. All grids were negatively stained with 2% uranyl acetate alternative for 20 s and air‐dried for at least 30 min in a fume hood. TEM images were observed using a JEM‐ARM200F device (JEOL, Tokyo, Japan). For SEM imaging on the ZAHVIS platform, the EVs were diluted 1:10 in PBS and deposited as droplets on a silicon wafer, which was then incubated at 37°C for 30 min. The wafer was fixed with 2.5% glutaraldehyde for 10 min and subsequently immersed in different concentrations of ethanol (30%, 50%, 70%, 80%, 90% and 100%) for 15 min each. After drying in a fume hood, a thin layer of platinum (Pt) was applied to the wafers, and the images were captured using a JSM‐7610F‐Plus device (JEOL). To determine the quantity, diameter distribution and surface properties of the EVs, we conducted NTA and zeta potential analysis following standard protocols. For NTA analysis, EVs obtained from ZAHVIS, UC and TEI were diluted 1:50 in PBS, while SEC‐isolated EVs were analysed without dilution. The prepared samples were injected into cuvettes and measured using the NS300 instrument (Malvern Panalytical, Malvern, UK). Data were processed with NanoSight software (Malvern Panalytical) to assess EV concentration and intensity distribution. The surface charge of the EVs was assessed using a Zetasizer Nano ZS90 instrument (Malvern Panalytical).

### 2.7 Western Blot Analysis

Proteins extracted from EVs isolated using the ZAHVIS platform, UC, TEI and SEC methods were analysed by western blotting. The protein concentration was determined using the Bradford assay, with a series of BSA dilutions as the standard. Equal amounts of protein (20 µg) were separated on a 10% SDS‐PAGE gel and transferred to a PVDF microporous membrane. The membrane was blocked for 1 h in PBS‐Tween 20 with 5% skim milk. Primary antibodies (CD9, CD81, CD63, Calnexin, GRP78, GM130, ApoB, ApoE and HSA) were diluted according to the manufacturer's instructions and incubated overnight at 4°C. The membranes were then incubated with HRP‐tagged secondary antibodies diluted as per the manufacturer's instructions for 1 h. Marker proteins were detected using a mixture of peroxidase and chemiluminescent substrate, and images were captured using a ChemiDoc MP Imaging System (Bio‐Rad, Hercules, CA, USA) and Image Lab software (Bio‐Rad).

### 2.8 Real‐Time PCR and RQ Values

The miRNA and circRNA biomarker candidates were normalized using small nuclear RNA U6 (U6 snRNA) and GAPDH, respectively. For miRNAs, the forward primer consisted of the miRNA sequence, and the reverse primer was the mRQ 3' Primer provided by TAKARA. For circRNAs, primers were synthesized and used based on the CircInteractome database and the NCBI primer design tool. All primer sets are listed in Table S1. For the synthesis of miRNA cDNA and real‐time PCR analysis, we have utilized the Mir‐X miRNA qRT‐PCR TB Green Kit. We have combined 4 µL of EV‐derived RNAs from the ZAHVIS sample with 5 µL of reaction buffer and 1 µL of reverse transcription enzyme. This mixture was incubated at 37°C for 1 h and then the enzyme was inactivated by heating at 85°C for 5 min. Subsequently, 90 µL of RNase‐free water was added to the mixture to obtain a final volume of 100 µL of synthesized cDNA, which was stored at −20°C for future use. For the real‐time PCR of miRNA, 2 µL of synthesized cDNA was combined with 0.5 µL of 10 µM miRNA‐specific forward primer, 0.5 µL of 10 µM mRQ 3′ reverse primer, 12.5 µL of TB green premix and 9.5 µL of RNase‐free water. The amplification protocol included an initial denaturation step at 95°C for 10 s, followed by 40 cycles of 5 s at 95°C and 20 s at 60°C, and concluded with a final melt curve stage to confirm the specificity of the PCR products. For the synthesis of cDNA from circRNAs, we employed the SuperScript IV First‐Strand Synthesis System. Initially, 11 µL of EV‐derived RNAs from the ZAHVIS sample were mixed with the reaction buffer and reverse transcription enzyme, creating a 20 µL reaction mixture. This mixture was incubated at 23°C for 10 min to facilitate primer annealing, followed by incubation at 50°C for 10 min to allow reverse transcription. The reaction was then terminated by heating at 80°C for 10 min to inactivate the reverse transcriptase. To the synthesized cDNA, we added 30 µL of RNase‐free water, resulting in a final volume of 50 µL. The cDNA was subsequently stored at −20°C until further use. For the real‐time PCR analysis of circRNA, 5 µL of synthesized cDNA was combined with circRNA‐specific primer sets and the Brilliant III SYBR Green QPCR Master Mix in a 20 µL reaction volume. The qPCR amplification protocol began with an initial denaturation step at 95°C for 2 min. This was followed by 50 cycles of denaturation at 95°C for 15 s, annealing at 60°C for 30 s and extension at 72°C for 1 min. To verify the specificity of the PCR products, a final melt curve analysis was performed.

The expression levels of miRNAs and circRNAs were quantified using comparative Ct values. The RQ value of gene expression was determined through the following steps. First, the ΔCt value was calculated by subtracting the Ct value of the reference gene (U6 for miRNAs and GAPDH for circRNAs) from the Ct value of the biomarker candidates. This was followed by calculating the ΔΔCt value by subtracting the average ΔCt of the control group from the ΔCt of the test samples. Finally, the RQ value was computed using the equation RQ = 2^−ΔΔCt^. For CRC and HC, the calculations were as follows:

(1) $$\Delta \mathrm{Ct}=\mathrm{Ct}\;\left(\mathrm{Biomarker}\;\mathrm{candidates}\right)-\mathrm{Ct}\;\left(U6\;\mathrm{or}\;\mathrm{GAPDH}\right)$$

(2) $$\Delta \Delta \mathrm{Ct}=\Delta \mathrm{Ct}\;\left(\mathrm{CRC}\;\mathrm{and}\;\mathrm{HC}\right)-\Delta \mathrm{Ct}\;\left(\mathrm{Average}\;\mathrm{of}\;\mathrm{HC}\right)$$

(3) $$\mathrm{Relative}\;\mathrm{quantification}\;\left(\mathrm{RQ}\right)\;\mathrm{value}=2^{-\Delta \Delta \mathrm{Ct}}$$

### 2.9 Collection of Blood Plasma Samples

The clinical validation of the ZAHVIS platform was conducted following the approval of the Institutional Review Board of Asan Medical Centre (IRB no. 2023‐0484), with all participants providing informed consent prior to inclusion. A total of 100 blood plasma samples and data were obtained from Asan Bio‐Resource Centre, Korea Biobank Network (2021‐13(230)). These samples were primarily focused on CEA levels and EV‐derived biomarkers, without data on other traditional CRC biomarkers such as CA19‐9 or CA125, and dietary information was not available. Blood from patients scheduled for surgery was collected in citrate tubes 1 day before the operation, typically in two tubes (5cc each). The collected blood was centrifuged at 4°C and 3000 rpm (1900 g) for 10 min using a Hanil Combi‐514R Refrigerated Centrifuge (Hanil Science, Gimpo, South Korea). Subsequently, 1.5 mL of the upper plasma layer was transferred to a new tube and centrifuged again at 4°C and 13,100 rpm (16,000 g) for 10 min using a Microcentrifuge‐5415R (Eppendorf, Hamburg, Germany). Approximately 1 mL of the upper layer of the centrifuged plasma was then transferred to a cryotube for freezing. Blood plasma samples from healthy participants visiting the Health Screening & Promotion Centre were also obtained from the BRC of Asan Medical Centre. All samples were stored in a liquid nitrogen tank (LN_2_ tank) at −196°C for preservation. The enrolled subjects included individuals with histologically or cytologically confirmed HC (*n* = 20), CRC stages 0–1 (*n* = 20), CRC stage 2 (*n* = 20), CRC stage 3 (*n* = 20) and CRC stage 4 (*n* = 20). These samples were properly classified and utilized for subsequent analysis.

### 2.10 AI‐Driven Analysis

We employed a deep learning‐based AI‐driven analysis to evaluate the diagnostic performance of various biomarker combinations. The analysis was conducted using Python 3.11.4 with libraries such as TensorFlow (2.14.0), Keras (2.14.0), NumPy (1.24.3), Pandas (2.1.1), scikit‐learn (1.3.0) and Matplotlib (3.7.1). The dataset was preprocessed by converting the CRC stages and HC columns to binary labels and normalizing the features using Z‐score standardization. To ensure representative class distribution, samples were divided into training (70%) and test (30%) sets using an optimized splitting method. The optimal split for each group (HC, CRC stages 0–1, stage 2, stage 3, stage 4) was determined by evaluating the mean squared error of feature means and standard deviations between training and test sets over 1000 iterations, aiming to minimize the difference. Various biomarker combinations were evaluated using a trained neural network model. The model architecture consisted of an input layer, two hidden layers with dropout regularization, and an output layer with sigmoid activation. Hyperparameters were optimized through grid search and *K*‐fold (*K* = 5) cross‐validation. The optimization process involved exploring a range of hyperparameters, including learning rates (0.0001, 0.001, 0.01, 0.1), batch sizes (4, 8, 16, 32), dense units (16, 32, 64, 128) and dropout rates (0.1, 0.2, 0.3, 0.4). The performance of each hyperparameter combination was evaluated using the validation loss and AUC metrics. Early stopping was implemented to prevent overfitting, and the best‐performing hyperparameters were selected based on the highest combined metric of (1−average validation loss) and average AUC. The selected hyperparameters included the Adam optimizer with a learning rate of 0.1, a batch size of 16, 128 dense units for the first hidden layer, and a dropout rate of 0.1. The second hidden layer was set to half the size of the first hidden layer, and both hidden layers included the same dropout rate. ROC curves were plotted for test datasets to visualize diagnostic performance, and AUC values were confirmed. The 95% confidence intervals (CI) for the AUC values were calculated using the bootstrap method with 1000 resampled datasets. To evaluate the diagnostic performance on the test set, performance metrics such as sensitivity, specificity, accuracy and F1 score were calculated based on Youden's index. This comprehensive analysis identified the best‐performing combinations. The methodology was consistently applied to analyse overall stages, early‐stage (stages 0–2), advanced‐stage (stages 3–4) and individual stages (stages 0–1, 2, 3 and 4).

### 2.11 Statistical Analysis

Statistical analyses were performed using IBM SPSS Statistics version 27.0 (IBM Corp., Armonk, NY, USA), GraphPad Prism version 10.2.3 (GraphPad Software, La Jolla, CA, USA) and Python. Continuous variables were compared using the Student's *t*‐test for normally distributed data and the Mann–Whitney *U*‐test for non‐normally distributed data, as determined by the Shapiro–Wilk and Kolmogorov–Smirnov tests. For all statistical tests, *p* values less than 0.05 were considered statistically significant. Python was utilized to compute the ROC curve and the AUC using the Scikit‐learn library, with the ROC curve visualized using the Matplotlib library. Additionally, performance metrics, including sensitivity, specificity, accuracy and F1 score, were calculated based on Youden's index using the Scikit‐learn library. The t‐SNE analysis was performed using five combined markers as the input for binary classification (CRC and HC), and the t‐SNE visualization was created using the Matplotlib library.

## 3 Results

### 3.1 Design of the ZAHV‐AI System

**FIGURE 1:**
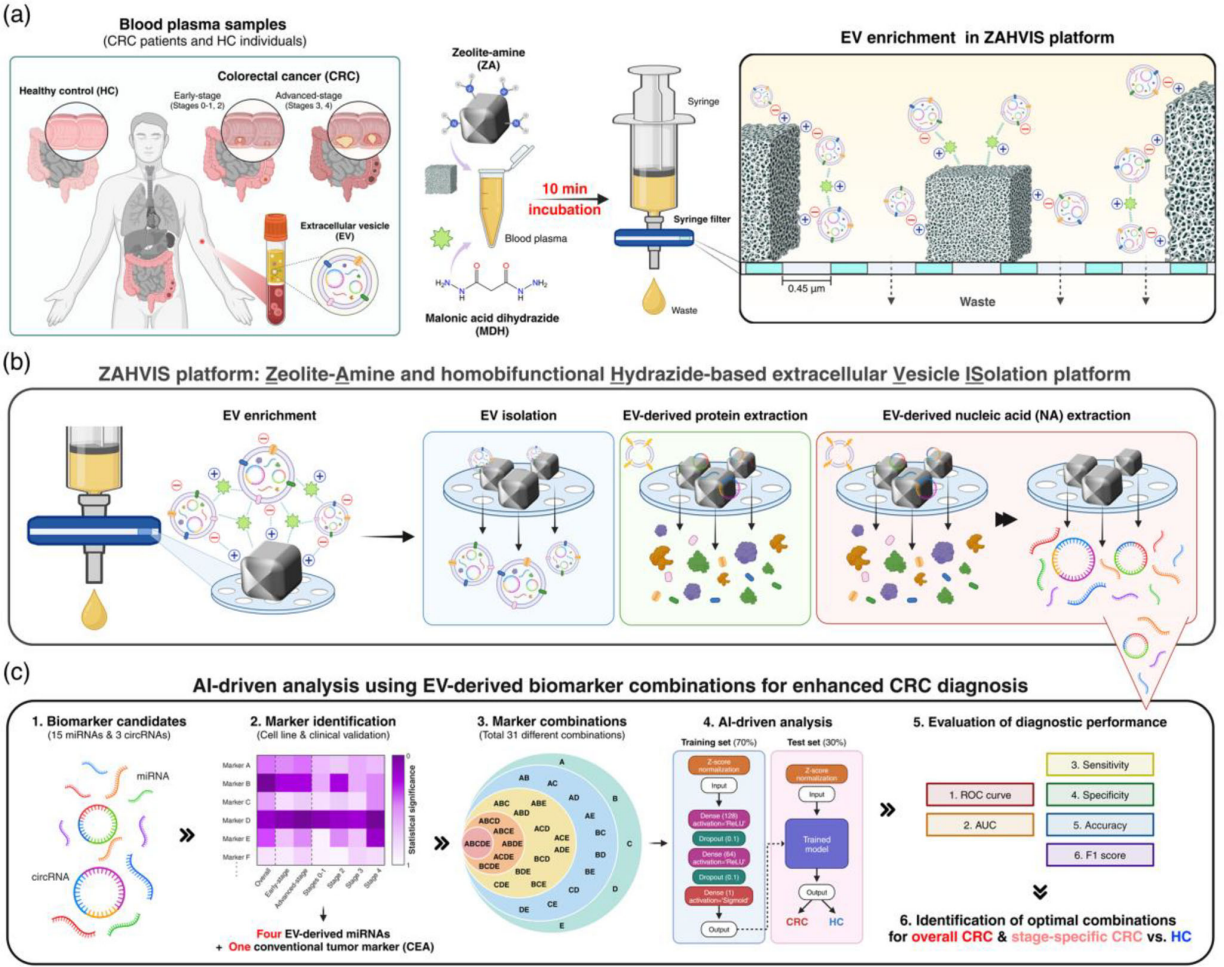
Overview of the ZAHV‐AI system. The ZAHV‐AI system integrates the ZAHVIS platform for efficient EV enrichment, isolation, and extraction of proteins and NAs, coupled with AI‐driven analysis to identify biomarker combinations for enhanced CRC diagnosis. Created with BioRender.com. (a) EV enrichment process. Blood plasma from CRC patients and HC individuals is incubated with zeolite‐amine (ZA) and malonic acid dihydrazide (MDH) for 10 min. This allows EVs to attach to the ZA surface via electrostatic and covalent interactions. The mixture is then filtered to capture ZA‐bound EVs. (b) EV isolation and molecular extraction for downstream analysis. Post‐enrichment, the ZAHVIS platform facilitates EV isolation and the extraction of components, specifically EV‐derived proteins and NAs, providing a streamlined approach for comprehensive EV analysis. (c) AI‐driven biomarker analysis. RNA extracted from EVs undergoes analysis to identify significant miRNA and circRNA biomarkers, leading to the selection of four miRNAs. These miRNAs, along with the conventional CEA, are evaluated across 31 combinations using AI with the dataset split into training (70%) and test (30%) sets. Metrics such as receiver operating characteristic (ROC) curve, area under the curve (AUC), sensitivity, specificity, accuracy, and F1 score are used to evaluate diagnostic performance and determine the optimal combinations for overall CRC and stage‐specific CRC (early‐stage, advanced‐stage, and specific stages 0–1, 2, 3, 4).

The ZAHV‐AI system is designed to enhance the diagnosis and monitoring of CRC by leveraging biomarkers derived from EVs in liquid biopsy samples. This innovative system integrates the ZAHVIS platform for efficient EV isolation with deep learning‐based AI‐driven analysis to determine the optimal blood biomarker combinations (Figure 1). The workflow begins with the preparation of blood plasma samples from CRC patients at various stages (early‐stage: stages 0–2; advanced‐stage: stages 3–4) and HC individuals. The ZAHVIS platform initiates the process by adding ZA and MDH to the plasma samples, followed by a 10‐min incubation period to facilitate rapid and efficient EV capture. The mixture is then passed through a syringe filter to further enrich the EVs (Figure 1a). The ZAHVIS platform isolates the EVs or extracts critical components such as EV‐derived proteins and NAs to prepare for subsequent steps. This stepwise approach ensures a comprehensive and streamlined analysis of the crucial elements contained within the EVs, significantly enhancing the overall efficiency of the process (Figure 1b). The ZAHV‐AI system incorporates the AI‐driven analysis of EV‐derived biomarker combinations to aid in CRC diagnosis. Initially, we selected 15 miRNAs and three circRNAs as potential biomarker candidates based on previous studies indicating their association with CRC. Through rigorous validation using cell line models and clinical samples, four significant miRNA markers were identified. These validated markers, along with the conventional CEA tumour marker, were combined into 31 different combinations for further analysis. The AI‐driven component evaluates these combinations using receiver operating characteristic (ROC) curves and AUC values, along with performance metrics such as sensitivity, specificity, accuracy and F1 scores. This comprehensive evaluation identifies the most effective combinations for distinguishing CRC from HC, as well as for differentiating among various CRC stages (overall CRC, early‐stage, advanced‐stage and individual stages [stages 0–1, 2, 3 and 4]) (Figure 1c). The ZAHV‐AI system's ability to integrate efficient EV isolation with AI‐driven analysis represents a significant advancement in CRC diagnostics. This integration provides a streamlined, efficient and highly sensitive approach for the early detection and monitoring of CRC using blood‐based EV‐derived biomarkers, offering a robust tool for improving patient outcomes.

### 3.2 Implementation and Working Principle of the ZAHVIS Platform

The ZAHVIS platform is engineered to efficiently isolate EVs from biological samples by utilizing ZA and MDH as functional materials (Figure S1A). It's effectiveness in EV capture and isolation is based on a robust bonding mechanism facilitated by these two key components. ZA, derived from zeolites, features a naturally porous aluminosilicate (Al_2_O_3_ and SiO_2_) structure with a significantly larger surface area compared to flat, uniform surfaces, which enhances interactions with EVs and leads to improved yield and purity (Li et al. 2017). As natural minerals, zeolites are abundant, cost‐effective and provide notable stability and uniformity, making them well‐suited for large‐scale applications. The negatively charged sites within the aluminosilicate framework allow for easy surface functionalization. By modifying the surface with amine groups (NH_2_), ZA gains a positive surface charge, which promotes electrostatic interactions with the negatively charged EV membranes. These properties, including high surface area and effective surface modification, lead to efficient EV capture and stable binding, making ZA highly effective for EV isolation. MDH, a soluble, low‐toxic and thermally and chemically stable material, functions as a crosslinking agent that significantly enhances the efficiency and precision of EV capture due to its enhanced binding and adsorption capacity. MDH contains hydrazide reactive groups (C = ONHNH_2_) at both ends, providing dual functionality. The positively charged hydrazine groups (NH‐NH_2_) facilitate electrostatic interactions with the negatively charged EVs. Additionally, the carbonyl components (C = O) of MDH form imine bonds (C = N) with the amines of ZA, resulting in a stable binding mechanism. This dual interaction—electrostatic and covalent—ensures the successful capture and secure binding of EVs to the ZA surface within just 10 min. Following EV capture by the ZA and MDH, the mixture undergoes syringe filtration. The ZA particles, larger than the syringe filter's pore size, retain the bound EVs while unbound molecules pass through as waste. This filtration process is quick, straightforward and cost‐effective, requiring minimal equipment, making it accessible for a wide range of laboratory settings.

The ZAHVIS platform is a one‐step solution that not only concentrates the EVs but also facilitates the isolation of EVs and the extraction of EV‐derived proteins and NAs (Figure S1B). To begin with, EV isolation uses a high pH elution buffer, causing the amine group of ZA and hydrazine group of MDH to lose their positive charge, detaching the EVs concentrated on the ZA surface. These separated EVs can then be easily filtered through the pores for isolation. As a result, EV isolation with the ZAHVIS platform is completed in under 15 min, resulting in high‐concentration, high‐purity EVs. For the extraction of EV‐derived proteins, the ZAHVIS platform uses the RIPA lysis buffer to dissolve EVs. These proteins, due to their non‐reactivity with ZA and MDH, remain suspended in the RIPA lysis buffer. Consequently, EV‐derived proteins can be readily obtained through a simple collection of the buffer within 30 min. To extract EV‐derived NAs, the ZAHVIS platform employs a non‐chaotropic NP‐40 lysis buffer instead of the typical RIPA or generic lysis buffers, effectively lysing EVs while preventing NA degradation caused by chaotropic reagents. Using non‐chaotropic lysis buffer, the ZAHVIS platform ensures the preservation of NA integrity, resulting in higher quality and yield for downstream applications. When EVs are dissolved, the released EV‐derived NAs can form bonds with ZA and MDH (Jeon et al. 2024; Koo, Kim, Lee, et al. 2023; Koo, Kim, Jang, et al. 2023). These bonds are facilitated by electrostatic interactions between the negatively charged EV‐derived NAs and the positively charged ZA and MDH. Additionally, the carbonyl groups of MDH form imine bonds with the amine groups of the nucleobases adenine (A), guanine (G) and cytosine (C), as well as the amine groups of the ZA. Similarly, the carbonyl groups of the nucleobases G, C, thymine (T, in DNA) and uracil (U, in RNA) form imine bonds with the amine groups of the ZA. Furthermore, the hydrazide groups of MDH form hydrazone bonds (–C = N–NH–) with the carbonyl groups of the nucleobases G, C, T and U. These interactions result in both electrostatic and covalent bonds, effectively trapping the EV‐derived NAs on the ZA surface. This method ensures a more stable capture of NAs, thereby enhancing the reliability and efficiency of the extraction process. The bonds between the extracted NAs and both ZA and MDH can be disrupted by the high pH elution buffer, facilitating EV‐derived NAs extraction within 35 min. The ZAHVIS platform enables the enrichment, isolation and extraction of EV‐derived components within remarkably short timeframes. This efficiency is largely due to the unique properties of ZA and MDH, which facilitate rapid and effective capture and isolation of EVs and their components, making the ZAHVIS platform a highly effective tool for cancer diagnostics and research.

### 3.3 Characterization, Optimization and Comparative Analysis of the ZAHVIS Platform

**FIGURE 2:**
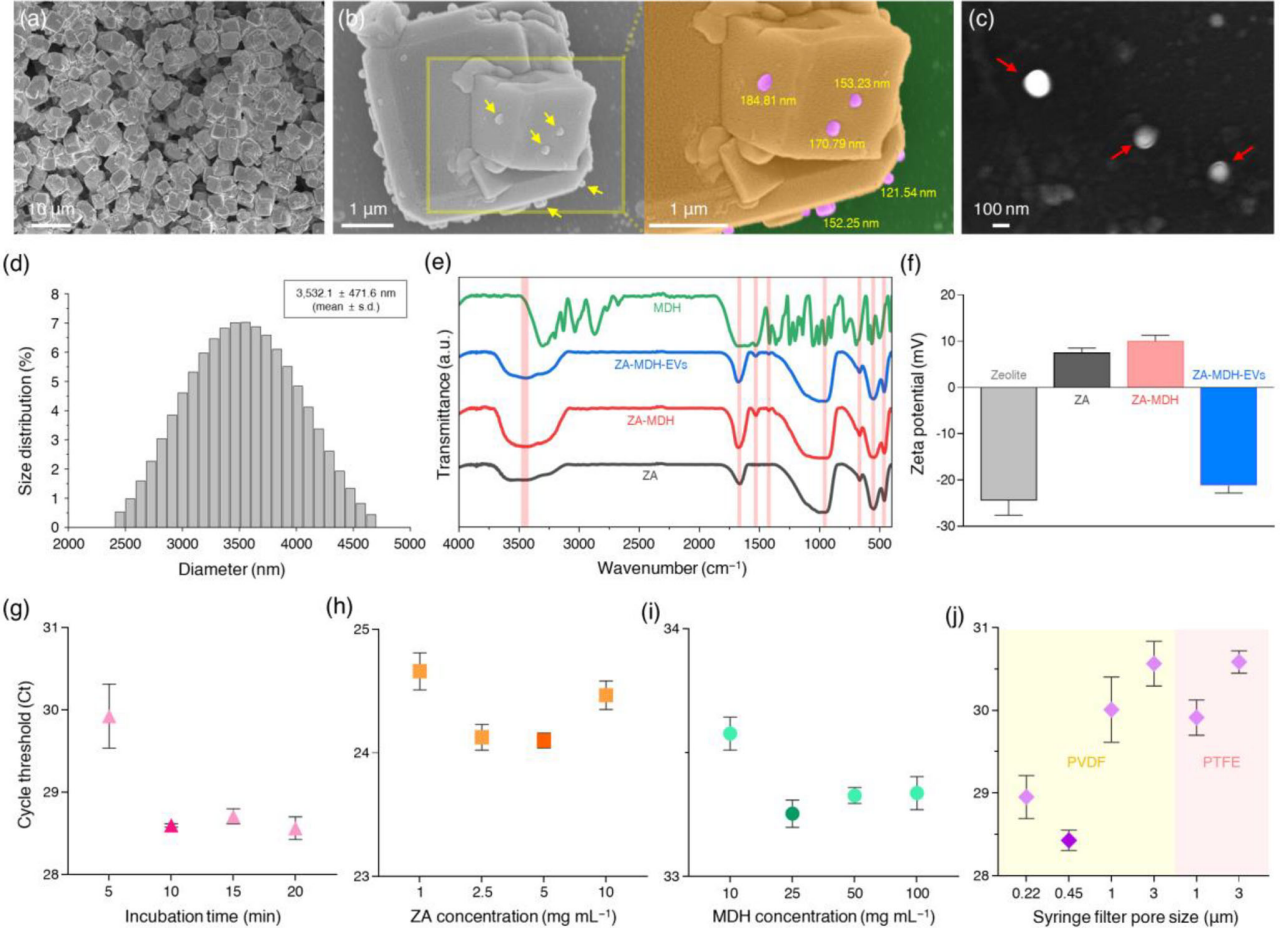
Characterization and optimization of the ZAHVIS platform. (a–c) Scanning electron microscopy (SEM) images of ZA particles (a) and ZA with captured EVs, shown in standard and magnified colored formats (b). The colored image highlights ZA (orange), captured EVs (pink), and the background (green). Isolated EVs (c). (d) Dynamic light scattering (DLS) analysis showing ZA particle size distribution. (e) Fourier‐transform infrared (FTIR) spectra for ZA, ZA modified with MDH (ZA‐MDH), EVs bound to ZA‐MDH (ZA‐MDH‐EVs), and MDH. (f) Zeta potential measurements for zeolite, ZA, ZA‐MDH, and ZA‐MDH‐EVs. (g–j) Optimization of incubation time for EV enrichment (g), ZA concentration (h), MDH concentration (i), and syringe filter pore size and type (j) using cycle threshold (Ct) values of miR‐21‐5p after EV enrichment and EV‐derived RNA extraction with the ZAHVIS platform. *n* = 3 or 4 biologically independent experiments per group. Bars and dots represent mean ± standard deviation (SD).

The characteristics of the ZAHVIS platform were confirmed through a series of analytical techniques, including scanning electron microscopy (SEM), dynamic light scattering (DLS), Fourier‐transform infrared (FTIR) spectroscopy and zeta potential analyses (Figure 2a–f). SEM images revealed the structural characteristics and interaction dynamics of EVs with ZA and MDH. Zeolite exhibited robust structural features even during amine modification and maintained stability (Figure 2a). Upon capturing EVs, the ZA displayed clear interactions with EVs, highlighted in both standard and magnified coloured formats, showing EV sizes ranging from 121.54 to 184.81 nm (Figure 2b). Additional SEM analysis confirmed the successful isolation of EVs (Figure 2c). DLS analysis provided the size distribution of ZA particles, which averaged 3532.1 ± 471.6 nm, indicating a relatively uniform size (Figure 2d). The FTIR spectra confirmed the chemical modifications on the ZA surface due to functionalization with 3‐Aminopropyl(diethoxy)methylsilane (APDMS) and MDH, as well as subsequent interactions with EVs, indicated by characteristic peaks at specific wavelengths (Figure 2e). The FTIR analysis of ZA revealed characteristic peaks of the zeolite structure at 461 cm^−1^ (symmetric bending of tetrahedrally bonded Si or Al), 550 cm^−1^ (symmetric stretching of double six‐membered rings), 665 cm^−1^ (symmetric stretching of Si‐O‐Si) and 974 cm^−1^ (asymmetric stretching of Si‐O‐Al and Si‐O‐Si), as well as peaks at 1665 cm^−1^ (NH bending) and 3462 cm^−1^ (NH stretching) due to the introduction of amine groups from APDMS. These peaks signify the successful modification of amine functionalities onto the zeolite surface. In the spectra for ZA modified with MDH (ZA‐MDH), additional peaks emerged at 1418 cm^−1^ (NH bending and CN stretching), 1533 cm^−1^ (CN stretching), along with increased intensities at 1665 cm^−1^ (NH bending) and a broad peak at 3462 cm^−1^ (NH stretching). These changes indicate that MDH reacted with ZA, and the characteristic peaks of MDH are now present on the ZA surface. Specifically, the enhanced intensity of the peak at 1533 cm^−1^ suggests the formation of imine bonds, as the carbonyl components of MDH form imine bonds with the amines of ZA, resulting in a stable binding mechanism. For EVs captured by ZA‐MDH (ZA‐MDH‐EVs), the FTIR spectra showed significant changes, especially in the peaks related to NH bending and stretching. The reduced intensity of the NH peaks (1665 and 3462 cm^−1^) confirms the effective capture and interaction of EVs with the ZA‐MDH. This suggests that EVs have successfully bound to the ZA‐MDH surface. Zeta potential measurements demonstrated significant surface charge changes through each stage of modification and EV capture (Figure 2f). The initial zeta potential of pure zeolite averaged −24.47 ± 3.17 mV, indicating a highly negative surface charge. The presence of hydroxyl groups (OH) on the zeolite surface enables functionalization with APDMS, leading to a zeta potential change to 7.51 ± 1.02 mV as amine groups are introduced. Further modification with MDH shifted the zeta potential to 9.99 ± 1.29 mV, reflecting additional surface changes. Finally, the zeta potential of ZA‐MDH‐EVs was −21.23 ± 1.59 mV, demonstrating effective interactions between ZA and negatively charged EVs through electrostatic binding mechanisms.

To determine the best conditions for efficient EV isolation using the ZAHVIS platform, several key parameters were optimized, including the incubation time for EV enrichment, the concentration of ZA and MDH, and the type and pore size of the syringe filter (Figure 2g–j). First, we optimized the incubation time for EV enrichment by testing durations of 5, 10, 15 and 20 min. The results showed a significant increase in efficiency at 10 min compared to 5 min, but the efficiency levelled off after 10 min, with similar results at 15 and 20 min (Figure 2g). This suggests that a 10‐min incubation time is sufficient for EV enrichment. Next, the concentration of ZA was evaluated. Efficiency increased as the amount of ZA rose from 1 to 5 mg mL^−1^ but decreased when the concentration further increased to 10 mg mL^−1^ (Figure 2h). Increasing the amount of ZA to 5 mg mL^−1^ enhances the surface area available for EV binding, while an excessive amount of ZA can decrease the opportunity for contact between ZA and EVs due to insufficient mixing. The concentration of MDH is crucial as it affects the binding strength between ZA and EVs. Isolation efficiency peaked at an MDH concentration of 25 mg mL^−1^ but decreased with further increases (Figure 2i). Lower MDH concentrations provide fewer hydrazide groups for effective binding to the EVs. However, at higher concentrations, excess MDH can act as an inhibitor by causing steric hindrance on the ZA surface, thereby reducing the ability of EVs to bind efficiently. We further evaluated the efficiency by the type (PVDF and PTFE) and pore size of the syringe filter (0.22, 0.45, 1, 3 µm) used in the ZAHVIS platform. As shown in Figure 2j, pore size influenced isolation efficiency more noticeably than filter material, with smaller pore sizes generally improving efficiency, while both PVDF and PTFE filters provided sufficient stability for EV isolation. However, the 0.22 µm filter demonstrated lower efficiency due to excessive pore constriction, leading to clogging and restricted flow, which hindered the isolation process. In contrast, the 3 µm filter exhibited lower efficiency than the 0.22, 0.45 and 1 µm filters because the small ZA particles, with a mean particle size of 3.5 µm, passed through the filter, reducing EV capture. The 0.45 µm PVDF filter achieved the best balance between particle retention and flow, ensuring efficient EV isolation. Based on these results, the optimal conditions were identified as a 10‐min incubation time, 5 mg mL^−1^ ZA, 25 mg mL^−1^ MDH, and the use of a 0.45 µm PVDF syringe filter. These parameters were applied in all subsequent experiments to ensure consistent efficiency and reliability in EV isolation and analysis.

To further assess the contributions of electrostatic interaction and covalent bonding in EV isolation, we compared EV recovery between ZA alone, which relies solely on electrostatic interaction, and ZA and MDH, which incorporates both electrostatic interaction and covalent bonding (Figure S2). The results showed that the addition of MDH significantly improved EV recovery, with EV concentrations of 9.77 × 10^7^ particles mL^−1^ for ZA alone and 1.58 × 10^8^ particles mL^−1^ for the combination of ZA and MDH, compared to the initial sample at 1.80 × 10^8^ particles mL^−1^. Statistical analysis confirmed significant differences between the initial sample and ZA alone (**, *p* = 0.0031) and between ZA alone and the combination of ZA and MDH (**, *p* = 0.0071), while no significant difference was observed between the initial sample and the combination of ZA and MDH (ns, *p* = 0.1306). These findings demonstrate that covalent bonding, mediated by MDH, enhances EV retention and recovery within the optimized 10‐min incubation period. MDH also functions as a cross‐linker, stabilizing EV capture and minimizing loss. This highlights the advantage of the dual isolation mechanism, distinguishing the ZAHVIS platform from methods based solely on electrostatic interaction. In addition, the reproducibility of the ZAHVIS platform in biomarker detection was evaluated by assessing the linearity of miRNA quantification using serial dilutions of plasma samples (Figure S3). The results showed a linear correlation between sample dilution and miRNA levels, with an *R* ^2^ value of 0.9765. These findings demonstrate that ZAHVIS enables efficient and reproducible EV isolation through a dual mechanism under optimized conditions.

**FIGURE 3:**
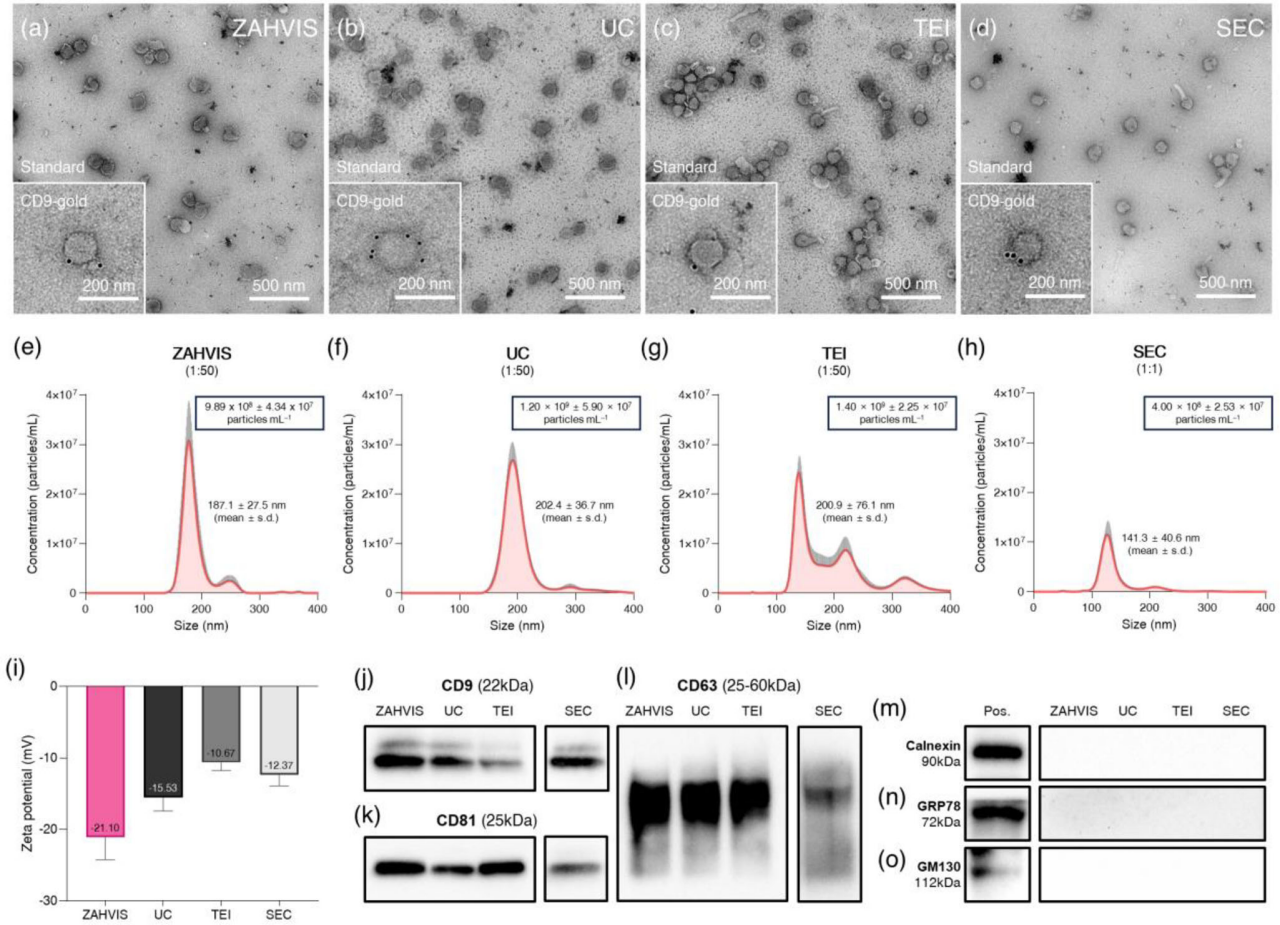
Characterization of EVs isolated by the ZAHVIS platform compared to traditional methods. (a–d) Transmission electron microscopy (TEM) images of EVs isolated using the ZAHVIS platform (a), ultracentrifugation (UC) (b), total exosome isolation (TEI) (c), and size exclusion chromatography (SEC) (d). Each panel includes both standard TEM and CD9‐gold labelling to confirm EV presence. (e–h) Nanoparticle tracking analysis (NTA) results displaying the size distribution and concentration of EVs isolated using ZAHVIS (e), UC (f), TEI (g), and SEC (h). (i) Zeta potential measurements of EVs isolated using the same methods. (j–o) Western blot analysis of EV‐derived proteins. EVs were concentrated and proteins extracted in a one‐step process using the ZAHVIS platform. UC, TEI and SEC methods involved first isolating EVs followed by protein extraction. The EV markers include CD9 (j), CD81 (k) and CD63 (l), while the non‐EV markers include Calnexin (endoplasmic reticulum (ER) marker) (m), Glucose‐regulated protein 78 (GRP78, ER stress marker) (n) and Golgi matrix protein 130 (GM130, Golgi marker) (o). Positive control (Pos.) results are shown using lysates from the HCT116 cell line. Scale bars in TEM images represent 500 nm or 200 nm. Bars represent mean ± SD

The performance of the ZAHVIS platform was evaluated through comparisons with commonly employed EV isolation techniques, specifically UC, TEI and SEC methods (Figure 3). The morphology of EVs isolated by each method was verified using TEM images (Figure 3a–d). All methods revealed spherical EVs within the typical size range, confirming structural integrity. Standard TEM and CD9‐gold immunolabeling images confirmed successful EV capture across all methods. In addition, EV size distribution and concentration were evaluated using NTA (Figure 3e–h). The mean particle sizes were 187.1 ± 27.5 nm for ZAHVIS, 202.4 ± 36.7 nm for UC, 200.9 ± 76.1 nm for TEI, and 141.3 ± 40.6 nm for SEC. The NTA results, measured at a 1:50 dilution, showed EV concentrations of 9.89 × 10^8^ ± 4.34 × 10^7^ particles mL^−1^ for ZAHVIS, 1.20 × 10^9^ ± 5.90 × 10^7^ particles mL^−1^ for UC, and 1.40 × 10^9^ ± 2.25 × 10^7^ particles mL^−1^ for TEI, while SEC, measured without dilution, yielded 4.00 × 10^8^ ± 2.53 × 10^7^ particles mL^−1^. These results indicate that ZAHVIS yields slightly fewer EVs than UC and TEI, while SEC shows the lowest recovery. To further evaluate the functional efficiency of ZAHVIS, we compared EV‐derived RNA recovery across the different isolation methods (Figure S4). The one‐step ZAHVIS approach, which integrates EV enrichment and RNA extraction in a single workflow, was evaluated alongside conventional workflows, where EVs were first isolated using ZAHVIS, UC, TEI or SEC, followed by RNA extraction using a commercial kit. The results showed that the one‐step ZAHVIS method yielded the highest RNA recovery, with a Ct value of 26.06, compared to 28.99 for ZAHVIS with the commercial kit, 28.20 for UC, 27.46 for TEI, and 34.28 for SEC. These findings suggest that the one‐step ZAHVIS approach minimizes sample loss and enhances RNA recovery by integrating EV enrichment and RNA extraction, compensating for its slightly lower EV yield compared to UC and TEI. Additionally, zeta potential measurements were performed to examine the surface properties of the isolated EVs (Figure 3i). The EVs exhibited negative surface charges, with values of −21.10 ± 3.15 mV for ZAHVIS, −15.53 ± 1.91 mV for UC, −10.67 ± 1.12 mV for TEI, and −12.37 ± 1.55 mV for SEC, confirming surface stability across all methods. These findings confirm that ZAHVIS achieves EV yields comparable to conventional methods while maintaining consistent RNA recovery suitable for molecular applications.

To further validate the ZAHVIS platform, we performed western blot analysis to assess the quantity and purity of EV‐derived proteins, as well as the selectivity and reproducibility of the isolation process. The EV‐specific proteins CD9, CD81 and CD63 were detected in EVs isolated by ZAHVIS, UC, TEI and SEC (Figure 3j–l). SEC, which produced fewer EVs, was analysed separately under the same conditions to confirm the presence of these markers. Non‐EV markers such as Calnexin, GRP78 and GM130 were consistently absent across all methods, demonstrating the effective removal of cellular contaminants (Figure 3m–o). In addition, we examined the removal of blood‐derived contaminants such as Apolipoprotein B (ApoB), Apolipoprotein E (ApoE) and human serum albumin (HSA) (Figure S5). The original plasma sample exhibited detectable bands for ApoB, ApoE and HSA. However, ApoE and HSA were not detected in EVs isolated using ZAHVIS, UC and TEI, confirming their effective removal across these methods. ApoB was present at reduced levels, with ZAHVIS producing weaker bands compared to TEI, while showing similar selectivity to UC. These results suggest that ZAHVIS effectively minimizes plasma protein contamination, contributing to higher EV purity. To further assess the removal efficiency of lipoprotein‐associated contaminants, we conducted a controlled study by spiking EV fractions with ApoB (Figure S6). The results showed that SEC achieved the highest purity, with no detectable ApoB band, while ZAHVIS, UC and TEI exhibited comparable residual ApoB signals. These findings suggest that ZAHVIS achieves slightly lower removal efficiency than SEC, with comparable performance to UC and TEI, while also maintaining high EV yield and providing high‐purity EVs suitable for downstream molecular analyses. Therefore, the ZAHVIS platform demonstrates high selectivity in EV isolation, effectively minimizing contamination and ensuring reliable EV recovery for molecular applications, making it a robust alternative to conventional methods.

### 3.4 Assessment of EV‐Derived Biomarker Candidates Using Cell Line Models

**FIGURE 4:**
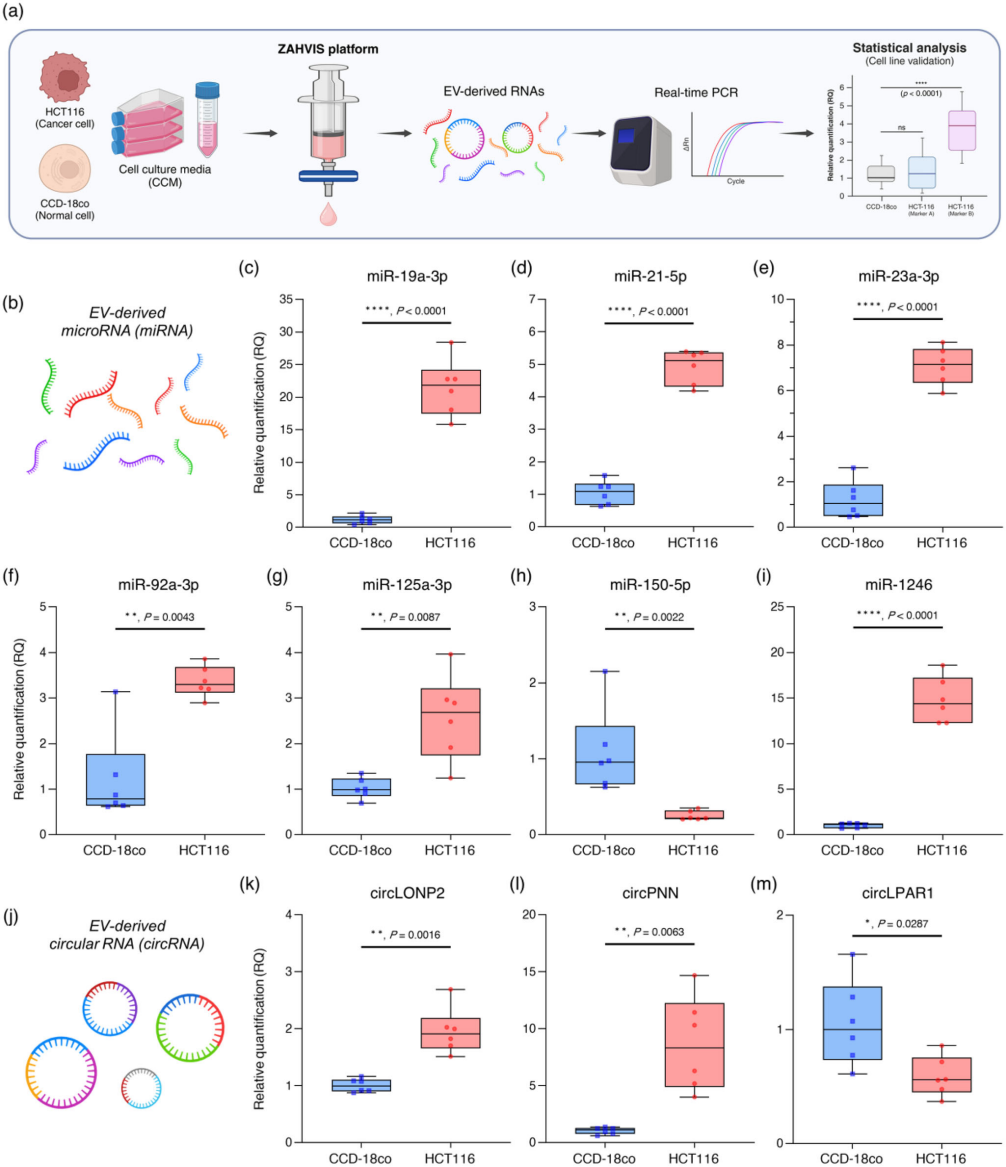
Validation of biomarker candidates using cell line models with the ZAHVIS platform. (a) Schematic overview of using cell line models to validate biomarker candidates. The ZAHVIS platform extracts EV‐derived RNAs from the cell culture media of HCT116 (cancer cells, *n* = 6) and CCD‐18co (normal cells, *n* = 6). These RNAs are then analysed using real‐time PCR to quantify biomarker candidates and control genes, followed by statistical analysis to determine relative quantification (RQ) values and significance. Created with BioRender.com. (b) Illustration of EV‐derived miRNAs. Created with BioRender.com. (c–i) RQ values of statistically significant miRNAs identified in cell line models including miR‐19a‐3p (c), miR‐21‐5p (d), miR‐23a‐3p (e), miR‐92a‐3p (f), miR‐125a‐3p (g), miR‐150‐5p (h) and miR‐1246 (i). (j) Illustration of EV‐derived circRNAs. Created with BioRender.com. (k–m) RQ values of statistically significant circRNAs identified in cell line models including circLONP2 (k), circPNN (l) and circLPAR1 (m). Box plots represent the median, interquartile range (IQR) and whiskers indicating the minimum and maximum values. Each dot represents an individual sample. Statistical significance indicated by *p < 0.05, **p < 0.01, ***p < 0.001 and ****p < 0.0001.

To validate potential biomarker candidates for CRC diagnosis, we employed cell line models in conjunction with the ZAHVIS platform (Figure 4). The experimental approach is schematically represented in Figure 4a. EV‐derived RNAs were extracted from the cell culture media of HCT116 (cancer cells) and CCD‐18co (normal cells) using the ZAHVIS platform. These RNAs were analysed using real‐time PCR to quantify both biomarker candidates and control genes (Table S1). This quantification allowed for the determination of RQ values, followed by statistical analysis to evaluate the significance of these markers. Figure 4b illustrates the EV‐derived miRNAs. Among the 15 miRNAs investigated, seven miRNAs were identified as statistically significant, including miR‐19a‐3p, miR‐21a‐5p, miR‐23a‐3p, miR‐92a‐3p, miR‐125a‐3p, miR‐150‐5p and miR‐1246 (Figure 4c–i). The RQ values for these miRNAs demonstrated distinct expression patterns between the HCT116 and the CCD‐18co cell line, indicating their potential as biomarkers for CRC. In addition to miRNAs, we also analysed circRNAs derived from EVs (Figure 4j). Among the three circRNAs examined, three were found to be statistically significant including circLONP2, circPNN and circLPAR1 (Figure 4k–m). The RQ values for these circRNAs also exhibited significant differences between the cancer and normal cell lines, further supporting their potential as CRC biomarkers. Furthermore, the statistically non‐significant biomarkers were identified, which included miR‐99b‐5p, miR‐122‐5p, miR‐141‐3p, miR‐181a‐5p, miR‐182‐5p, miR‐200b‐3p, miR‐222‐5p and miR‐223‐3p (Figure S7). These findings demonstrate the ZAHVIS platform's effectiveness in isolating and analysing EV‐derived NAs, confirming the identified miRNAs and circRNAs as promising CRC biomarkers. By validating these biomarkers in cell line models, we have established a solid foundation for their potential use in clinical settings.

### 3.5 Clinical Validation of Selected EV‐Derived Biomarkers in Blood Plasma

**FIGURE 5:**
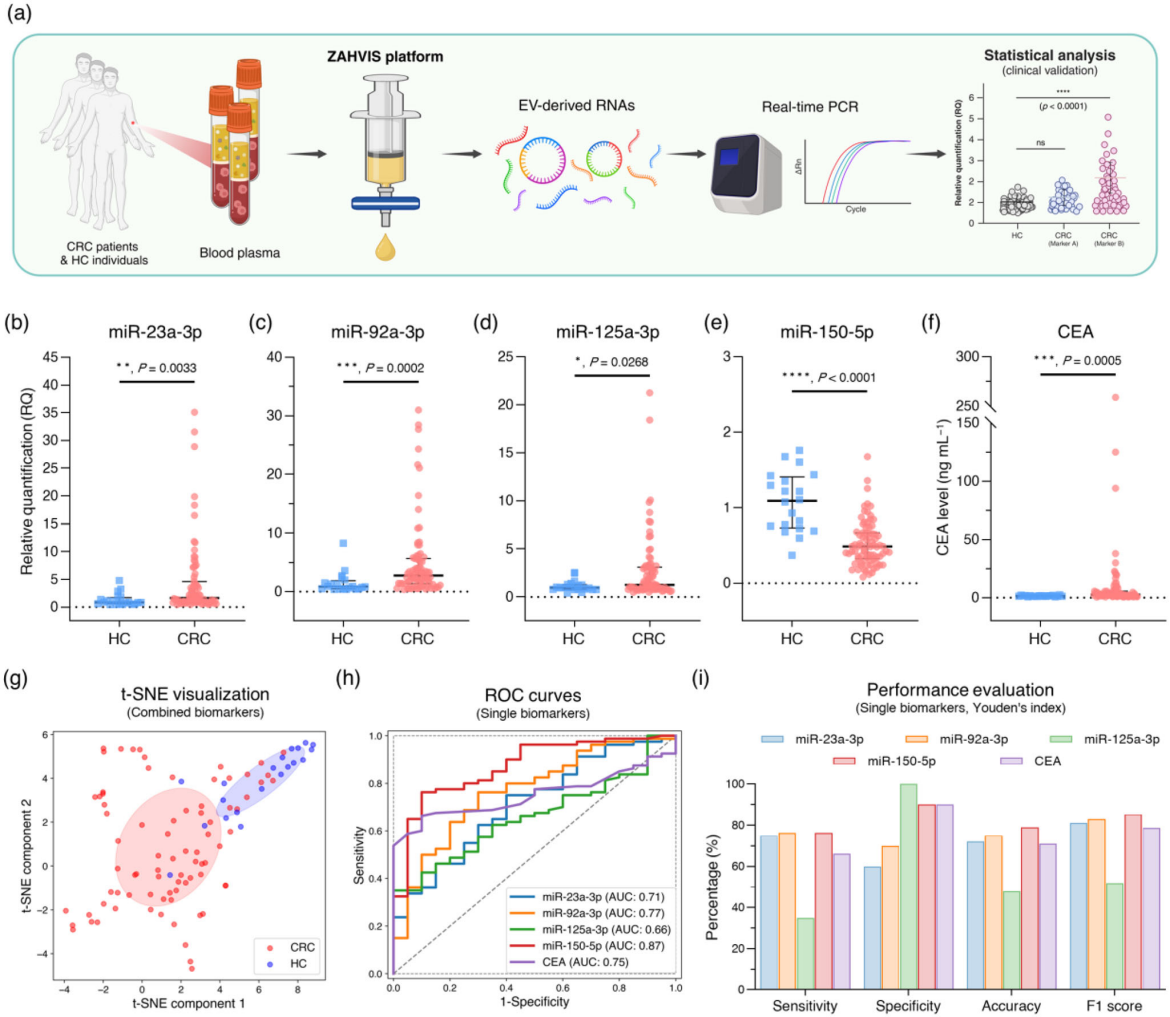
Clinical validation of biomarkers identified in cell line models using blood plasma samples with the ZAHVIS platform. (a) Schematic overview of using clinical samples to validate biomarkers identified in cell line models. The ZAHVIS platform extracts EV‐derived RNAs from blood plasma samples of CRC patients and HC individuals. These RNAs are analysed using real‐time PCR to quantify biomarkers and control genes, followed by statistical analysis to determine RQ values and significance. Created with BioRender.com. (b–e) RQ values of clinically validated miRNAs in CRC patients (*n* = 80) and HC individuals (*n* = 20) including miR‐23a‐3p (b), miR‐92a‐3p (c), miR‐125a‐3p (d) and miR‐150‐5p (e). These miRNAs were selected from a pool of seven miRNAs and three circRNAs based on their statistical significance in both cell line and clinical experiments. (f) CEA levels in CRC patients and HC individuals. (g) t‐Distributed stochastic neighbour embedding (t‐SNE) visualization of combined biomarkers discriminating between CRC patients and HC individuals. (h) ROC curves for single biomarkers illustrating their diagnostic performance with corresponding AUC values. (i) Performance evaluation of single biomarkers using Youden's index, including sensitivity, specificity, accuracy and F1 score. Dot plots represent individual samples, with a line at the median and IQR. Statistical significance indicated by *p < 0.05, **p < 0.01, ***p < 0.001 and ****p < 0.0001.

To confirm the clinical relevance of biomarkers identified through cell line models, we analysed blood plasma samples from CRC patients (*n* = 80) and HC individuals (*n* = 20) using the ZAHVIS platform (Figure 5 and Table 1). The clinical characteristics of CRC patients were analysed to determine their disease‐free survival probabilities over 100 months (Figure S8). Survival rates were observed to be 95% for early‐stage CRC, 75% for advanced‐stage CRC, 100% for CRC stages 0–1, 90% for CRC stage 2, 80% for CRC stage 3 and 70% for CRC stage 4. The experimental approach is schematically represented in Figure 5a. This process involved the extraction of EV‐derived RNAs from the blood plasma samples, followed by real‐time PCR quantification of the seven miRNAs and three circRNAs identified from cell line models, as well as control genes. The data were then subjected to statistical analysis to determine the RQ values and their significance. The results showed significant differences in the expression levels of miR‐23a‐3p, miR‐92a‐3p, miR‐125a‐3p and miR‐150‐5p between CRC patients and HC individuals, indicating their potential as diagnostic biomarkers for CRC (Figure 5b–e). Several biomarkers did not show statistically significant differences in clinical samples, including miR‐19a‐3p, miR‐21‐5p, miR‐1246, circLONP2, circPNN and circLPAR1 (Figure S9). We also measured CEA levels and found them to be significantly higher in CRC patients compared to HC individuals, confirming CEA's established role as a diagnostic marker for CRC (Figure 5f).

**TABLE 1:** Clinical sample information used in the study.

	CRC							
Case	Overall	Early‐stage	Advanced‐stage	Stages 0–1	Stage 2	Stage 3	Stage 4	HC
	80	40	40	20	20	20	20	20
**Age**								
Median	60	62.5	60	66.5	59	59.5	60	48.5
Range	32–77	36–77	32–77	36–77	41–74	32–77	35–77	31–61
**Sex**								
Male	50 (62.5%)	25 (62.5%)	25 (62.5%)	12 (60%)	13 (65%)	14 (70%)	11 (55%)	13 (65%)
Female	30 (37.5%)	15 (37.5%)	15 (37.5%)	8 (40%)	7 (35%)	6 (30%)	9 (45%)	7 (35%)
**EV‐derived blood biomarker [median (range)]**								
miR‐23a‐3p	1.65 (0.42–35.08)	2.12 (0.42–31.54)	1.53 (0.6–35.08)	2.12 (0.42–19.87)	1.81 (0.47–31.54)	1.54 (0.73–7.56)	1.53 (0.6–35.08)	0.85 (0.44–4.8)
miR‐92a‐3p	2.78 (0.36–31.01)	2.21 (0.5–31.01)	3.09 (0.36–21.08)	1.82 (0.51–31.01)	3.36 (0.5–28.47)	2.80 (0.48–8.37)	3.53 (0.36–21.08)	0.86 (0.37–8.27)
miR‐125a‐3p	1.26 (0.47–21.24)	1.27 (0.51–21.24)	1.26 (0.47–9.84)	1.07 (0.6–6.27)	1.77 (0.51–21.24)	1.07 (0.7–7.85)	2.49 (0.47–9.84)	0.96 (0.41–2.54)
miR‐150‐5p	0.49 (0.08–1.68)	0.43 (0.18–1.68)	0.50 (0.08–1.36)	0.42 (0.19–0.96)	0.50 (0.18–1.68)	0.63 (0.08–1.25)	0.42 (0.14–1.36)	1.09 (0.37–1.76)
**Conventional marker [median (range)]**								
CEA	2.50 (0.42–259)	2.35 (0.58–29.9)	3.25 (0.42–259)	1.60 (0.58–6.4)	3.75 (0.61–29.9)	1.85 (0.42–37.9)	5.05 (1.5–259)	1.40 (0.71–2.3)

The additional clinical validation of biomarkers for early‐stage and advanced‐stage CRC, compared to HC individuals, further emphasized the diagnostic potential of specific miRNAs and CEA. In Figure S10, for early‐stage CRC, miR‐23a‐3p, miR‐92a‐3p, miR‐150‐5p and CEA also demonstrated significant differences when compared to HC. For advanced‐stage CRC, miR‐23a‐3p, miR‐92a‐3p, miR‐125a‐3p, miR‐150‐5p and CEA showed significant differences compared to HC. Statistically non‐significant miRNAs and circRNAs in both stages included miR‐19a‐3p, miR‐21‐5p, miR‐1246, circLONP2, circPNN and circLPAR1 (Figures S11 and S12). Further analysis of individual CRC stages revealed significant miRNAs and CEA in stages 0–1, 2, 3 and 4 compared to HC (Figure S13). Among the five markers, miR‐23a‐3p, miR‐92a‐3p and miR‐150‐5p were statistically significant across all stages. However, miR‐125a‐3p and CEA were not significant in stages 0–1 and 3. Statistically non‐significant markers across individual stages included miR‐19a‐3p, miR‐21‐5p, miR‐1246, circLONP2, circPNN and circLPAR1 (Figures S14 and S15). Overall, these results indicate that miR‐23a‐3p, miR‐92a‐3p and miR‐150‐5p can be considered more promising biomarker candidates compared to miR‐125a‐3p and CEA.

To visualize the separation between CRC patients and HC individuals based on combined biomarker profiles, we performed t‐SNE analysis (Figure 5g). The t‐SNE plot shows partial clustering but reveals considerable overlap between CRC and HC samples, suggesting that using these five biomarkers individually may not provide sufficient accuracy. To evaluate the diagnostic performance of each biomarker, we generated ROC curves and calculated the AUC values. Although all biomarkers showed statistical significance, their AUC values reflected inherent limitations as single diagnostic markers. The ROC curves showed AUC values of 0.71 (fair) for miR‐23a‐3p, 0.77 (fair) for miR‐92a‐3p, 0.66 (poor) for miR‐125a‐3p, 0.87 (good) for miR‐150‐5p and 0.75 (fair) for CEA (Figure 5h). Notably, miR‐150‐5p exhibited the highest AUC, though none exceeded 0.9 (excellent classification). Performance metrics, including sensitivity, specificity, accuracy and F1 score, were also calculated based on Youden's index (Figure 5i). Among the biomarkers, miR‐150‐5p demonstrated the highest performance with a sensitivity of 76.25%, specificity of 90%, accuracy of 79% and an F1 score of 85.31%. However, even this marker shows limitations, underscoring the need for combinatorial approaches to enhance diagnostic precision. Nevertheless, the results confirm the effectiveness of the ZAHVIS platform in isolating and analysing EV‐derived NAs, validating the identified miRNAs as potential CRC biomarkers. Although clinical validation establishes a basis for their diagnostic potential, further refinement is required to improve overall diagnostic accuracy.

### 3.6 AI‐Driven Comprehensive Evaluation of Blood Biomarker Combinations for Enhanced CRC Diagnosis

**FIGURE 6:**
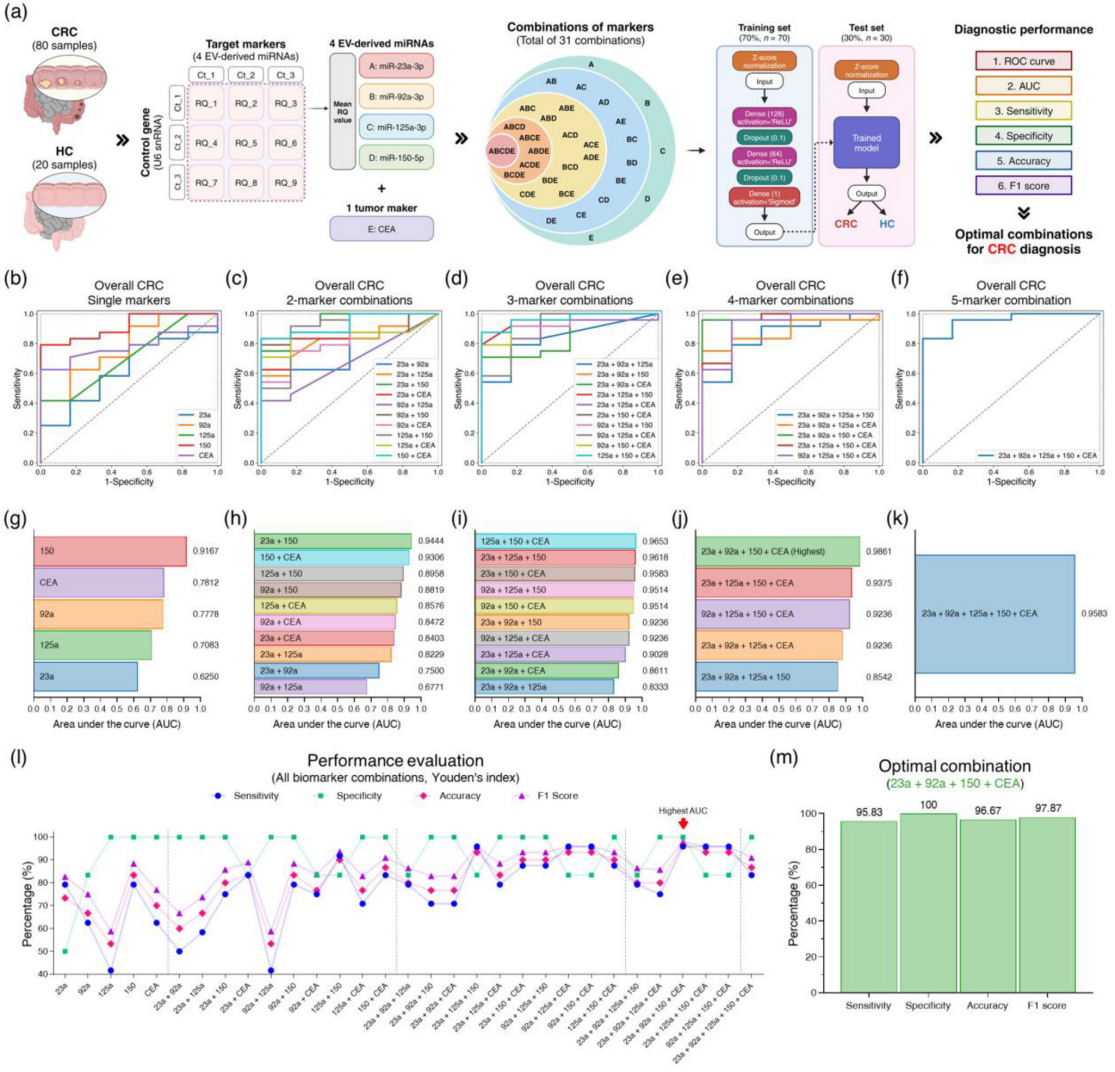
AI‐driven analysis of blood biomarker combinations for CRC in the ZAHV‐AI system. (a) Schematic of the ZAHV‐AI system workflow for evaluating biomarker combinations. EV‐derived RNAs were extracted from 100 blood plasma samples (80 CRC patients and 20 HC individuals) using the ZAHVIS platform. Four miRNAs (miR‐23a‐3p, miR‐92a‐3p, miR‐125a‐3p and miR‐150‐5p) and CEA were quantified. For each miRNA, RQ values were calculated using Ct values from three target markers and control genes, averaged into a single RQ value. These markers were combined into 31 different combinations for AI analysis. The dataset was split into training (70%, *n* = 70) and test (30%, *n* = 30) sets to train a deep learning model to evaluate diagnostic performance. Performance evaluation included metrics such as ROC curves, AUC values, sensitivity, specificity, accuracy and F1 score to determine the most effective biomarker combinations. Created with BioRender.com. (b–f) ROC curves showing diagnostic performance of single markers (b), 2‐marker combinations (c), 3‐marker combinations (d), 4‐marker combinations (e) and a 5‐marker combination (f). (g–k) AUC values for biomarker combinations, ranked by performance, including single markers (g), 2‐marker combinations (h), 3‐marker combinations (i), 4‐marker combinations (j) and a 5‐marker combination (k). (l) Performance evaluation of all biomarker combinations using Youden's index, including sensitivity, specificity, accuracy and F1 score. (m) Bar chart showing performance metrics for the optimal combination (miR‐23a‐3p, miR‐92a‐3p, miR‐150‐5p and CEA). The EV‐derived miRNA markers are labelled simply as 23a, 92a, 125a and 150 in (b–m).

The comprehensive AI‐driven analysis workflow for evaluating blood biomarker combinations for CRC diagnosis using the ZAHV‐AI system is illustrated in Figure 6a. Using EV‐derived RNAs extracted from 100 blood plasma samples (80 CRC patients and 20 HC individuals) through the ZAHVIS platform, four miRNAs (miR‐23a‐3p, miR‐92a‐3p, miR‐125a‐3p and miR‐150‐5p) along with the CEA marker were quantified. For each miRNA, RQ values were calculated from Ct values of three PCR repetitions for the target markers and control gene, averaged over nine RQ values per miRNA. The CEA levels were used directly, leading to 31 different biomarker combinations for AI‐driven analysis. The dataset was divided into training (70%, *n* = 70) and test (30%, *n* = 30) sets using an optimized splitting method (Figure S16 and Table S2). Hyperparameter optimization was performed using *K*‐fold (*K* = 5) cross‐validation. Final model parameters were selected based on the highest combined metric of (1 − average validation loss) and average AUC, resulting in a learning rate of 0.1, batch size of 16, dense layer size of 128 and dropout rate of 0.1. This setup achieved the lowest average validation loss of 0.2383 and a high average AUC of 0.9432 among all tested parameter combinations (Figure S17). The final deep learning model comprised Z‐score normalization, one input layer, two hidden layers (the first with 128 neurons and a dropout rate of 0.1, and the second with 64 neurons and a dropout rate of 0.1), and one output layer. Following model training (Figures S18, S19 and S20), the performance of test set was evaluated using multiple metrics (ROC curve, AUC value, sensitivity, specificity, accuracy and F1 score), and the optimal combinations for CRC diagnosis were identified.

The AI‐driven evaluation provided critical insights across various stages of the disease. In overall CRC, single markers showed varied effectiveness, with miR‐150‐5p achieving the highest AUC of 0.9167. However, combinations significantly improved performance, particularly with miR‐23a‐3p and miR‐150‐5p (AUC: 0.9444), miR‐125a‐3p, miR‐150‐5p and CEA (AUC: 0.9653), and miR‐23a‐3p, miR‐92a‐3p, miR‐150‐5p and CEA (AUC: 0.9861), demonstrating the highest diagnostic accuracy. The combination of miR‐23a‐3p, miR‐92a‐3p, miR‐150‐5p and CEA achieved sensitivity of 95.83%, specificity of 100%, accuracy of 96.67% and an F1 score of 97.87% (Figure 6b–m and Table 2). For subgroup analysis, early‐stage and advanced‐stage samples from the dataset were further analysed, resulting in training (70%, *n* = 42) and test (30%, *n* = 18) sets. In early‐stage and advanced‐stage CRC, the highest single‐marker AUC values were observed with miR‐150‐5p, with values of 0.9028 for early‐stage and 0.9444 for advanced‐stage. For early‐stage CRC, combinations such as miR‐92a‐3p and miR‐150‐5p (AUC: 0.9722) showed improved diagnostic performance, with six combinations achieving the highest AUC of 0.9861 and high‐performance metrics (Figure S21 and Table S3). For advanced‐stage CRC, the combination of miR‐150‐5p and CEA achieved the highest AUC of 0.9583 and high‐performance metrics. (Figure S22 and Table S4). In stage‐specific analysis, individual stage samples from the dataset were further analysed, resulting in training (70%, *n* = 28) and test (30%, *n* = 12) sets. Stage‐specific analysis identified several optimal combinations for each stage of CRC. For stages 0–1, three optimal combinations were identified, all achieving an AUC of 1.0 and perfect performance metrics (Figure S23 and Table S5). For stage 2, four optimal combinations were identified, all achieving an AUC of 0.9722 and high‐performance metrics (Figure S24 and Table S6). For stage 3, the optimal combination of miR‐150‐5p and CEA was identified, achieving an AUC of 1.0 and perfect performance metrics (Figure S25 and Table S7). For stage 4, eleven optimal combinations were identified, all achieving an AUC of 1.0 and perfect performance metrics (Figure S26 and Table S8). These results show the enhanced diagnostic performance achieved through AI‐driven analysis of biomarker combinations. By leveraging multiple biomarkers, the ZAHV‐AI system demonstrated significantly improved sensitivity, specificity, accuracy and F1 scores across various stages of CRC compared to single markers. This approach enhances the diagnostic accuracy for overall CRC and shows remarkable strength in early detection, which is crucial for effective clinical management.

**TABLE 2:** Diagnostic performance of 31 blood biomarker combinations by ZAHV‐AI system for overall CRC.

		Test set (*n* = 30)				
Biomarker combinations^a^ (Total of 31 combinations)		AUC (95% CI)^c^	Sensitivity (%)	Specificity (%)	Accuracy (%)	F1 Score (%)
**Single markers**	23a	0.6250 (0.3791–0.8801)	79.17	50.00	73.33	82.61
	92a	0.7778 (0.5280–0.9808)	62.50	83.33	66.67	75.00
	125a	0.7083 (0.5238–0.8750)	41.67	100	53.33	58.82
	150	0.9167 (0.7913–1.0)	79.17	100	83.33	88.37
	CEA	0.7812 (0.5919–0.9208)	62.50	100	70.00	76.92
**2‐marker combinations**	23a + 92a	0.7500 (0.5381–0.9280)	50.00	100	60.00	66.67
	23a + 125a	0.8229 (0.6488–0.9628)	58.33	100	66.67	73.68
	23a + 150	0.9444 (0.8240–1.0)	75.00	100	80.00	85.71
	23a + CEA	0.8403 (0.6760–0.9680)	83.33	83.33	83.33	88.89
	92a + 125a	0.6771 (0.5026–0.8200)	41.67	100	53.33	58.82
	92a + 150	0.8819 (0.7389–1.0)	79.17	100	83.33	88.37
	92a + CEA	0.8472 (0.6597–1.0)	75.00	83.33	76.67	83.72
	125a + 150	0.8958 (0.6932–1.0)	91.67	83.33	90.00	93.62
	125a + CEA	0.8576 (0.7215–0.9712)	70.83	100	76.67	82.93
	150 + CEA	0.9306 (0.8000–1.0)	83.33	100	86.67	90.91
**3‐marker combinations**	23a + 92a + 125a	0.8333 (0.6561–0.9547)	79.17	83.33	80.00	86.36
	23a + 92a + 150	0.9236 (0.7901–1.0)	70.83	100	76.67	82.93
	23a + 92a + CEA	0.8611 (0.6816–1.0)	70.83	100	76.67	82.93
	23a + 125a + 150	0.9618 (0.8783–1.0)	95.83	83.33	93.33	95.83
	23a + 125a + CEA	0.9028 (0.7677–1.0)	79.17	100	83.33	88.37
	23a + 150 + CEA	0.9583 (0.8718–1.0)	87.50	100	90.00	93.33
	92a + 125a + 150	0.9514 (0.8634–1.0)	87.50	100	90.00	93.33
	92a + 125a + CEA	0.9236 (0.7438–1.0)	95.83	83.33	93.33	95.83
	92a + 150 + CEA	0.9514 (0.8459–1.0)	95.83	83.33	93.33	95.83
	125a + 150 + CEA	0.9653 (0.8976–1.0)	87.50	100	90.00	93.33
**4‐marker combinations**	23a + 92a + 125a + 150	0.8542 (0.6630–0.9843)	79.17	83.33	80.00	86.36
	23a + 92a + 125a + CEA	0.8819 (0.7497–0.9887)	75.00	100	80.00	85.71
	23a + 92a + 150 + CEA^b^	0.9861 (0.9360–1.0)	95.83	100	96.67	97.87
	23a + 125a + 150 + CEA	0.9375 (0.7917–1.0)	95.83	83.33	93.33	95.83
	92a + 125a + 150 + CEA	0.9236 (0.7452–1.0)	95.83	83.33	93.33	95.83
**5‐marker combinations**	23a + 92a + 125a + 150 + CEA	0.9583 (0.8653–1.0)	83.33	100	86.67	90.91

^a^ The EV‐derived miRNAs are labelled simply as 23a, 92a, 125a and 150.

^b^ Optimal combination.

^c^ 95% CI, 95% confidence interval.

### 3.7 Diagnostic Performance of Optimal Blood Biomarker Combinations for Early Detection of CRC in the ZAHV‐AI System

**FIGURE 7:**
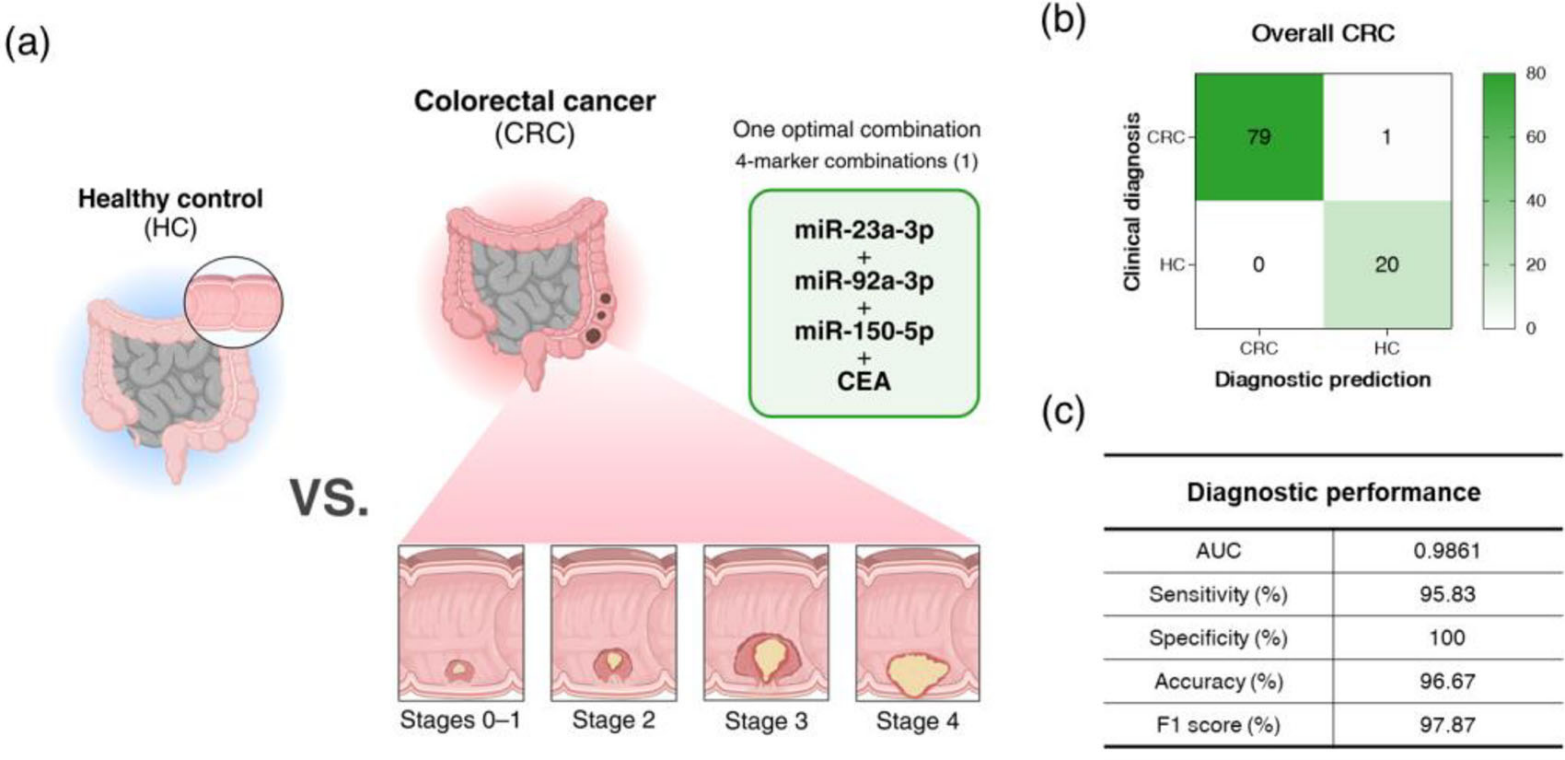
Optimal biomarker combination and diagnostic performance for overall CRC in the ZAHV‐AI system. (a) Schematic overview of the optimal blood biomarker combination for overall CRC, comparing HC and CRC groups. Created with BioRender.com. (b) Confusion matrix of diagnostic predictions. (c) Diagnostic performance metrics (AUC, sensitivity, specificity, accuracy, F1 score).

The ZAHV‐AI system demonstrates remarkable diagnostic capabilities for CRC through optimal blood biomarker combinations. For overall CRC detection, the system identified an optimal biomarker combination consisting of miR‐23a‐3p + miR‐92a‐3p + miR‐150‐5p + CEA, as illustrated in the schematic overview (Figure 7a). Using this combination, the system achieved outstanding diagnostic performance (AUC 0.9861, sensitivity 95.83%, specificity 100%, accuracy 96.67%, F1 score 97.87%) as summarized in the confusion matrix and performance metrics (Figure 7b,c). Subgroup analyses of early‐stage, advanced‐stage and individual CRC stages, including their optimal biomarker combinations and diagnostic performance metrics, are summarized in Figure S27. In early‐stage CRC, six combinations were identified, including miR‐23a‐3p + miR‐150‐5p + CEA; miR‐92a‐3p + miR‐125a‐3p + miR‐150‐5p; miR‐92a‐3p + miR‐150‐5p + CEA; miR‐125a‐3p + miR‐150‐5p + CEA; miR‐23a‐3p + miR‐92a‐3p + miR‐150‐5p + CEA; and miR‐23a‐3p + miR‐125a‐3p + miR‐150‐5p + CEA. These combinations yielded an AUC of 0.9861, sensitivity of 91.67%, specificity of 100%, accuracy of 94.44% and F1 score of 95.65%. For advanced‐stage CRC, the combination of miR‐150‐5p + CEA achieved an AUC of 0.9583, sensitivity of 91.67%, specificity of 100%, accuracy of 94.44% and F1 score of 95.65%, highlighting the system's effectiveness in detecting both early and advanced stages of the disease. For stages 0–1, perfect diagnostic performance (AUC 1.0, sensitivity 100%, specificity 100%, accuracy 100%, F1 score 100%) was achieved with three combinations, namely, miR‐150‐5p; miR‐92a‐3p + miR‐150‐5p; and miR‐92a‐3p + miR‐150‐5p + CEA. Stage 2 exhibited high performance with four combinations, including miR‐23a‐3p + miR‐92a‐3p; miR‐92a‐3p + miR‐150‐5p; miR‐125a‐3p + miR‐150‐5p; and miR‐92a‐3p + miR‐125a‐3p + CEA, achieving an AUC of 0.9722, sensitivity of 83.33%, specificity of 100%, accuracy of 91.67% and F1 score of 90.91%. For stage 3, perfect diagnostic metrics were observed with the combination of miR‐150‐5p + CEA. Stage 4 also demonstrated perfect diagnostic performance with eleven combinations, including miR‐150‐5p; miR‐23a‐3p + miR‐150‐5p; miR‐125a‐3p + CEA; miR‐150‐5p + CEA; miR‐23a‐3p + miR‐125a‐3p + miR‐150‐5p; miR‐92a‐3p + miR‐150‐5p + CEA; miR‐125a‐3p + miR‐150‐5p + CEA; miR‐23a‐3p + miR‐92a‐3p + miR‐125a‐3p + miR‐150‐5p; miR‐23a‐3p + miR‐125a‐3p + miR‐150‐5p + CEA; miR‐92a‐3p + miR‐125a‐3p + miR‐150‐5p + CEA; and miR‐23a‐3p + miR‐92a‐3p + miR‐125a‐3p + miR‐150‐5p + CEA. These comprehensive analyses the ZAHV‐AI system effectively identifies optimal blood biomarker combinations, improving diagnostic accuracy for CRC, particularly at early stages. By integrating efficient EV isolation through the ZAHVIS platform with AI‐driven analysis, the ZAHV‐AI system offers a streamlined, highly sensitive and accurate approach to CRC diagnostics. Overall, the ZAHV‐AI system provides a useful tool for ongoing monitoring and personalized treatment, demonstrating its potential for clinical application and contributing to improvements in cancer diagnostics and patient care.

## 4 Discussion

The ZAHV‐AI system, which combines the ZAHVIS platform for EV isolation with AI‐driven biomarker analysis, shows significant promise for enhancing CRC diagnostics. Traditional EV isolation methods, such as UC and commercial precipitation kits like the TEI, are often limited by their time‐consuming, labour‐intensive processes and heightened contamination risks. Even more recent techniques, such as SEC, while faster and more straightforward, face challenges with reproducibility and detection sensitivity, as they require collecting multiple fractions. This disperses EVs across large volumes, making SEC more suited for purification than enrichment, which limits its sensitivity in achieving accurate diagnostics. In contrast, the ZAHVIS platform utilizes the unique properties of ZA and MDH crosslinkers to offer a more efficient and advanced solution compared to simpler electrostatic methods, such as cation‐charged polymer filters (Chen et al. 2010; Yasui et al. 2017). Although simpler methods are easier to produce, they often suffer from non‐specific binding and contamination, reducing EV purity. The ZAHVIS platform, however, enhances traditional electrostatic interactions by incorporating covalent bonding, leveraging both mechanisms in a single‐step process. This combination provides stronger and more selective binding forces, offering significant advantages in the enrichment and separation of EVs and EV‐derived molecules during downstream analysis. The high surface area and porous structure of ZA enhance EV capture, while MDH crosslinkers ensure stable, contamination‐free isolation, leading to more concentrated and purified samples critical for accurate diagnostics. Furthermore, compared to advanced ultrafiltration systems such as the exosome detection method via the ultrafast‐isolation system (EXODUS), which are known for their high speed and purity in EV isolation, the system still faces potential concerns regarding EV clogging and the need for specialized equipment, which can limit its scalability in clinical applications (Chen et al. 2021). In contrast, the ZAHVIS platform effectively overcomes these limitations by capturing EVs on the ZA surface, which prevents EV clogging and ensures reliable performance without the need for complex equipment. This make the ZAHVIS platform more scalable and suitable for high‐throughput clinical settings where efficiency and ease of use are crucial. A key advantage of the ZAHVIS platform is its ability to streamline the entire EV isolation process. Traditional methods typically require separate steps for isolating EVs and extracting EV‐derived biomolecules, extending processing time. The ZAHVIS platform simplifies this by isolating EVs and extracting proteins or NAs in a single step, thereby shortening the overall process and preserving the integrity of EV‐derived biomolecules. As summarized in Table 3, the ZAHVIS platform offers several technical benefits. With 10 min for EV enrichment, the platform completes EV isolation in a total of 15 min, EV‐derived protein extraction in 30 min and EV‐derived NA extraction in 35 min. Additionally, the ZAHVIS platform is highly cost‐effective, priced at less than $2 per sample, making it accessible for use in various laboratory and clinical settings. Its flexibility in handling different sample volumes supports scalability, making it a practical choice for EV‐based diagnostics. The AI‐driven analysis component of the ZAHV‐AI system further enhances its diagnostic capabilities by overcoming the limitations associated with single biomarkers. Building on previously established methodologies that applied machine learning to cancer diagnostics (Kim et al. 2021; Liu et al. 2021), we further refine these approaches by incorporating an optimized data‐splitting method along with *K*‐fold cross‐validation to reduce overfitting and enhance model reliability. By leveraging advanced deep learning algorithms, the ZAHV‐AI system evaluates combinations of biomarkers validated through cell lines and clinical studies, identifying and proposing optimal combinations for improved CRC diagnosis. This integration of the ZAHVIS platform and AI‐driven analysis offers a comprehensive, efficient and highly sensitive diagnostic approach. The streamlined workflow, coupled with the high sensitivity and specificity afforded by AI‐driven blood biomarker combination analysis, represents a significant advancement in CRC diagnostics, particularly for early detection. This system has the potential to provide earlier and more accurate diagnoses, which is critical for improving patient outcomes in CRC.

**TABLE 3:** Comparison of ZAHVIS platform and traditional methods for EV isolation.

Methods	ZAHVIS platform	Ultracentrifugation (UC)	Precipitation	Size exclusion chromatography (SEC)
**Underlying principle**	Electrostatic and covalent binding with ZA and MDH	High‐speed centrifugal force	Polymer‐induced precipitation	Size‐based separation
**Procedure steps**	1. ZA and MDH mix for EV capture 2. Filtration for EV enrichment 3. Washing and EV elution	1. Centrifugation at 300 g 2. Centrifugation at 2000 g 3. Centrifugation at 10,000 g 4. Centrifugation at 11,000 g 5. EV elution by resuspending pellet	1. Precipitation reagents mix 2. Overnight incubation 3. Centrifugation at 10,000 g 4. EV elution by resuspending pellet	1. Column setup and equilibration 2. Column flushing 3. Sample loading 4. EV elution
**Required materials**	ZA and MDH	None	Precipitation reagent^a^	Specific SEC column^b^
**Required devices**	None	Ultracentrifuge	Centrifuge	Auto fraction collector (AFC)
**Temperature**	Roop temperature (RT)	4°C and RT	4°C and RT	RT
**Sample volume capacity**	Flexible (depending on syringe/filter size)	> 10 mL	< 20 mL	Flexible (depending on column size)
**Isolation time**	< 15 min	3–4 h	> 12 h	1–2 h (Manually) < 20 min (AFC)
**EV yield**	Moderate to High	Moderate to High	High	Low to Moderate
**EV purity**	Moderate to High	High	Low	High
**Scalability**	High	Moderate	Moderate	Moderate
**Laboratory accessibility**	High	Low	High	Moderate
**Cost per isolation**	$2 (ZA: $0.005, MDH: $0.15, Syringe filter: $1.5, Additional supplier: $0.3)	$15	$12	$50
**Advantages**	Simple procedure Fast Low cost User‐friendly	Most common method No reagents used	Simple procedure Low cost	Simple procedure (AFC) Fast
**Disadvantages**	Needs more validation	Bulky equipment required Time‐consuming Labor‐intensive Potential EV damage	Non‐EV fraction contamination Polymer contamination	Complex procedure (Manually) High cost Multiple fraction collection Limited EV enrichment
**Molecular extraction**	EV enrichment and extraction (One‐step)		1. EV isolation 2. Molecular extraction (Two‐step)	
**Extraction time**	< 35 min		Several hours	
**Protein** **extraction**	1. EV enrichment (10 min) 2. RIPA lysis buffer (< 20 min)		1. EV isolation (1–4 h or > 12 h) 2. RIPA lysis buffer (40 min)	
**Nucleic acid** **extraction**	1. EV enrichment (10 min) 2. NP‐40 lysis buffer (20 min) 3. Washing and elution (< 5 min)		1. EV isolation (1–4 h or > 12 h) 2. Commercial kit^c^ (2 h)	

^a^ Total Exosome Isolation Reagent (4478359, Invitrogen).

^b^ qEV Isolation Columns (IC10‐70, Izon Science).

^c^ Total Exosome RNA & Protein Isolation Kit (4478545, Invitrogen).

Blood‐based testing using conventional CEA markers has demonstrated significant limitations in the early diagnosis of CRC, often failing to detect early‐stage cancers due to its lower sensitivity and specificity. In our study, CEA as a single marker showed low diagnostic performance for early diagnosis, with AUC values of 0.7361 (fair) for early‐stage CRC and 0.6944 (poor) for stages 0–1 and stage 2, indicating its insufficient accuracy. Although CEA is an established diagnostic marker for CRC, its effectiveness is significantly reduced in early‐stage detection. For advanced‐stage CRC, however, CEA demonstrated better performance, with AUC values of 0.8333 (good) for advanced stages, 0.9722 (excellent) for stage 3 and stage 4. These results underscore the limitations of CEA when used as a single marker for early‐stage CRC detection. Screening and early diagnosis of CRC remain challenging with new liquid‐based markers due to their low detection rates and lower sensitivity in early‐stage CRC compared to advanced stages. For example, a multi‐analyte blood test showed a median sensitivity of 43% for stage 1 cancers, compared to 73% and 78% for stages 2 and 3 (Cohen et al. 2018). Another study found circulating tumour DNA alterations in 50% of stage 1 CRC patients, while the detection rates were much higher in stages 2, 3 and 4 (89%, 90% and 93%, respectively) (Phallen et al. 2017). These findings suggest that early‐stage CRC tends to have smaller tumour sizes, which release minimal quantities of DNA into the bloodstream, making early detection more challenging. Nevertheless, ZAHV‐AI system provides reliable early detection by combining multiple EV‐derived miRNA markers with CEA, thus enhancing diagnostic performance. For early‐stage CRC, six optimal combinations were identified, with an AUC of 0.9861. Notably, CEA was included in five of these six combinations. For stages 0–1, the combination of miR‐92a‐3p + miR‐150‐5p + CEA achieved perfect diagnostic performance with an AUC of 1.0. Additionally, the combination of miR‐92a‐3p + miR‐125a‐3p + CEA for stage 2 was identified as optimal, with an AUC of 0.9722. By combining CEA with other EV‐derived blood biomarkers, the ZAHV‐AI system effectively enhances the diagnostic accuracy for early‐stage CRC, thereby addressing the inherent limitations of CEA as a diagnostic marker and offering a more robust tool for early detection.

The ZAHV‐AI system shows remarkable potential for the early detection of CRC by identifying optimal biomarker combinations using EVs derived from blood plasma. Using traditional statistical methods in our study, miR‐23a‐3p, miR‐92a‐3p and miR‐150‐5p emerged as significant diagnostic markers. However, even though some individual markers like miR‐125a‐3p and CEA did not show significance in certain stages, the evaluation of biomarker combinations by the ZAHV‐AI system highlighted the importance of using a panel of markers for enhanced diagnostic performance. For instance, in early‐stage CRC, combinations such as miR‐125a‐3p + miR‐150‐5p + CEA and miR‐23a‐3p + miR‐125a‐3p + miR‐150‐5p + CEA demonstrated robust diagnostic capability. Additionally, in stages 0–1, the combination of miR‐92a‐3p + miR‐150‐5p + CEA was most effective, while for stage 2, miR‐92a‐3p + miR‐125a‐3p + CEA provided the highest diagnostic accuracy. These findings suggest that traditional methods of assessing diagnostic markers based solely on individual statistical significance may be limiting. The AI‐driven analysis of the ZAHV‐AI system reveals that a combinatorial approach, leveraging multiple markers, can significantly enhance diagnostic performance. This indicates that potential biomarkers might be overlooked if we rely only on their individual statistical significance. The ZAHV‐AI system's AI‐driven approach highlights that markers previously considered less useful can meaningfully contribute to cancer diagnosis when evaluated as part of a marker panel or in combination with other markers. These findings suggest that a combinatorial approach, leveraging multiple markers, can provide superior diagnostic performance, especially in early‐stage CRC detection.

In our study, we identified optimal blood biomarker combinations for CRC, including one for overall CRC, six for early‐stage CRC, one for advanced‐stage CRC, three for stages 0–1, four for stage 2, one for stage 3 and eleven for stage 4. Despite applying these optimal combinations, the ZAHV‐AI system failed to correctly classify one sample each in the overall CRC, early‐stage, advanced‐stage and stage 2 analyses (Figures 7b and S28). These results suggest that while the ZAHV‐AI system shows promising diagnostic performance, further refinement is needed to improve accuracy. In particular, the relatively small control group and subdivision of CRC cases may limit the statistical power of subgroup analyses, contributing to variability in classification outcomes. Expanding the cohort to include a larger and more diverse population, especially with more early‐stage cases, will be essential to strengthen the system's robustness and validate biomarker performance more reliably. Additionally, the integration of a simple deep learning algorithm with real‐time PCR enables quick training and easy implementation without requiring specialized computational expertise. However, this simplicity may limit the ability to capture complex interactions between biomarkers, potentially affecting diagnostic accuracy. Moreover, clinical miRNA diagnostics often face challenges due to variability introduced during EV isolation, which can impact biomarker detection and reproducibility. Although the ZAHVIS platform demonstrated consistent detection in this study, further validation is needed to address factors such as batch‐to‐batch consistency and sample handling conditions. Standardizing these parameters will be essential to minimize variability and facilitate clinical translation. Future studies could also explore advanced EV detection systems, such as magneto‐electrochemical devices (Park et al. 2021) or dual‐surface‐protein orthogonal barcoding (Lei et al. 2023), in combination with the ZAHVIS platform to further enhance diagnostic sensitivity and specificity. Addressing these limitations and expanding the study could establish the ZAHV‐AI system as a practical tool for CRC staging, treatment monitoring and prognosis. Furthermore, the ZAHV‐AI system is versatile and applicable to other biological samples, such as urine, saliva and oral swab, as well as various cancer types, including prostate, breast and lung cancer. Future studies will further enhance the capabilities of the ZAHV‐AI system, broadening its impact on cancer diagnostics and improving patient outcomes.

## Author Contributions

**Bonhan Koo**: conceptualization(equal), formal analysis(equal), investigation(equal), methodology(equal), writing–original draft(equal). **Young Il Kim**: formal analysis(equal), investigation(equal), methodology(equal), writing–original draft(equal). **Minju Lee**: formal analysis(equal), investigation(equal), methodology(equal). **Seok‐Byung Lim**: conceptualization(equal), supervision(equal), writing–original draft(equal), writing–review and editing(equal). **Yong Shin**: conceptualization (equal), funding acquisition (equal), project administration (equal), supervision (equal), writing–original draft (equal), writing–review and editing (equal).

## Consent

Informed consent was obtained from all patients.

## Conflicts of Interest

The authors declare no conflicts of interest.

## Supporting information



Supporting information

## Data Availability

The source code for the AI‐driven analysis and the data used in this study are available at: https://github.com/BKoo‐Codes/ZAHV‐AI‐system_CRC‐Early‐diagnostics.
