# Supplementary material for: Enhanced Early Detection of Colorectal Cancer via Blood Biomarker Combinations Identified Through Extracellular Vesicle Isolation and Artificial Intelligence Analysis
Source: J Extracell Vesicles. 2025 Jun 13;14(6):e70088. doi: 10.1002/jev2.70088 (PMC12163753; doi:10.1002/jev2.70088)
Supplement: Supplementary file 1 — Supporting information [file JEV2-14-e70088-s001.docx]

Supporting Information

**Enhanced early detection of colorectal cancer via optimal blood biomarker combinations identified through efficient extracellular vesicle isolation and artificial intelligence analysis**

Bonhan Koo^1#^, Young Il Kim^2#^, Minju Lee^1^, Seok-Byung Lim^2*^, Yong Shin^1*^

^1^Department of Biotechnology, College of Life Science and Biotechnology, Yonsei University, Republic of Korea

^2^Division of Colon and Rectal Surgery, Department of Surgery, Asan Medical Center, University of Ulsan College of Medicine, Republic of Korea

^#^Bonhan Koo and Young Il Kim contributed equally to this study.

*e-mail: shinyongno1@yonsei.ac.kr, sblim@amc.seoul.kr

Supplementary Figures: S1 – S28

Supplementary Tables: S1 – S8


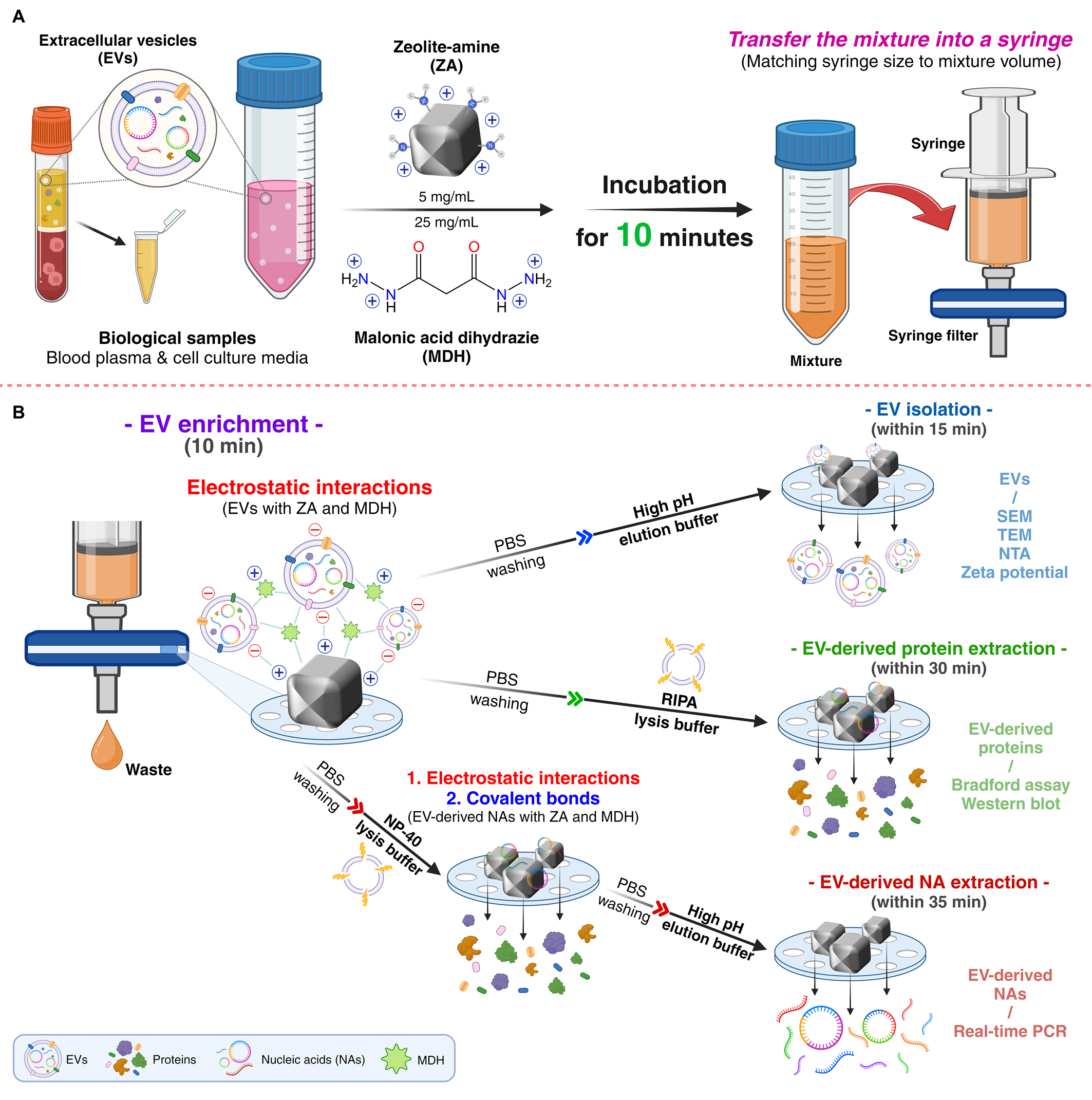


**Figure S1.** Schematic of the ZAHVIS platform workflow. (A) Initial steps in the ZAHVIS platform for EV enrichment. Biological samples (blood plasma and cell culture media) are mixed with zeolite-amine (ZA) and malonic acid dihydrazide (MDH) at 5 mg mL^−1^ and 25 mg mL^−1^, respectively. After 10 minutes of incubation to allow EVs to attach to the ZA surface, the mixture is transferred into a syringe equipped with a filter. Created with BioRender.com. (B) Detailed ZAHVIS workflow. EV enrichment occurs via electrostatic interactions between EVs and the combination of ZA and MDH during the 10-minute incubation. Enriched EVs are processed for isolation, protein extraction, and NA extraction. EV isolation (within 15 minutes) involves PBS washing and elution with a high pH buffer, followed by analysis using scanning electron microscopy (SEM), transmission electron microscopy (TEM), nanoparticle tracking analysis (NTA), and zeta potential measurements. EV-derived protein extraction (within 30 minutes) involves PBS washing and lysis with RIPA lysis buffer, analyzed by Bradford assay and western blot. EV-derived NA extraction (within 35 minutes) includes PBS washing, NP-40 lysis, and binding to ZA and MDH via electrostatic and covalent interactions. NAs are eluted with a high pH buffer and analyzed by real-time PCR. Created with BioRender.com.


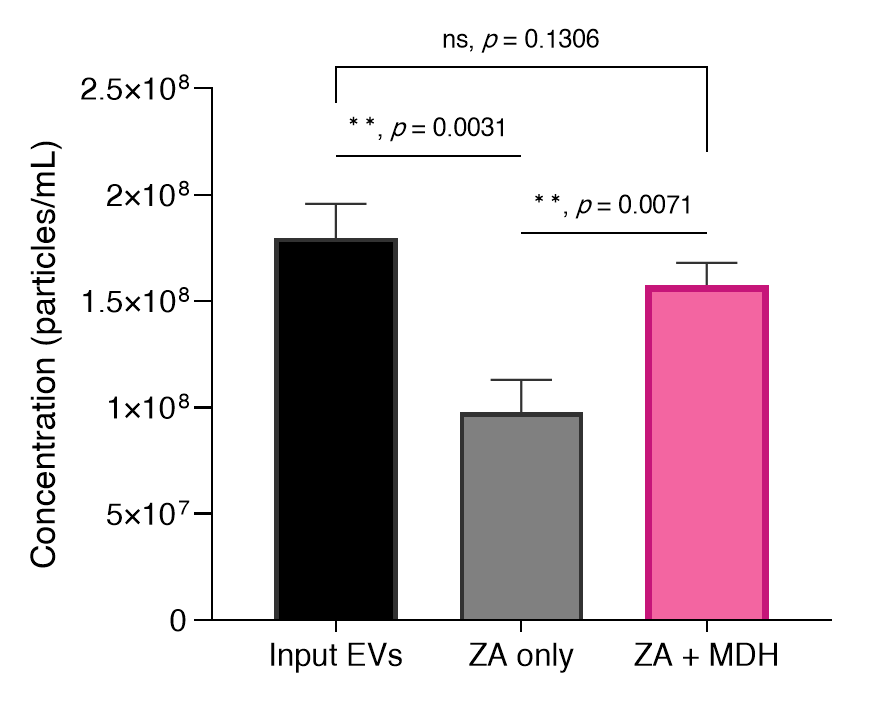


**Figure S2.** Comparative analysis of EV isolation efficiency with and without covalent bonding. Input EVs represent UC-isolated EVs without further isolation, ZA only shows isolation based on electrostatic interaction, and ZA + MDH represents isolation using the dual mechanism combining electrostatic interaction and covalent bonding. Bars represent mean ± standard deviation (s.d.). Statistical significance indicated by **P* < 0.05, ***P* < 0.01, ****P* < 0.001, and *****P* < 0.0001.


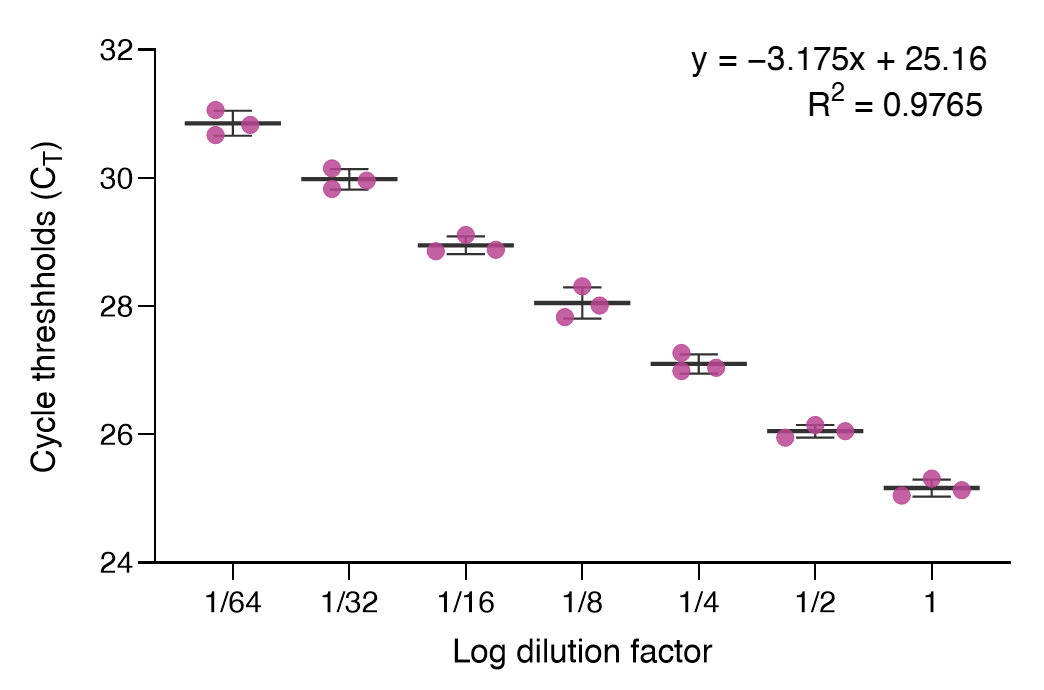


**Figure S3.** Evaluation of the reproducibility of the ZAHVIS platform in biomarker detection. Plasma samples were serially diluted by half up to 1/64, and EV-derived miRNA was extracted using the one-step ZAHVIS method. miR-21-5p levels were quantified by real-time PCR. Dot plots represent individual samples, with a line at the mean ± s.d.


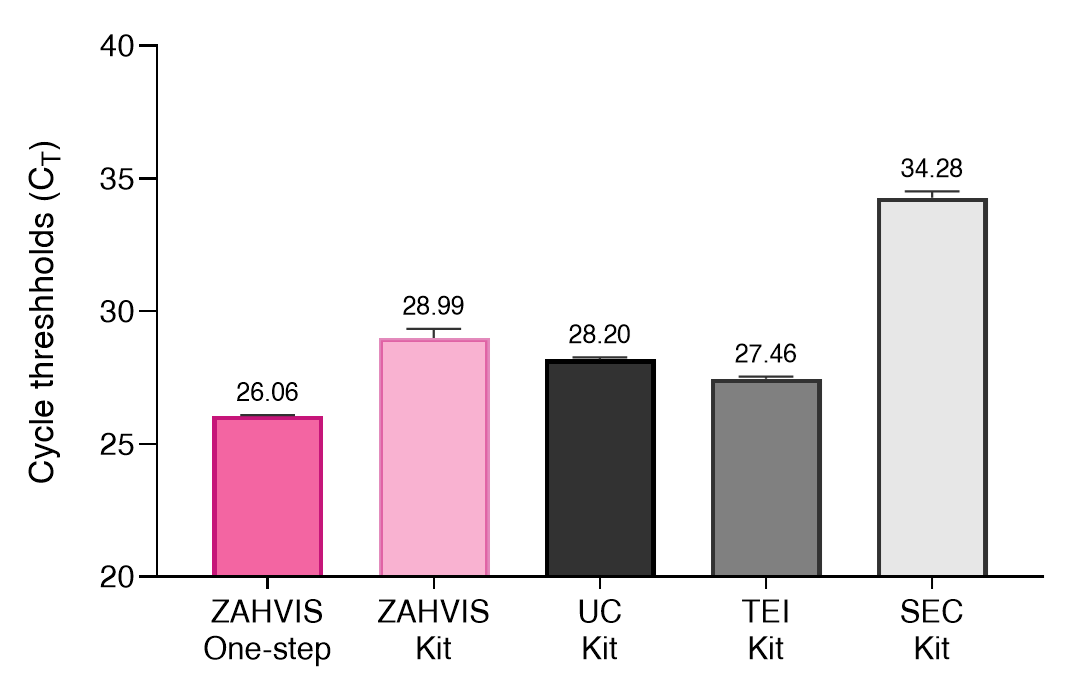


**Figure S4.** Comparison of EV-derived RNA recovery across different EV isolation methods. The one-step ZAHVIS approach, which integrates EV enrichment and RNA extraction in a single workflow, was compared with conventional workflows where EVs were first isolated using ZAHVIS, ultracentrifugation (UC), total exosome isolation (TEI), or size exclusion chromatography (SEC), followed by RNA extraction using a commercial kit (Total Exosome RNA & Protein Isolation Kit). miR-21-5p levels were quantified by real-time PCR. Bars represent mean ± s.d.


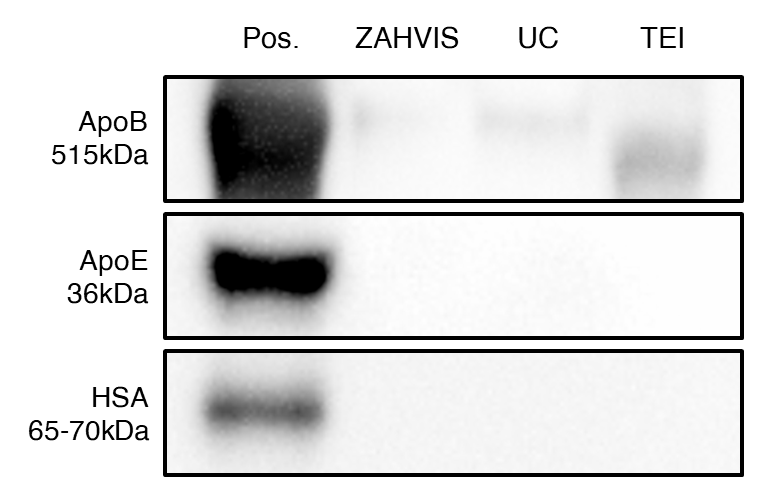


**Figure S5.** Comparative analysis of plasma-derived protein removal efficiency. Western blot analysis of Apolipoprotein B (ApoB), Apolipoprotein E (ApoE), and human serum albumin (HSA) in EV samples isolated from human plasma using the ZAHVIS platform, UC, and TEI methods. The positive control (Pos.) represents original human plasma prior to isolation.


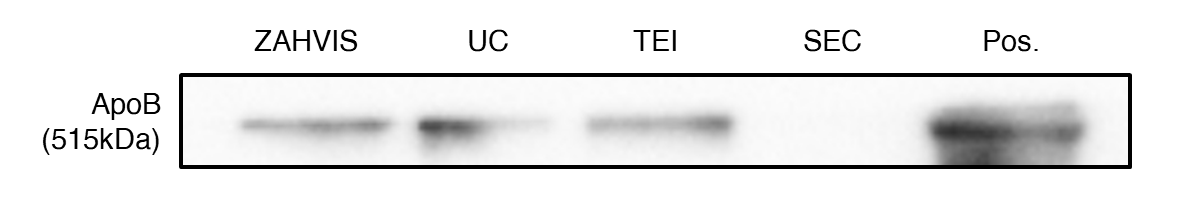


**Figure S6.** Comparative assessment of lipoprotein-associated protein removal efficiency. Western blot analysis of ApoB in EV samples isolated using the ZAHVIS platform, UC, TEI, and SEC methods. UC-isolated EV fractions were spiked with ApoB at a concentration of 16 µg mL^−1^ prior to isolation. The positive control (Pos.) represents ApoB from human plasma prior to isolation.


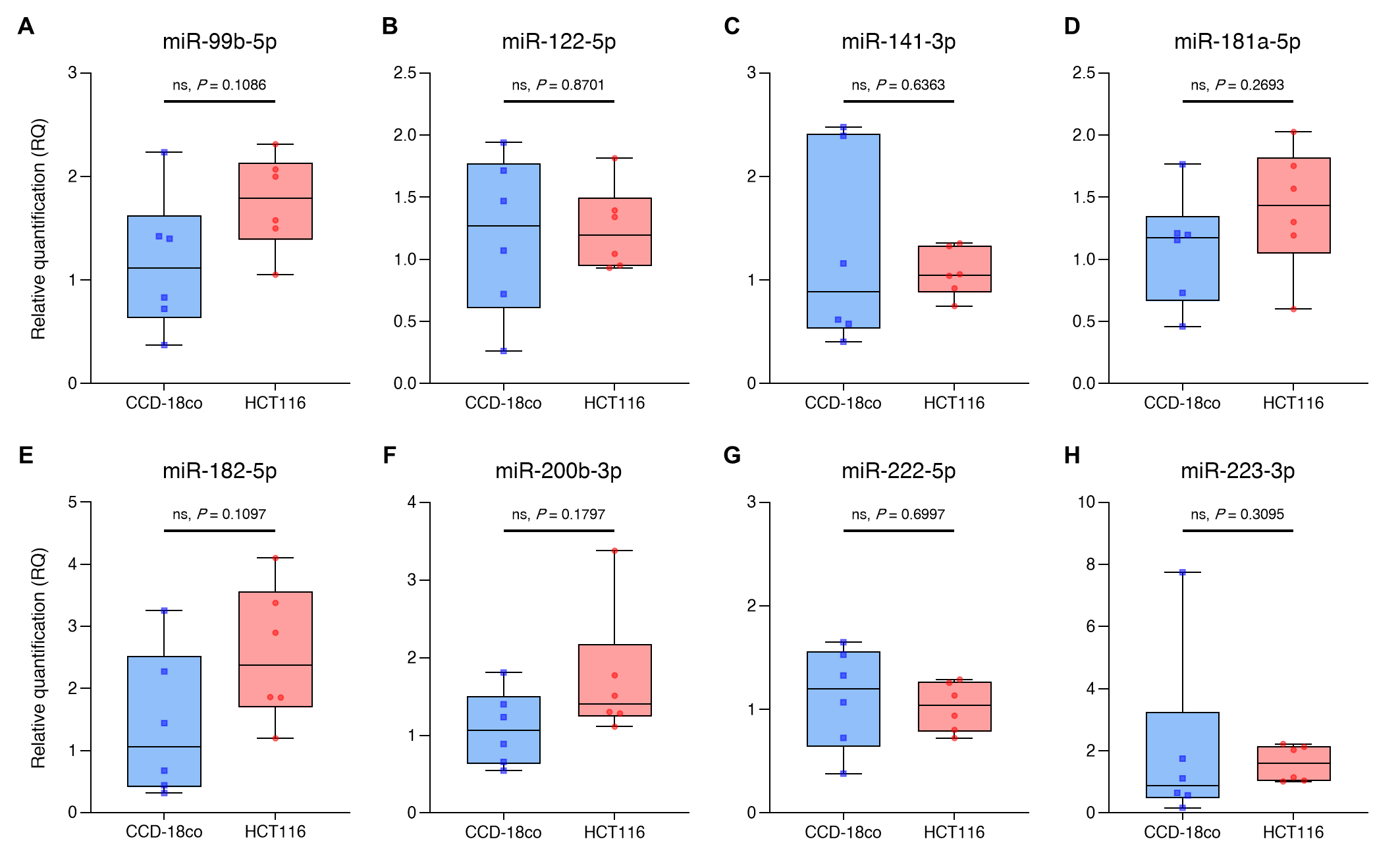


**Figure S7.** Analysis of candidate biomarkers in EV-derived RNA from cell line models using the ZAHVIS platform. (A–H) Box plots showing the relative quantification (RQ) values of selected non-significant biomarker candidates. EV-derived RNAs were extracted from the cell culture media of HCT116 (cancer cells) and CCD-18co (normal cells) using the ZAHVIS platform. Among the 15 miRNAs and 3 circRNAs analyzed, miR-99b-5p (A), miR-122-5p (B), miR-141-3p (C), miR-181a-5p (D), miR-182-5p (E), miR-200b-3p (F), miR-222-5p (G), and miR-223-3p (H) showed no statistical significance (*P* > 0.05). *n* = 6 biologically independent experiments per group. Box plots represent the median, interquartile range (IQR), and whiskers indicating the minimum and maximum values. Each dot represents an individual sample.


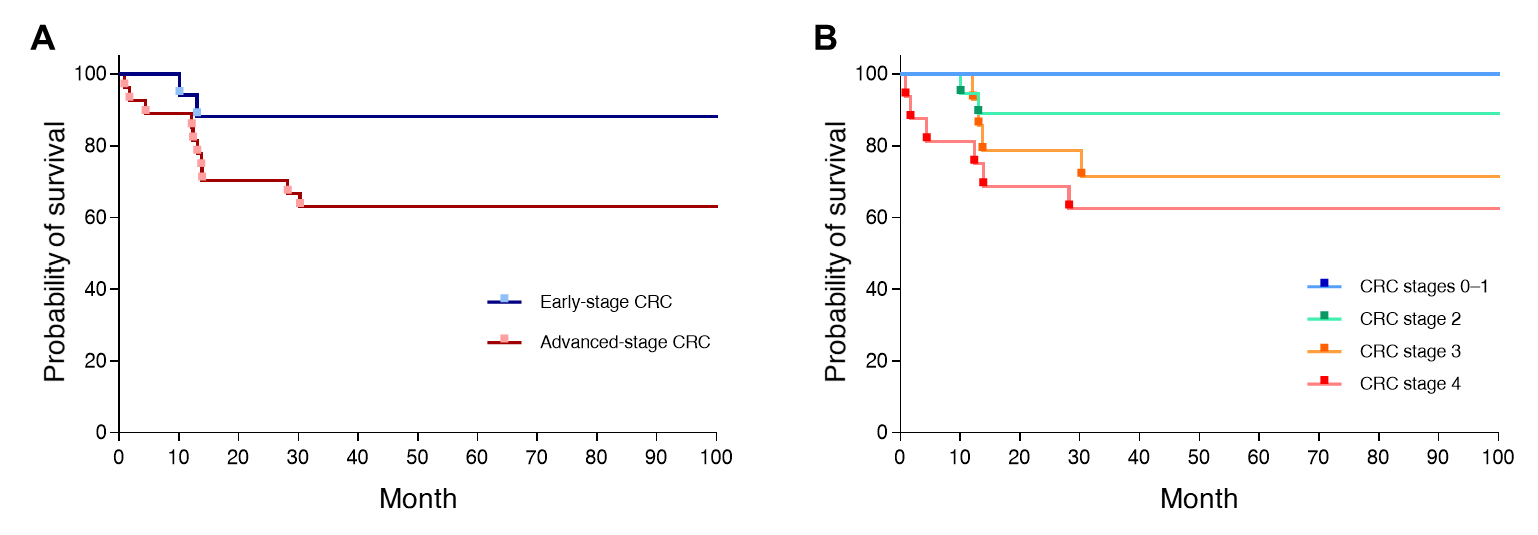


**Figure S8.** CRC stage-specific cumulative disease-free survival probabilities. Kaplan-Meier survival curves showing cumulative disease-free survival probabilities for CRC patients over 100 months, stratified by stage. (A) Early-stage CRC (stages 0–2, *n* = 40) and advanced-stage CRC (stages 3–4, *n* = 40). (B) Detailed breakdown by CRC stages 0–1, stage 2, stage 3, and stage 4 (*n* = 20 for each stage).


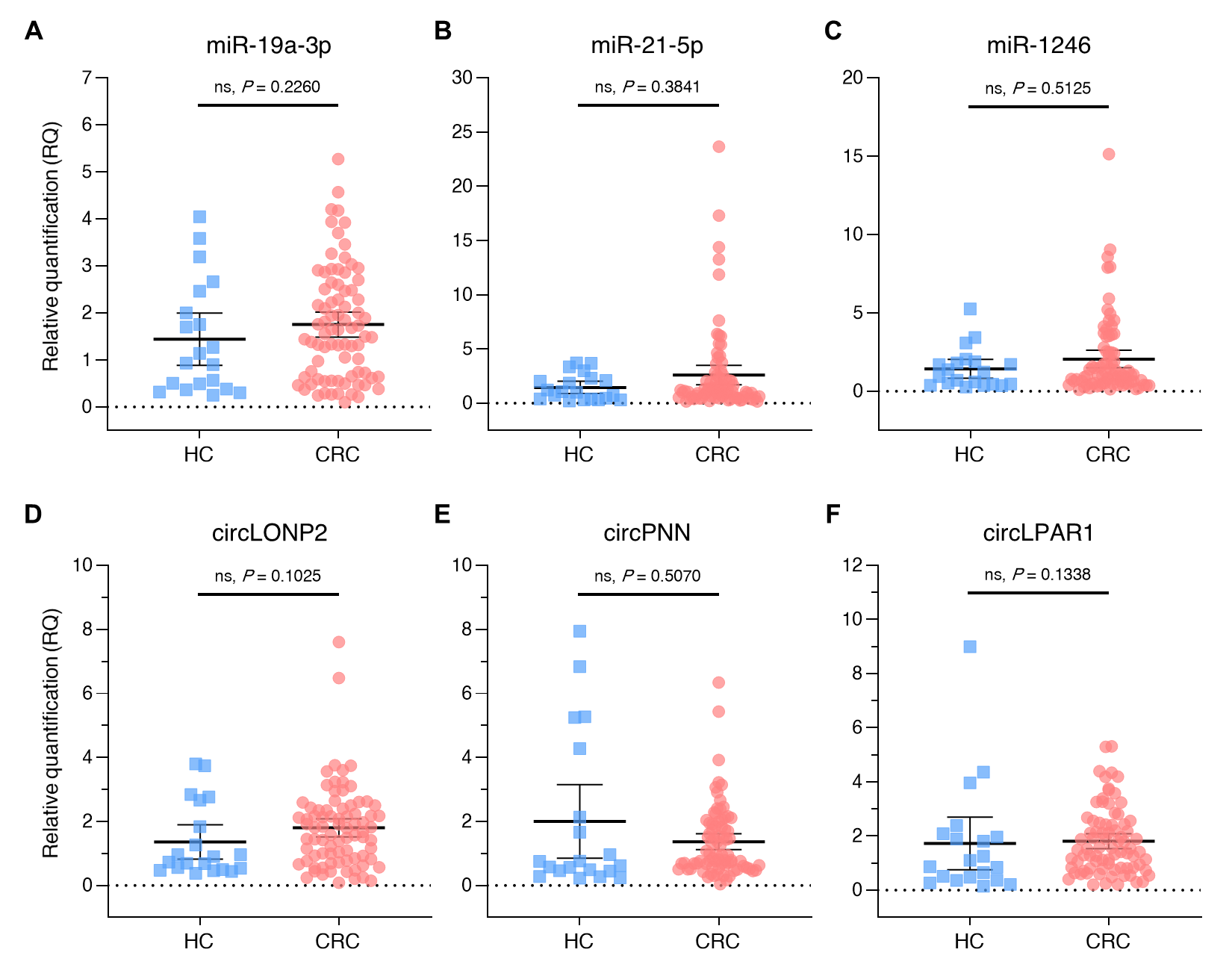


**Figure S9.** Analysis of candidate biomarkers in EV-derived RNA from clinical samples using the ZAHVIS platform. (A–F) Dot plots showing the RQ values of biomarkers identified in cell line models but not statistically significant in clinical samples. EV-derived RNAs were extracted from the blood plasma of CRC patients (*n* = 80) and HC individuals (*n* = 20) using the ZAHVIS platform. Among the 7 miRNAs and 3 circRNAs selected from cell line models, miR-19a-3p (A), miR-21-5p (B), and miR-1246 (C) for miRNAs, and circLONP2 (D), circPNN (E), and circLPAR1 (F) for circRNAs showed no statistical significance (ns, *P* > 0.05). Dot plots represent individual samples, with a line at the median and IQR.


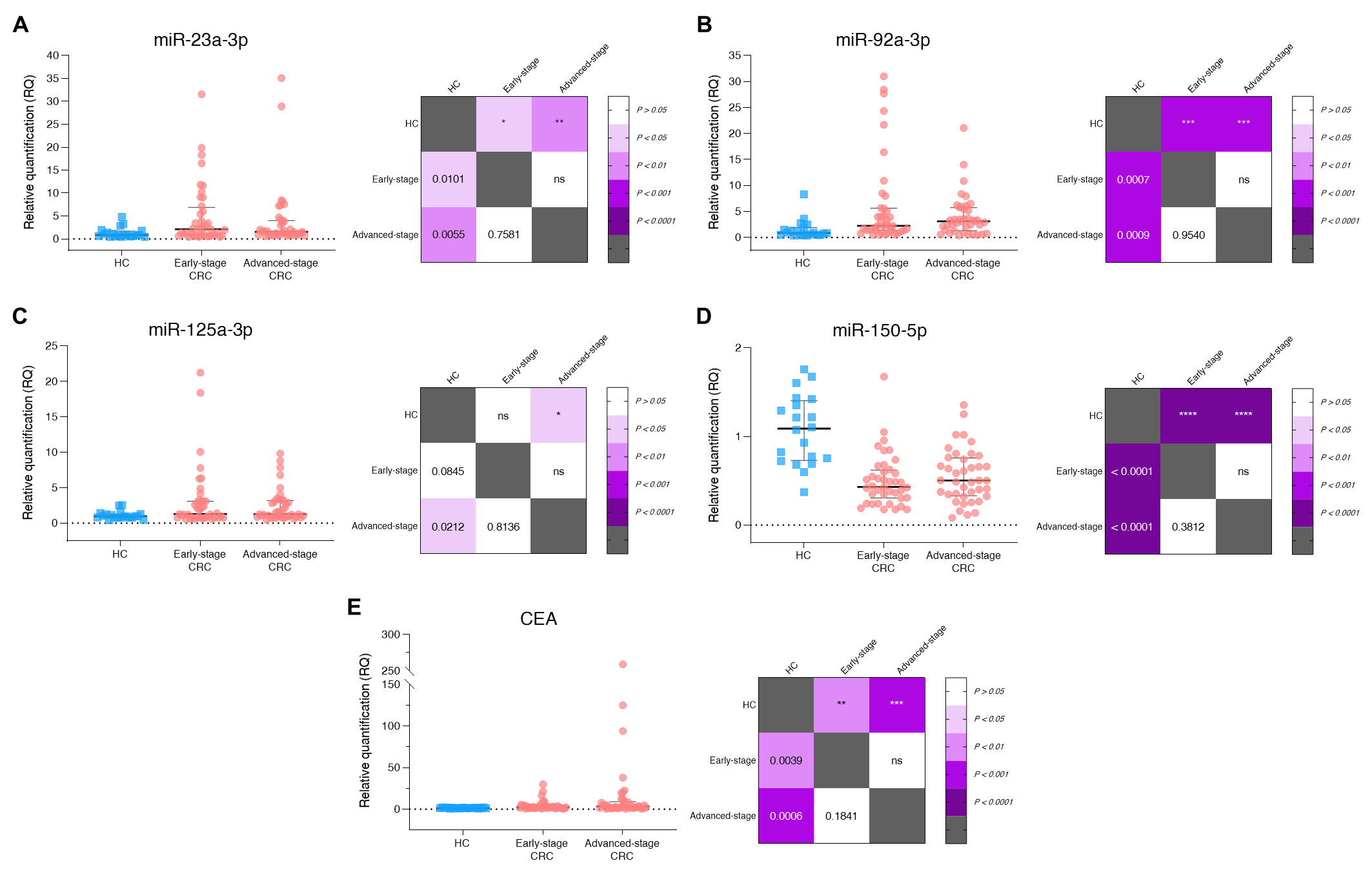


**Figure S10.** Clinical validation of statistically significant EV-derived miRNA and CEA biomarkers in early-stage and advanced-stage CRC. (A–E) Dot plots and heatmaps showing the RQ values of clinically validated EV-derived miRNA and CEA in CRC, categorized into early-stage (stages 0–2, *n* = 40) and advanced-stage (stages 3–4, *n* = 40), compared to HC (*n* = 20). The analyzed miRNAs include miR-23a-3p (A), miR-92a-3p (B), miR-125a-3p (C), and miR-150-5p (D), with CEA levels shown in (E). Heatmaps indicate statistical significance between groups (ns, *P* > 0.05; **P* < 0.05; ***P* < 0.01; ****P* < 0.001; *****P* < 0.0001). Dot plots represent individual samples, with a line at the median and IQR.


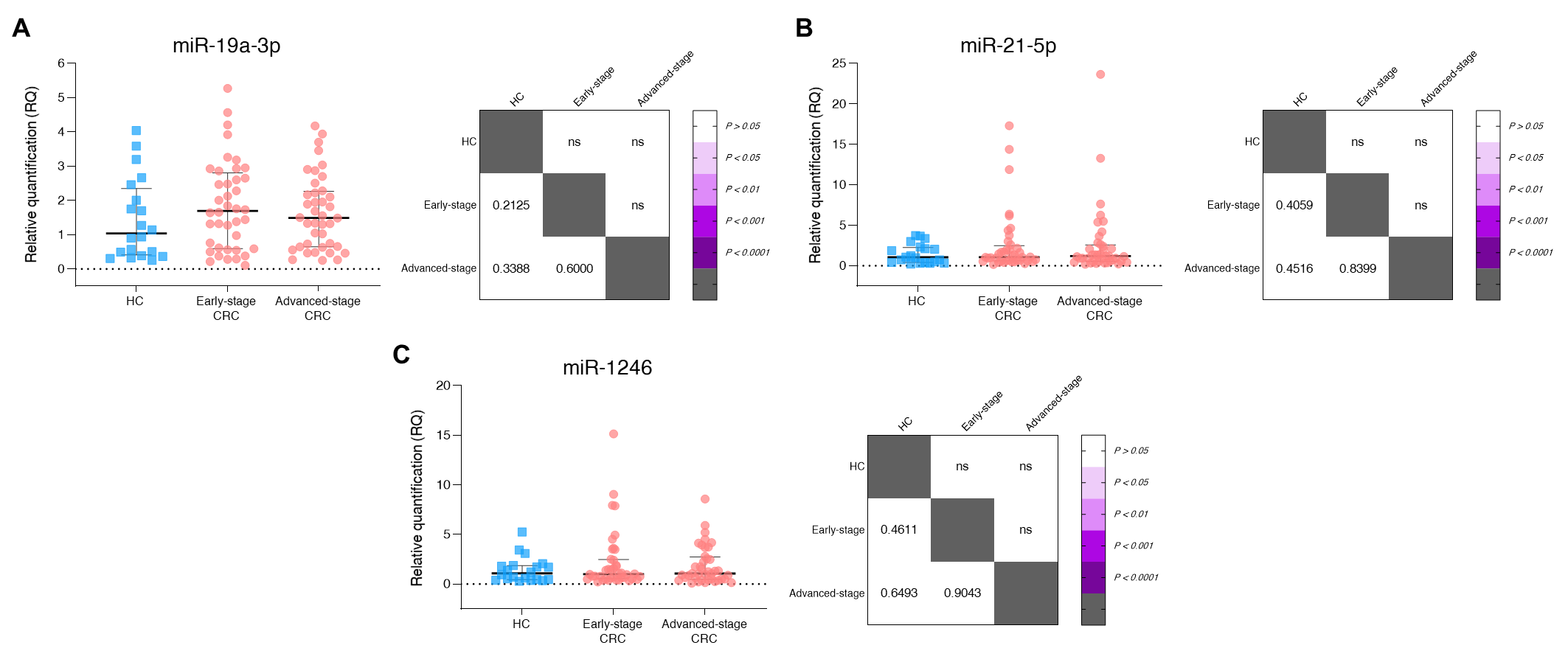


**Figure S11.** EV-derived miRNA biomarkers in early-stage and advanced-stage CRC. (A–C) Dot plots and heatmaps showing the RQ values of statistically non-significant EV-derived miRNAs in CRC, categorized by early-stage (*n* = 40) and advanced-stage (*n* = 40), compared to HC (*n* = 20). The analyzed miRNAs include miR-19a-3p (A), miR-21-5p (B), and miR-1246 (C). Heatmaps indicate statistical significance between groups (ns, *P* > 0.05; **P* < 0.05; ***P* < 0.01; ****P* < 0.001; *****P* < 0.0001). Dot plots represent individual samples, with a line at the median and IQR.


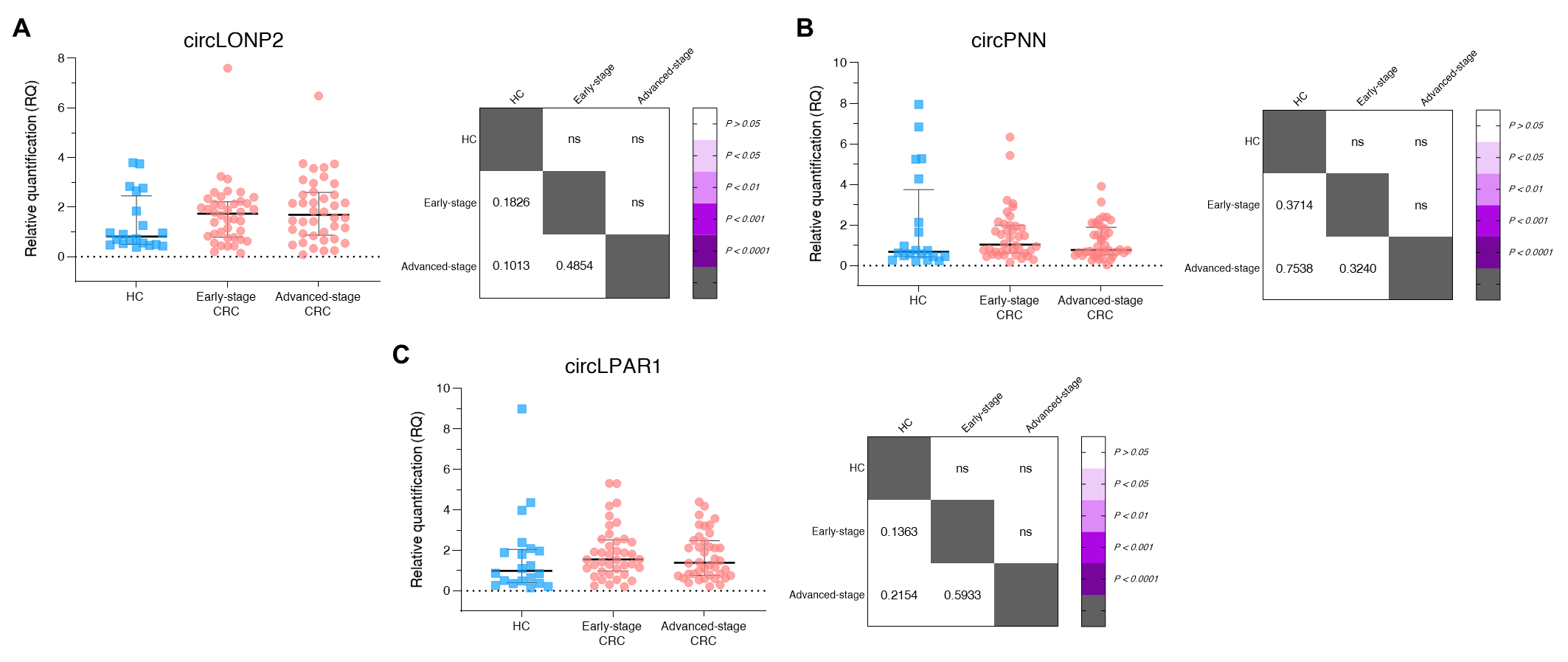


**Figure S12.** EV-derived circRNA biomarkers in early-stage and advanced-stage CRC. (A–C) Dot plots and heatmaps showing the RQ values of statistically non-significant EV-derived circRNAs in CRC, categorized by early-stage (*n* = 40) and advanced-stage (*n* = 40), compared to HC (*n* = 20). The analyzed circRNAs include circLONP2 (A), circPNN (B), and circLPAR1 (C). Heatmaps indicate statistical significance between groups (ns, *P* > 0.05; **P* < 0.05; ***P* < 0.01; ****P* < 0.001; *****P* < 0.0001). Dot plots represent individual samples, with a line at the median and IQR.


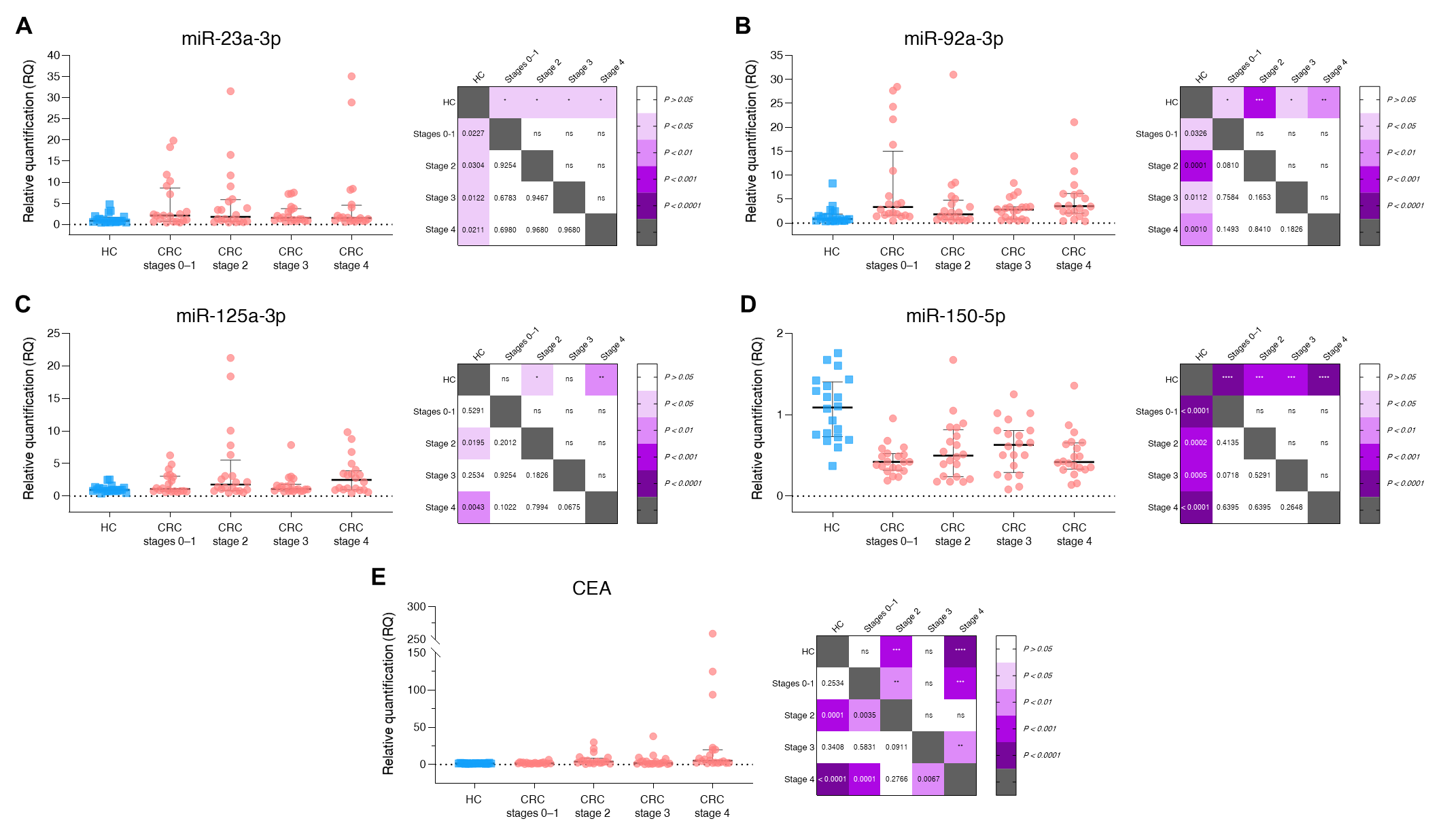


**Figure S13.** Clinical validation of statistically significant EV-derived miRNA and CEA biomarkers in individual CRC stages. (A–E) Dot plots and heatmaps showing the RQ values of clinically validated EV-derived miRNAs and CEA in CRC, categorized by individual stages (stages 0–1, stage 2, stage 3, and stage 4; *n* = 20 for each stage), compared to HC (*n* = 20). The analyzed miRNAs include miR-23a-3p (A), miR-92a-3p (B), miR-125a-3p (C), and miR-150-5p (D), with CEA levels shown in (E). Heatmaps indicate statistical significance between groups (ns, *P* > 0.05; **P* < 0.05; ***P* < 0.01; ****P* < 0.001; *****P* < 0.0001). Dot plots represent individual samples, with a line at the median and IQR.


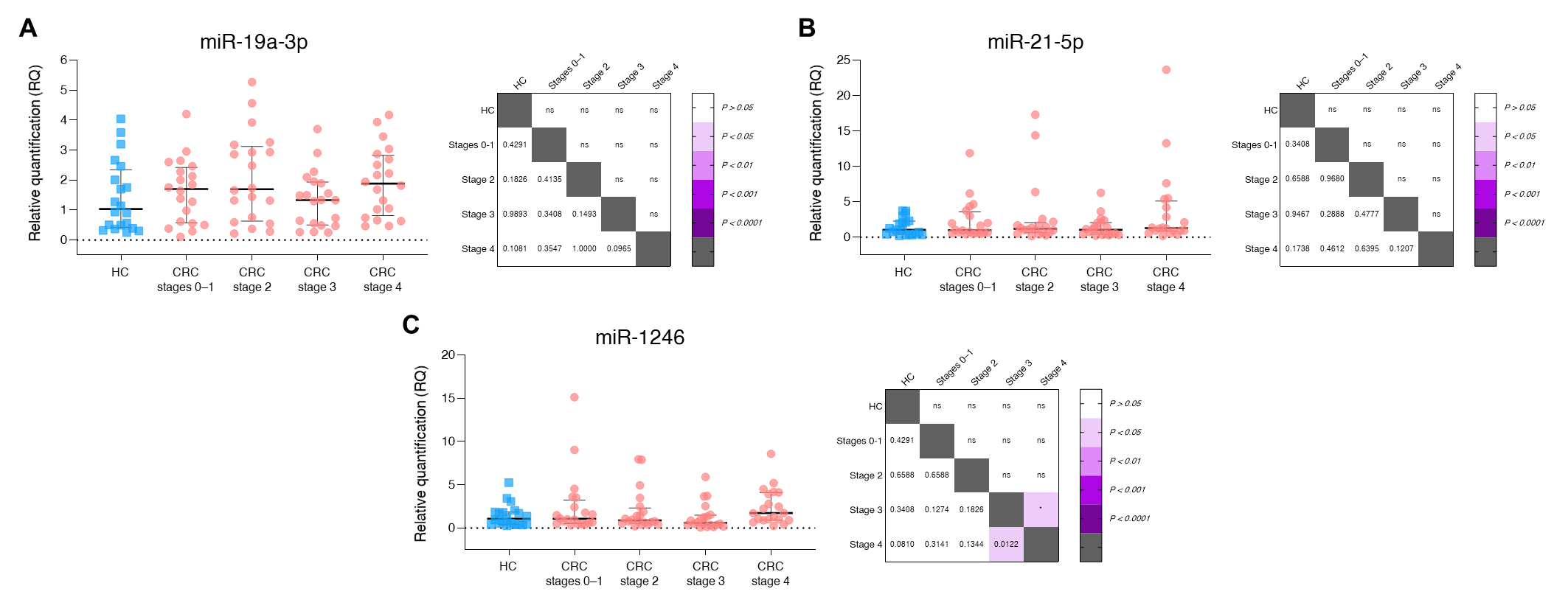


**Figure S14.** EV-derived miRNA biomarkers in individual CRC stages. (A–C) Dot plots and heatmaps showing the RQ values of statistically non-significant EV-derived miRNAs in CRC, categorized by individual stages (stages 0–1, stage 2, stage 3, and stage 4; *n* = 20 for each stage), compared to HC (*n* = 20). The analyzed miRNAs include miR-19a-3p (A), miR-21-5p (B), and miR-1246 (C). Heatmaps indicate statistical significance between groups (ns, *P* > 0.05; **P* < 0.05; ***P* < 0.01; ****P* < 0.001; *****P* < 0.0001). Dot plots represent individual samples, with a line at the median and IQR.


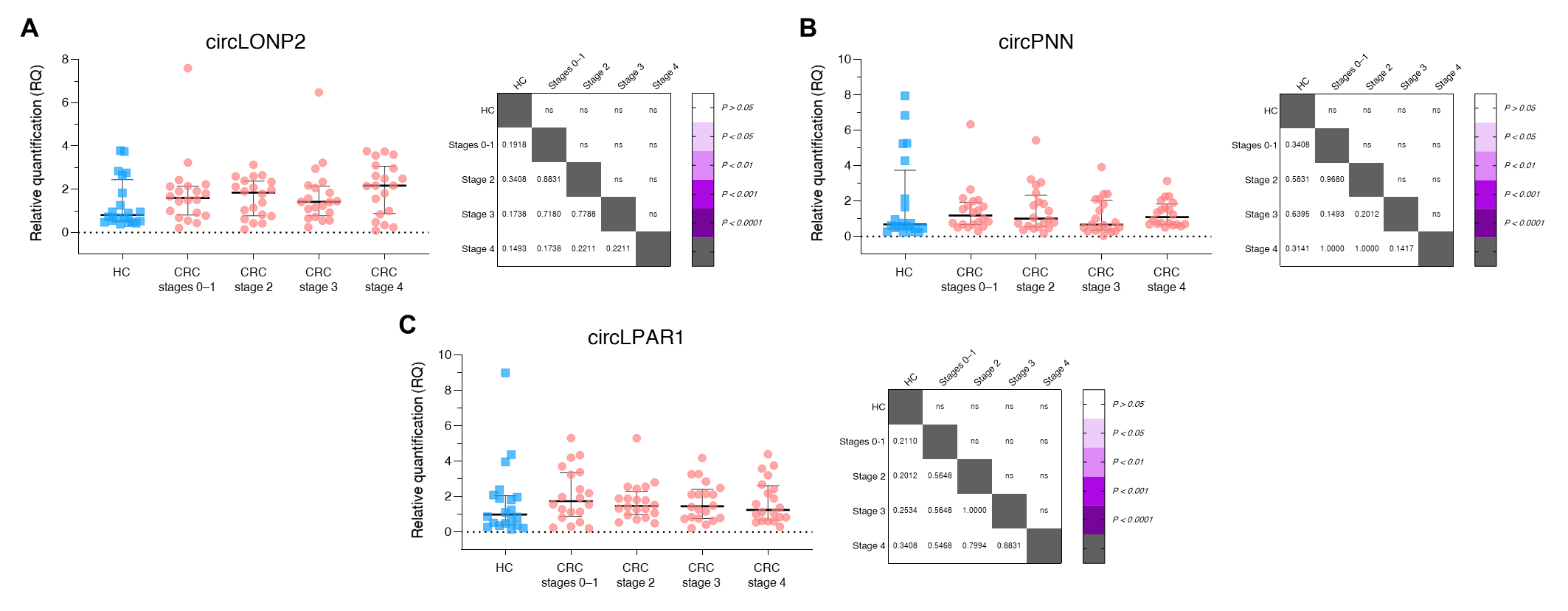


**Figure S15.** EV-derived circRNA biomarkers in individual CRC stages. (A–C) Dot plots and heatmaps showing the RQ values of statistically non-significant circRNAs in CRC, categorized by individual stages (stages 0–1, stage 2, stage 3, and stage 4; *n* = 20 for each stage), compared to HC (*n* = 20). The analyzed circRNAs include circLONP2 (A), circPNN (B), and circLPAR1 (C). Heatmaps indicate statistical significance between groups (ns, *P* > 0.05; **P* < 0.05; ***P* < 0.01; ****P* < 0.001; *****P* < 0.0001). Dot plots represent individual samples, with a line at the median and IQR.


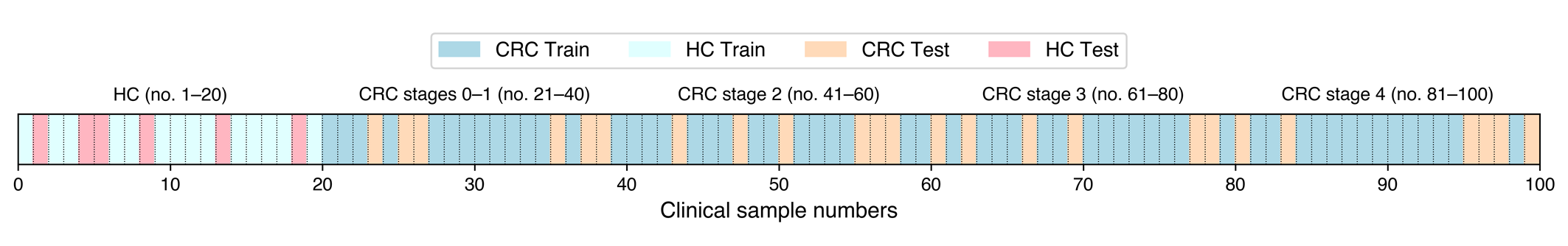


**Figure S16.** Optimized splitting of clinical samples into training and test sets. Clinical samples were divided into training (70%, *n* = 70) and test (30%, *n* = 30) sets using an optimized splitting method. This method ensures representative class distribution for HC and CRC stages 0–1, stage 2, stage 3, and stage 4. The optimal split was determined by minimizing the mean squared error of feature means and standard deviations between training and test sets.


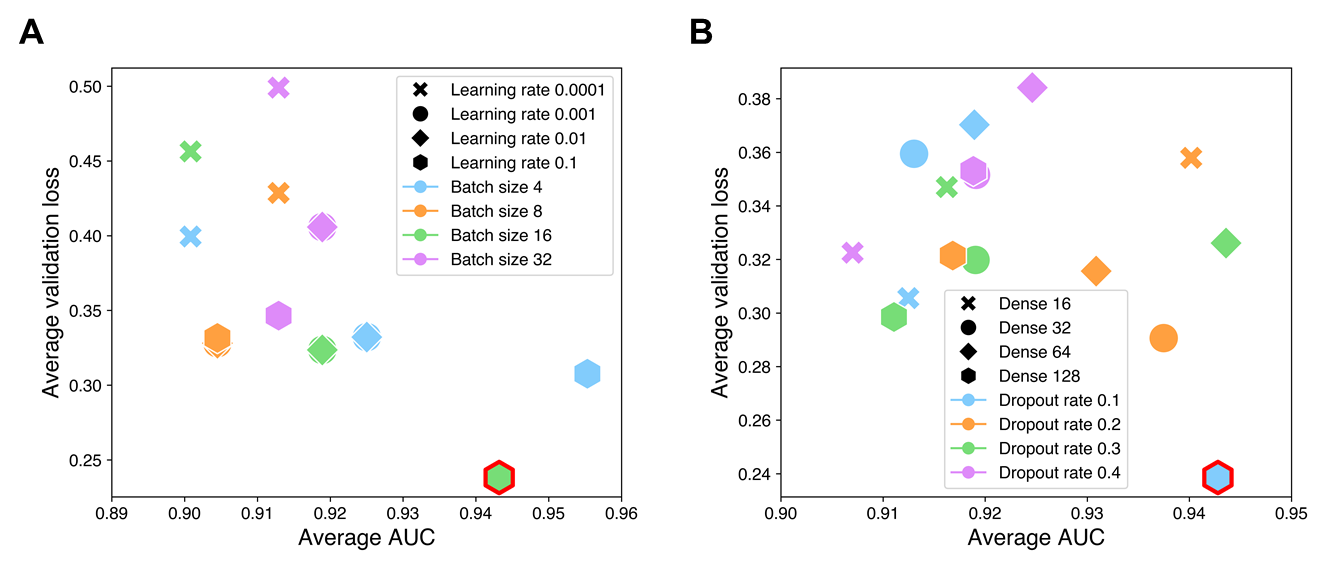


**Figure S17.** Hyperparameter optimization for model training. Dot plots showing the average validation loss and average AUC for 256 different hyperparameter combinations in deep learning-based analysis using K-fold cross-validation (K = 5). (A) Hyperparameter combinations of learning rates (0.0001, 0.001, 0.01, 0.1) and batch sizes (4, 8, 16, 32) with a dense layer size of 128 and a dropout rate of 0.1. (B) Hyperparameter combinations of dense layer sizes (16, 32, 64, 128) and dropout rates (0.1, 0.2, 0.3, 0.4) with a learning rate of 0.1 and a batch size of 16. The final parameters (red outline), which included a learning rate of 0.1, batch size of 16, dense layer size of 128, and dropout rate of 0.1, were selected for the highest combined metric of (1 − average validation loss) and average AUC.


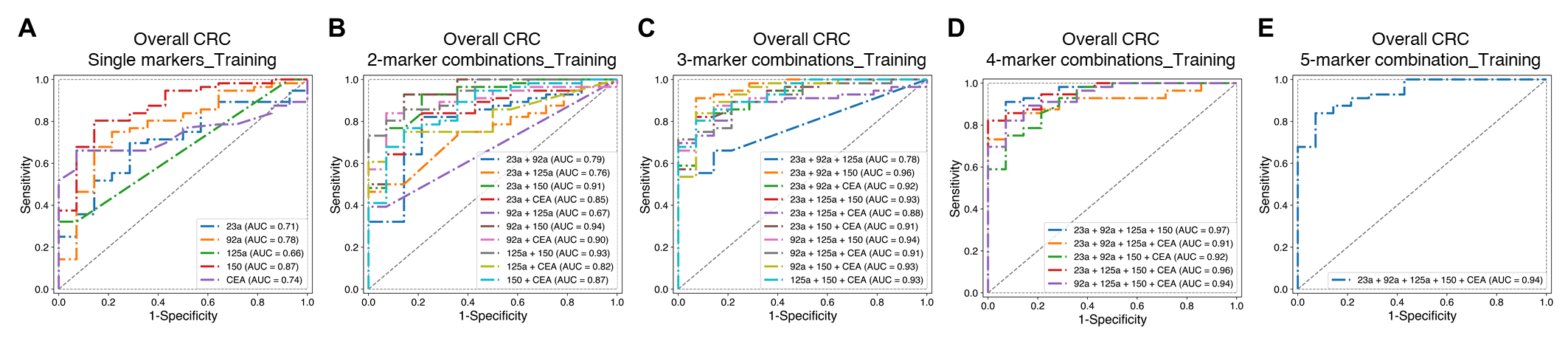


**Figure S18.** ROC curves and AUC values of the training set for blood biomarker combinations in overall CRC. (A–E) ROC curves showing the diagnostic performance of single markers (A), 2-marker combinations (B), 3-marker combinations (C), 4-marker combinations (D), and a 5-marker combination (E) in the training set for overall CRC diagnosis. Each plot includes the AUC values for the corresponding biomarker combinations, evaluated using the deep learning model. The EV-derived miRNA markers are labeled simply as 23a, 92a, 125a, and 150 in (A–E).


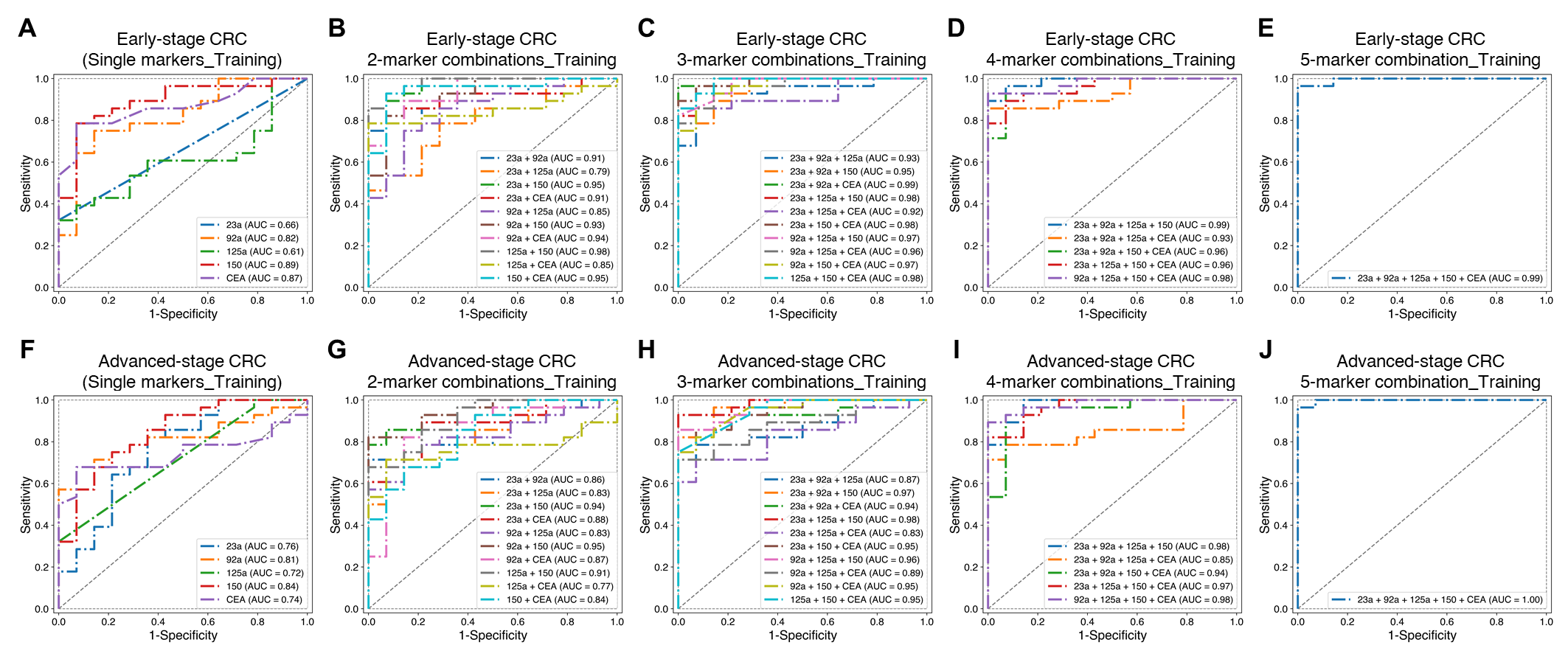


**Figure S19.** ROC curves and AUC values of the training set for blood biomarker combinations in early-stage and advanced-stage CRC. (A–E) ROC curves showing the diagnostic performance of single markers (A), 2-marker combinations (B), 3-marker combinations (C), 4-marker combinations (D), and a 5-marker combination (E) in the training set for early-stage CRC diagnosis. (F–J) ROC curves showing the diagnostic performance of single markers (F), 2-marker combinations (G), 3-marker combinations (H), 4-marker combinations (I), and a 5-marker combination (J) in the training set for advanced-stage CRC diagnosis. Each plot includes the AUC values for the corresponding biomarker combinations, evaluated using the deep learning model. The EV-derived miRNA markers are labeled simply as 23a, 92a, 125a, and 150 in (A–J).


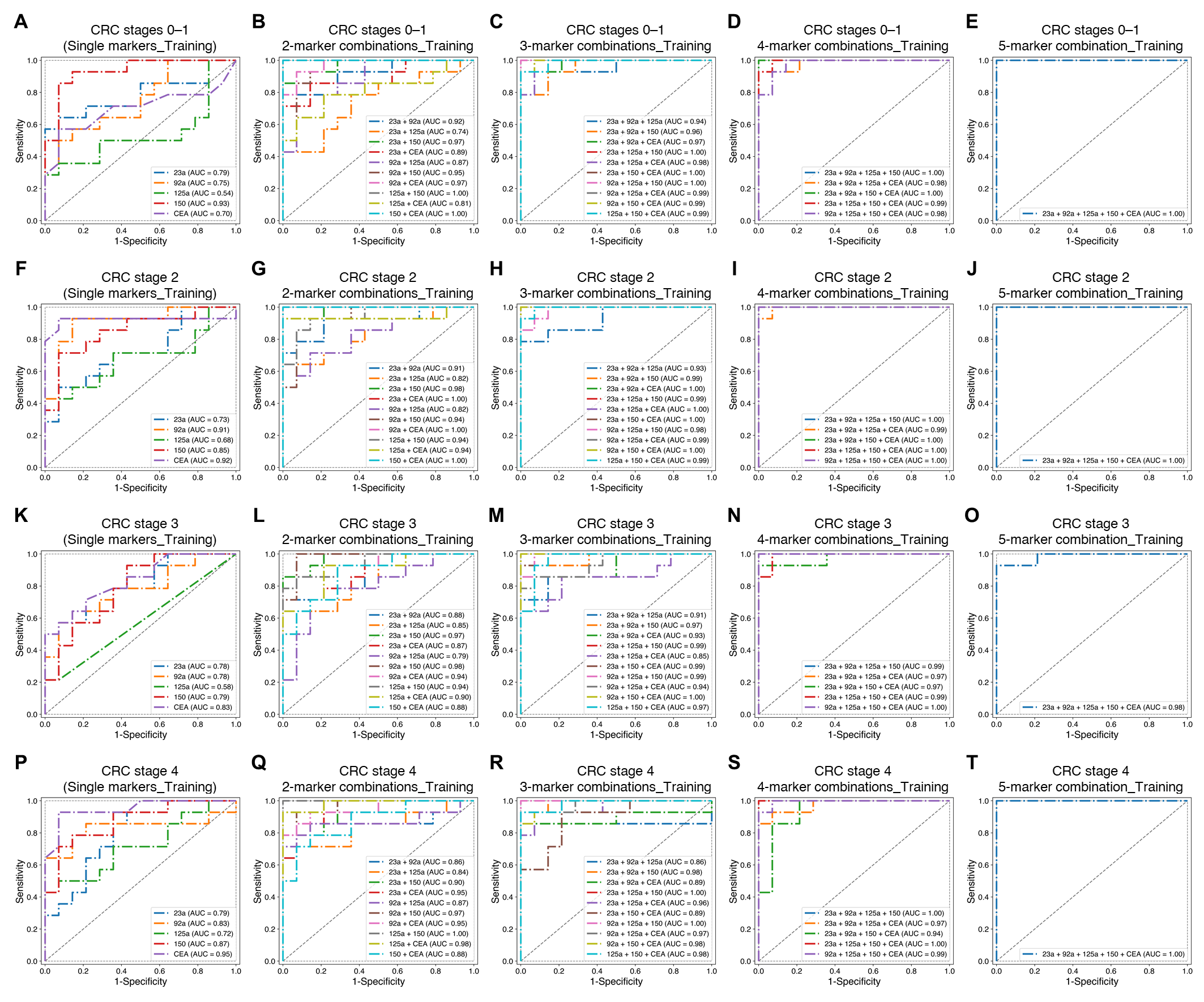


**Figure S20.** ROC curves and AUC values of the training set for blood biomarker combinations in individual CRC stages. (A–E) ROC curves showing the diagnostic performance of single markers (A), 2-marker combinations (B), 3-marker combinations (C), 4-marker combinations (D), and a 5-marker combination (E) in the training set for CRC stages 0–1 diagnosis. (F–J) ROC curves showing the diagnostic performance of single markers (F), 2-marker combinations (G), 3-marker combinations (H), 4-marker combinations (I), and a 5-marker combination (J) in the training set for CRC stage 2 diagnosis. (K–O) ROC curves showing the diagnostic performance of single markers (K), 2-marker combinations (L), 3-marker combinations (M), 4-marker combinations (N), and a 5-marker combination (O) in the training set for CRC stage 3 diagnosis. (P–T) ROC curves showing the diagnostic performance of single markers (P), 2-marker combinations (Q), 3-marker combinations (R), 4-marker combinations (S), and a 5-marker combination (T) in the training set for CRC stage 4 diagnosis. Each plot includes the AUC values for the corresponding biomarker combinations, evaluated using the deep learning model. The EV-derived miRNA markers are labeled simply as 23a, 92a, 125a, and 150 in (A–T).


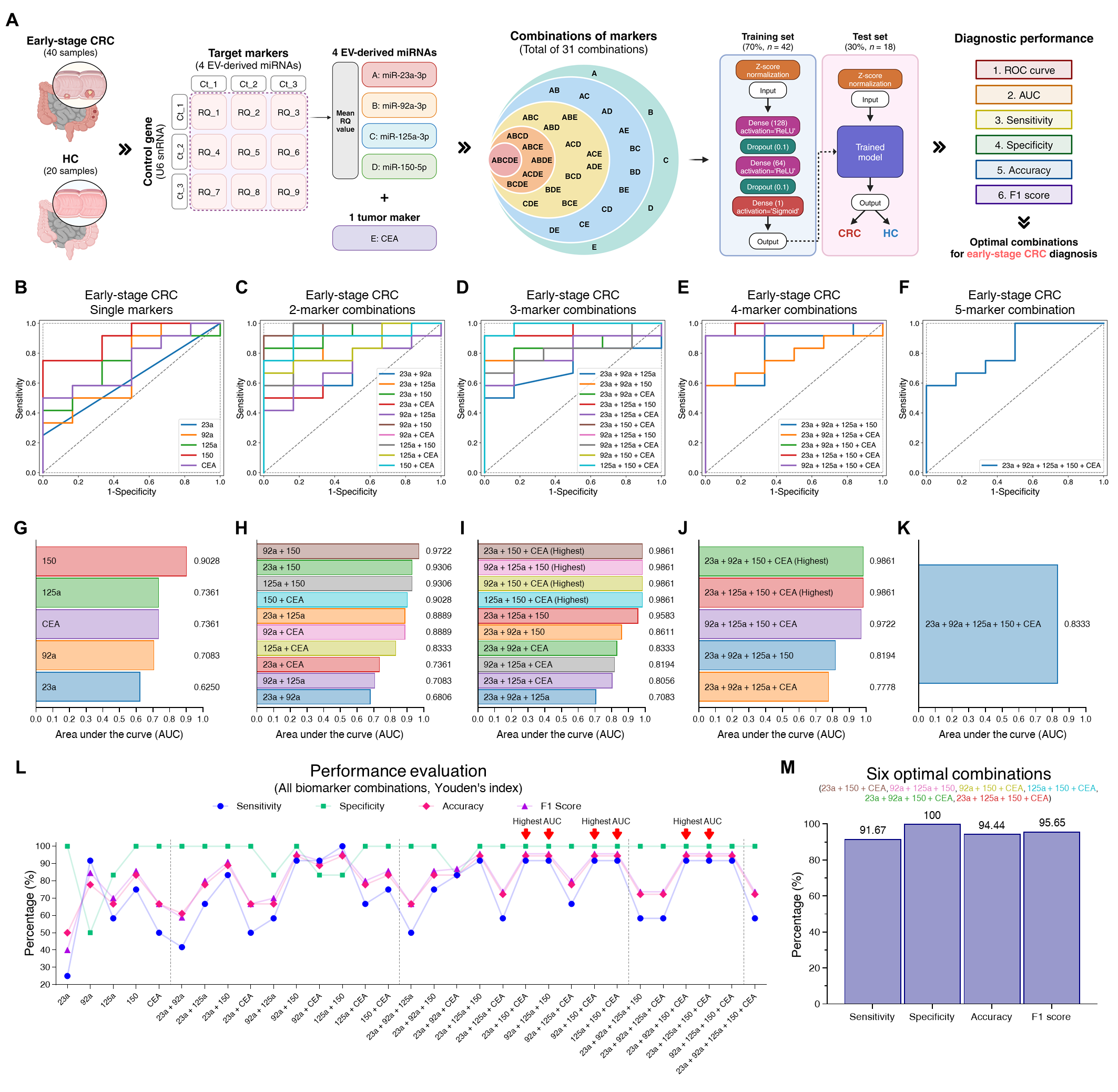


**Figure S21.** AI-driven analysis of blood biomarker combinations for early-stage CRC in the ZAHV-AI system. (A) Schematic overview of the ZAHV-AI system workflow for evaluating biomarker combinations for early-stage CRC. Samples were taken from 40 CRC stages 0–2 patients and 20 HC individuals, divided into a training set (70%, *n* = 42) and a test set (30%, *n* = 18). The deep learning model was evaluated for its diagnostic performance. Created with BioRender.com. (B–K) ROC curves and AUC values, ordered by performance from highest to lowest, for all biomarker combinations, including single markers (B, G), 2-marker combinations (C, H), 3-marker combinations (D, I), 4-marker combinations (E, J), and a 5-marker combination (F, K). (L) Performance evaluation for all biomarker combinations using Youden's index. (M) Bar chart showing performance metrics (sensitivity, specificity, accuracy, and F1 score) for the top six biomarker combinations (23a + 150 + CEA, 92a + 125a + 150, 92a + 150 + CEA, 125a + 150 + CEA, 23a + 92a + 150 + CEA, 23a + 125a + 150 + CEA). The EV-derived miRNA markers are labeled simply as 23a, 92a, 125a, and 150 in (B–M).


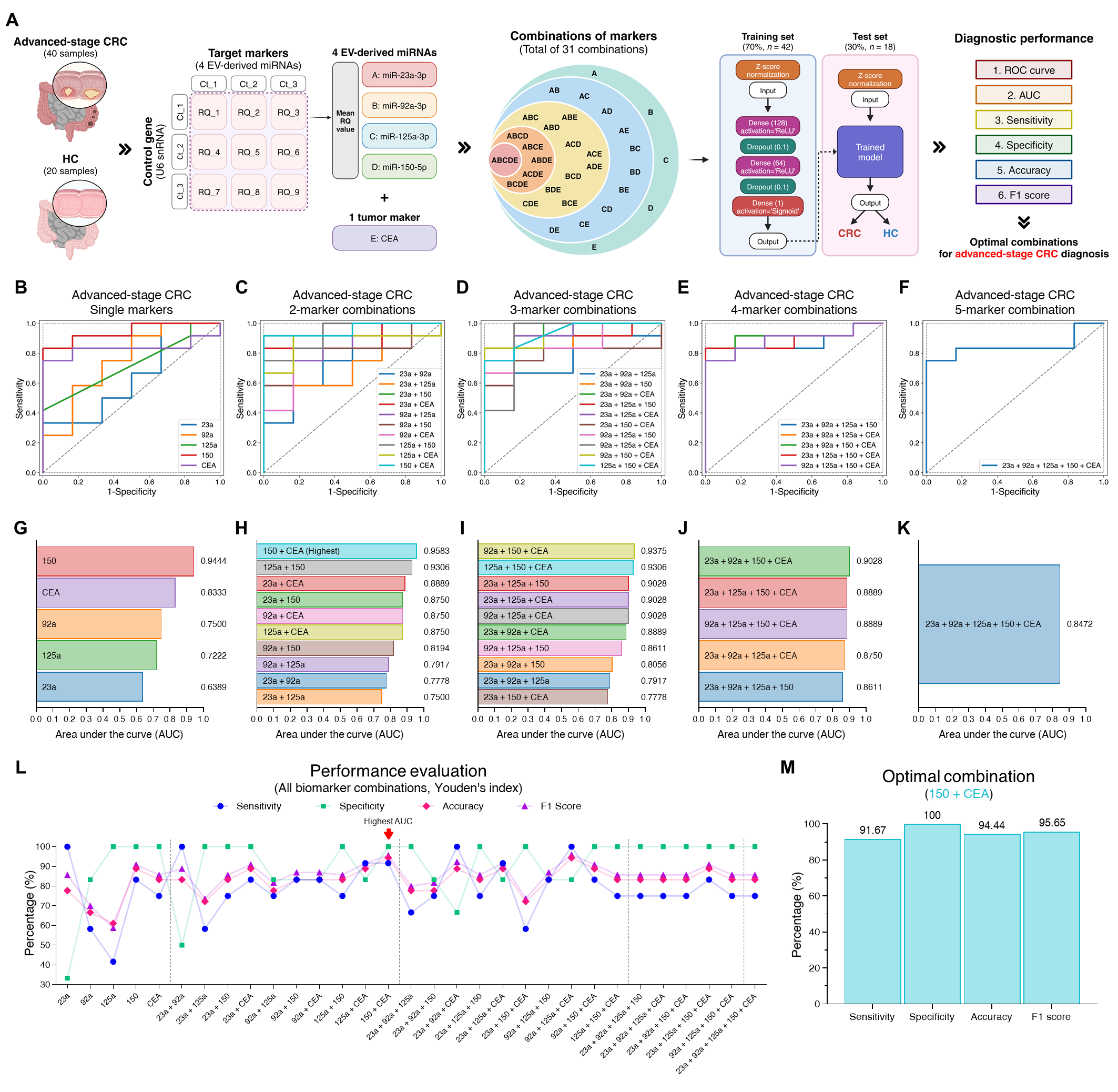


**Figure S22.** AI-driven analysis of blood biomarker combinations for advanced -stage CRC in the ZAHV-AI system. (A) Schematic overview of the ZAHV-AI system workflow for evaluating biomarker combinations for advanced-stage CRC. Samples were taken from 40 CRC stages 3–4 patients and 20 HC individuals, divided into a training set (70%, *n* = 42) and a test set (30%, *n* = 18). The deep learning model was evaluated for its diagnostic performance. Created with BioRender.com. (B–K) ROC curves and AUC values, ordered by performance from highest to lowest, for all biomarker combinations, including single markers (B, G), 2-marker combinations (C, H), 3-marker combinations (D, I), 4-marker combinations (E, J), and a 5-marker combination (F, K). (L) Performance evaluation for all biomarker combinations using Youden's index. (M) Bar chart showing performance metrics (sensitivity, specificity, accuracy, and F1 score) for the top biomarker combination (150 and CEA). The EV-derived miRNA markers are labeled simply as 23a, 92a, 125a, and 150 in (B–M).


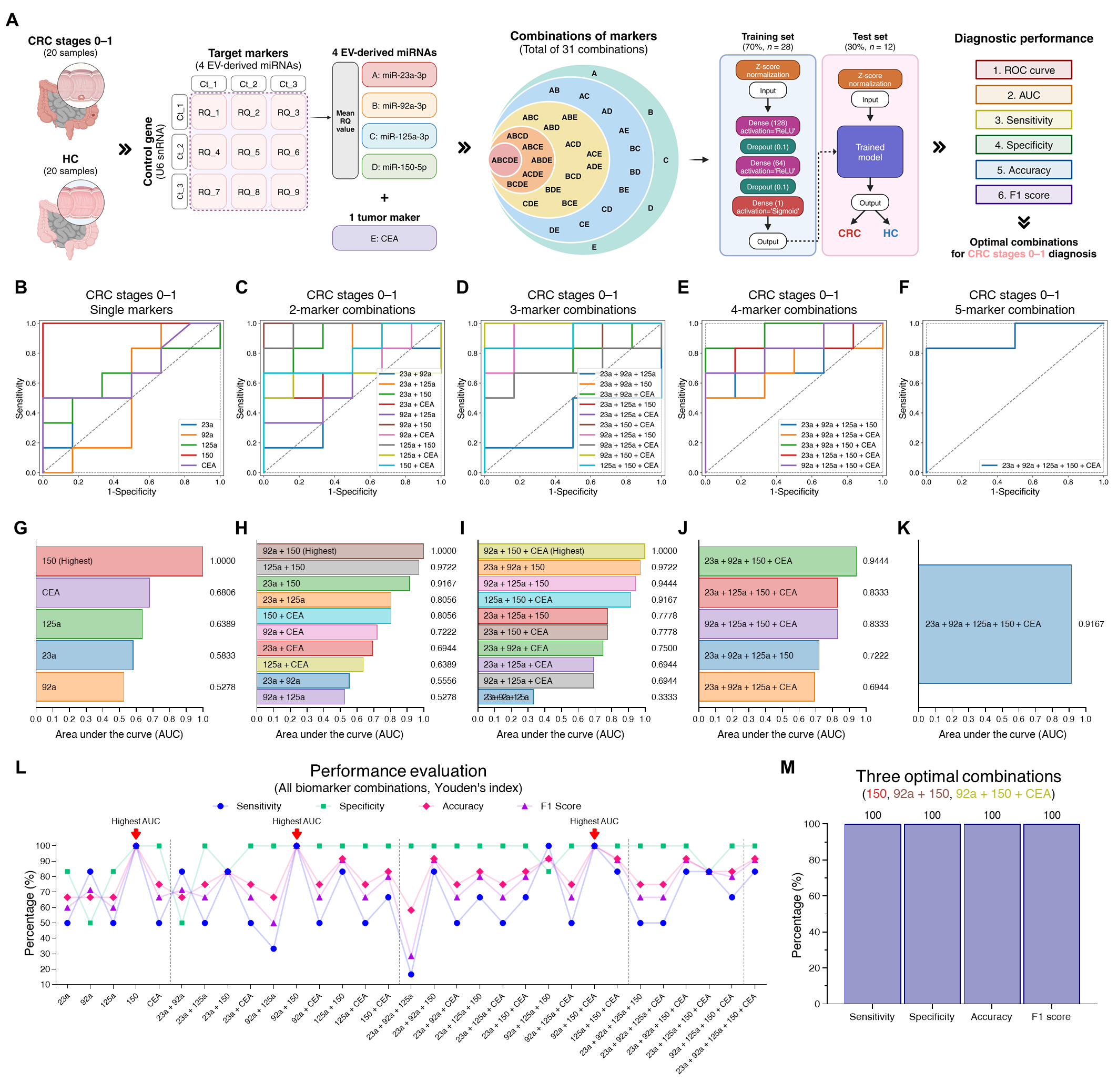


**Figure S23.** AI-driven analysis of blood biomarker combinations for CRC stages 0–1 in the ZAHV-AI system. (A) Schematic overview of the ZAHV-AI system workflow for evaluating biomarker combinations for CRC stages 0–1. Samples were taken from 20 CRC stages 0–1 patients and 20 HC individuals, divided into a training set (70%, *n* = 28) and a test set (30%, *n* = 12). The deep learning model was evaluated for its diagnostic performance. Created with BioRender.com. (B–K) ROC curves and AUC values, ordered by performance from highest to lowest, for all biomarker combinations, including single markers (B, G), 2-marker combinations (C, H), 3-marker combinations (D, I), 4-marker combinations (E, J), and a 5-marker combination (F, K). (L) Performance evaluation for all biomarker combinations using Youden's index. (M) Bar chart showing performance metrics (sensitivity, specificity, accuracy, and F1 score) for the top three biomarker combinations (150, 92a + 150, 92a + 150 + CEA). The EV-derived miRNA markers are labeled simply as 23a, 92a, 125a, and 150 in (B–M).


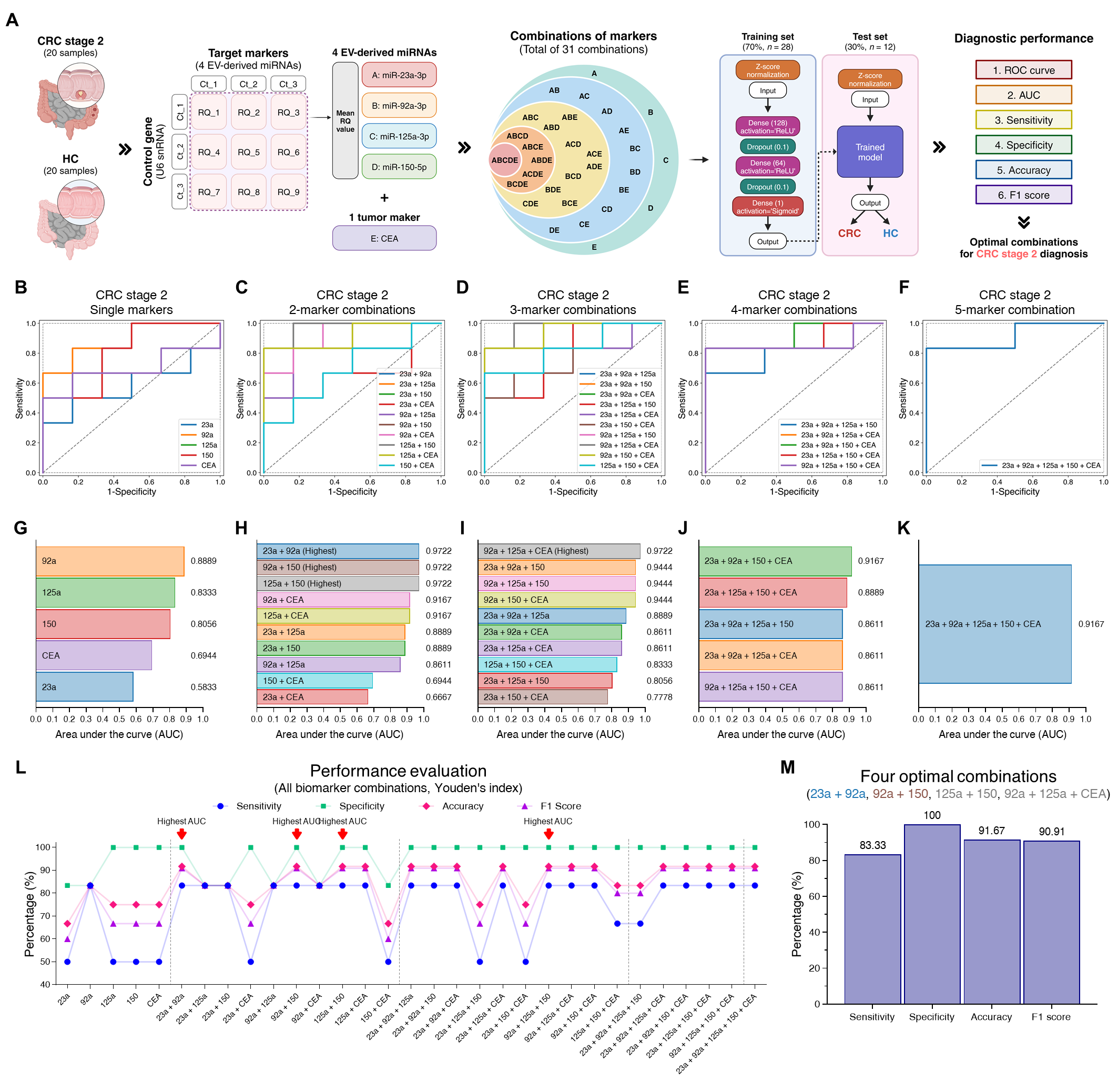


**Figure S24.** AI-driven analysis of blood biomarker combinations for CRC stage 2 in the ZAHV-AI system. (A) Schematic overview of the ZAHV-AI system workflow for evaluating biomarker combinations for CRC stage 2. Samples were taken from 20 CRC stage 2 patients and 20 HC individuals, divided into a training set (70%, *n* = 28) and a test set (30%, *n* = 12). The deep learning model was evaluated for its diagnostic performance. Created with BioRender.com. (B–K) ROC curves and AUC values, ordered by performance from highest to lowest, for all biomarker combinations, including single markers (B, G), 2-marker combinations (C, H), 3-marker combinations (D, I), 4-marker combinations (E, J), and a 5-marker combination (F, K). (L) Performance evaluation for all biomarker combinations using Youden's index. (M) Bar chart showing performance metrics (sensitivity, specificity, accuracy, and F1 score) for the top four biomarker combinations (23a + 92a, 92a + 150, 125a + 150, 92a + 125a + CEA). The EV-derived miRNA markers are labeled simply as 23a, 92a, 125a, and 150 in (B–M).


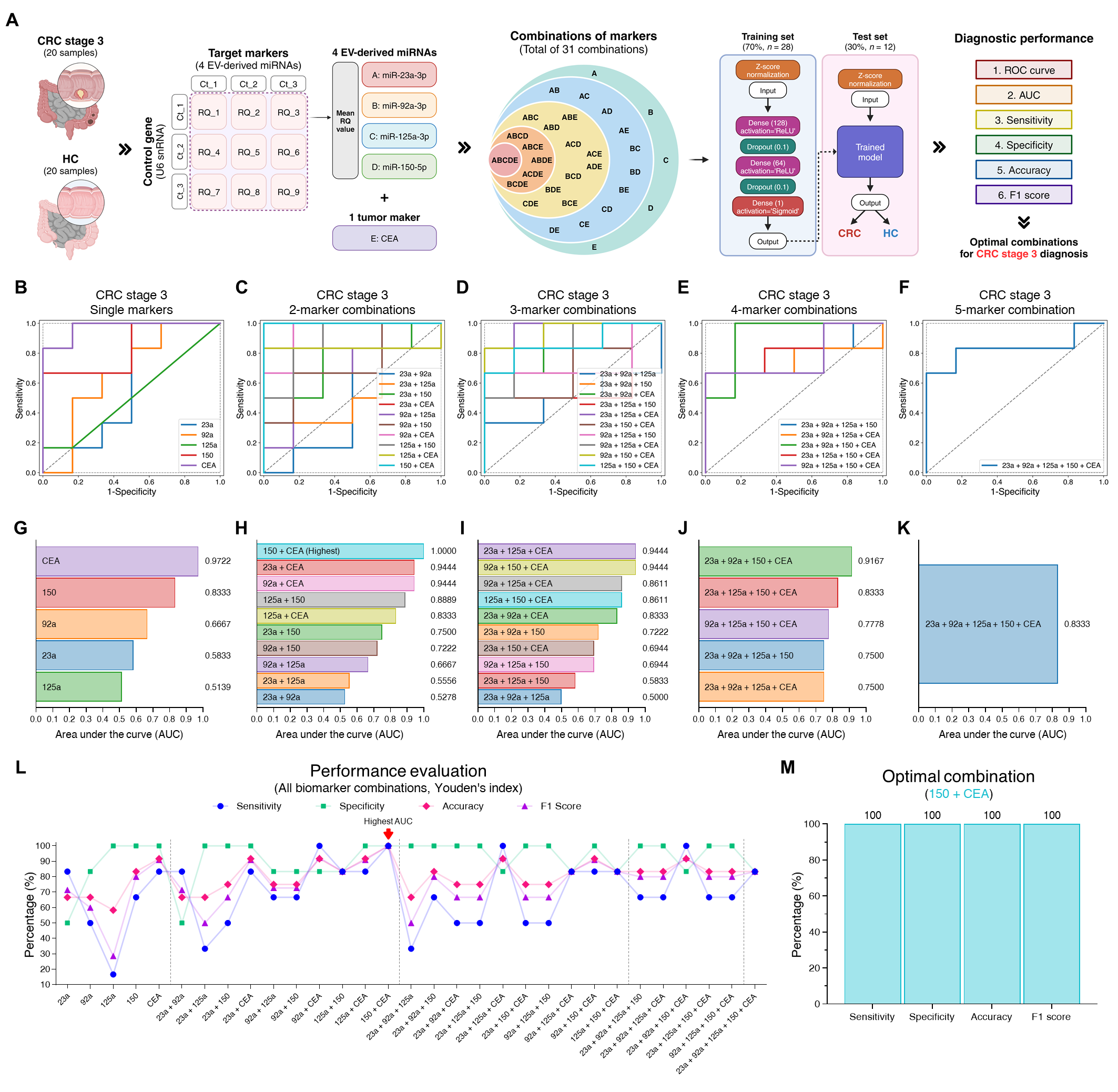


**Figure S25.** AI-driven analysis of blood biomarker combinations for CRC stage 3 in the ZAHV-AI system. (A) Schematic overview of the ZAHV-AI system workflow for evaluating biomarker combinations for CRC stage 3. Samples were taken from 20 CRC stage 3 patients and 20 HC individuals, divided into a training set (70%, *n* = 28) and a test set (30%, *n* = 12). The deep learning model was evaluated for its diagnostic performance. Created with BioRender.com. (B–K) ROC curves and AUC values, ordered by performance from highest to lowest, for all biomarker combinations, including single markers (B, G), 2-marker combinations (C, H), 3-marker combinations (D, I), 4-marker combinations (E, J), and a 5-marker combination (F, K). (L) Performance evaluation for all biomarker combinations using Youden's index. (M) Bar chart showing performance metrics (sensitivity, specificity, accuracy, and F1 score) for the top biomarker combination (150 and CEA). The EV-derived miRNA markers are labeled simply as 23a, 92a, 125a, and 150 in (B–M).


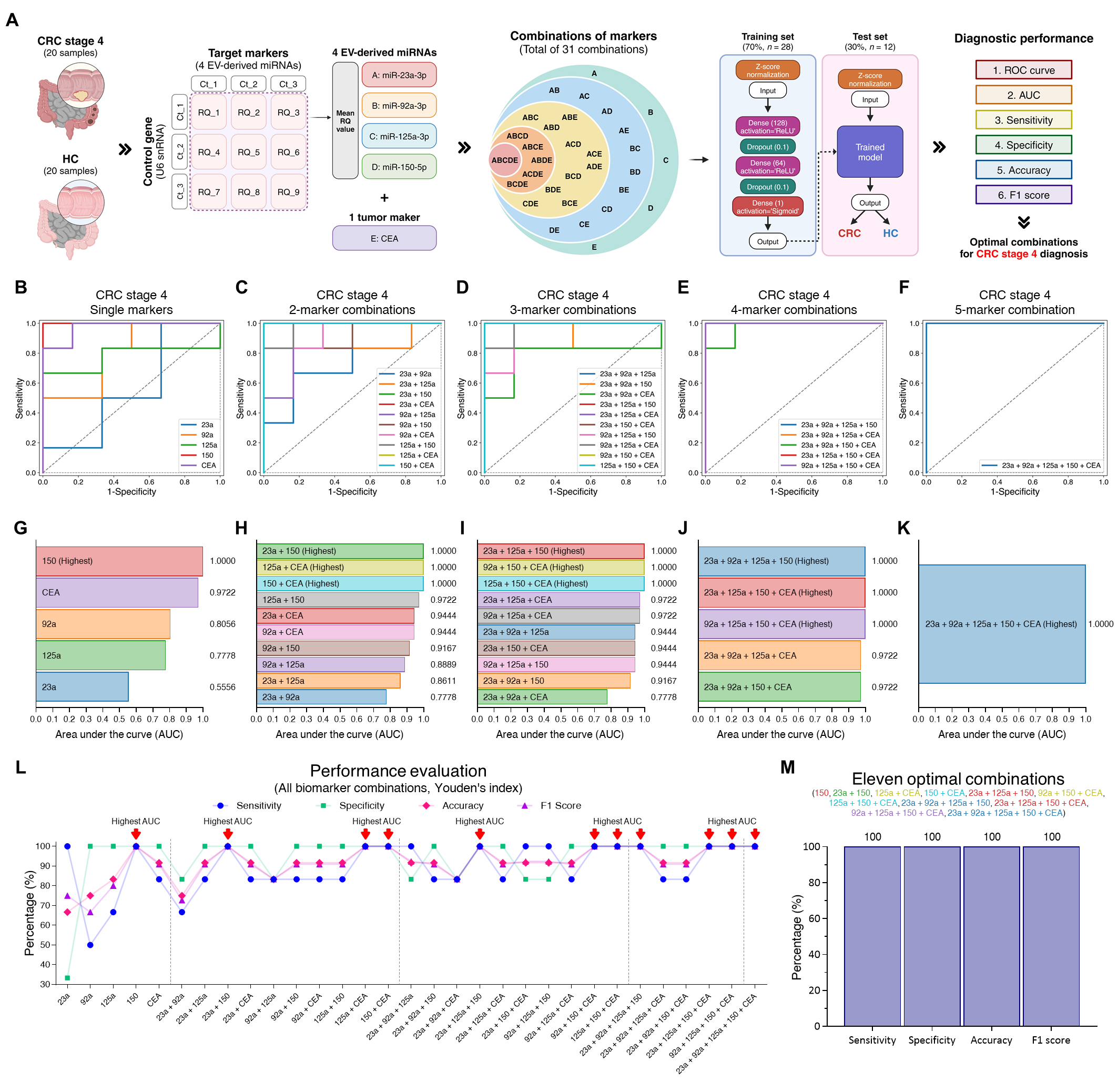


**Figure S26.** AI-driven analysis of blood biomarker combinations for CRC stage 4 in the ZAHV-AI system. (A) Schematic overview of the ZAHV-AI system workflow for evaluating biomarker combinations for CRC stage 4. Samples were taken from 20 CRC stage 4 patients and 20 HC individuals, divided into a training set (70%, *n* = 28) and a test set (30%, *n* = 12). The deep learning model was evaluated for its diagnostic performance. Created with BioRender.com. (B–K) ROC curves and AUC values, ordered by performance from highest to lowest, for all biomarker combinations, including single markers (B, G), 2-marker combinations (C, H), 3-marker combinations (D, I), 4-marker combinations (E, J), and a 5-marker combination (F, K). (L) Performance evaluation for all biomarker combinations using Youden's index. (M) Bar chart showing performance metrics (sensitivity, specificity, accuracy, and F1 score) for the top eleven biomarker combinations (150, 23a + 150, 125a + CEA, 150 + CEA, 23a + 125a + 150, 92a + 150 + CEA, 125a + 150 + CEA, 23a + 92a + 125a + 150, 23a + 125a + 150 + CEA, 92a + 125a + 150 + CEA, 23a + 92a + 125a + 150 + CEA). The EV-derived miRNA markers are labeled simply as 23a, 92a, 125a, and 150 in (B–M).


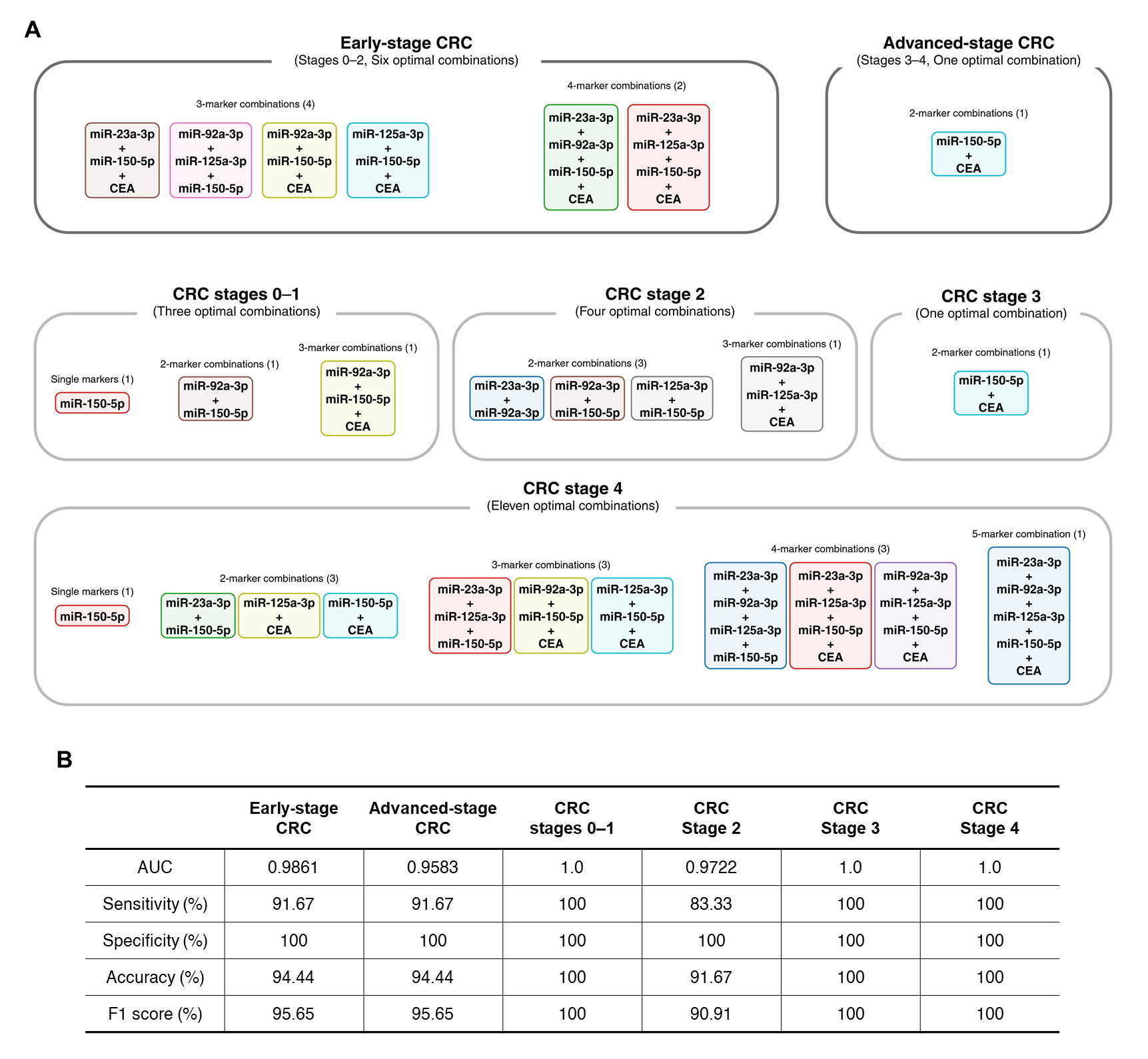


**Figure S27.** Optimal biomarker combinations and diagnostic performance for early-stage, advanced-stage, and individual CRC stages in the ZAHV-AI system. (A) Schematic overview of the optimal blood biomarker combinations identified for early-stage CRC (stages 0–2), advanced-stage CRC (stages 3–4), and individual CRC stages (stages 0–1, stage 2, stage 3, and stage 4). Each section lists the selected combinations of miRNAs and CEA. Created with BioRender.com. (B) Diagnostic performance metrics (AUC, sensitivity, specificity, accuracy, F1 score) for each subgroup analysis.


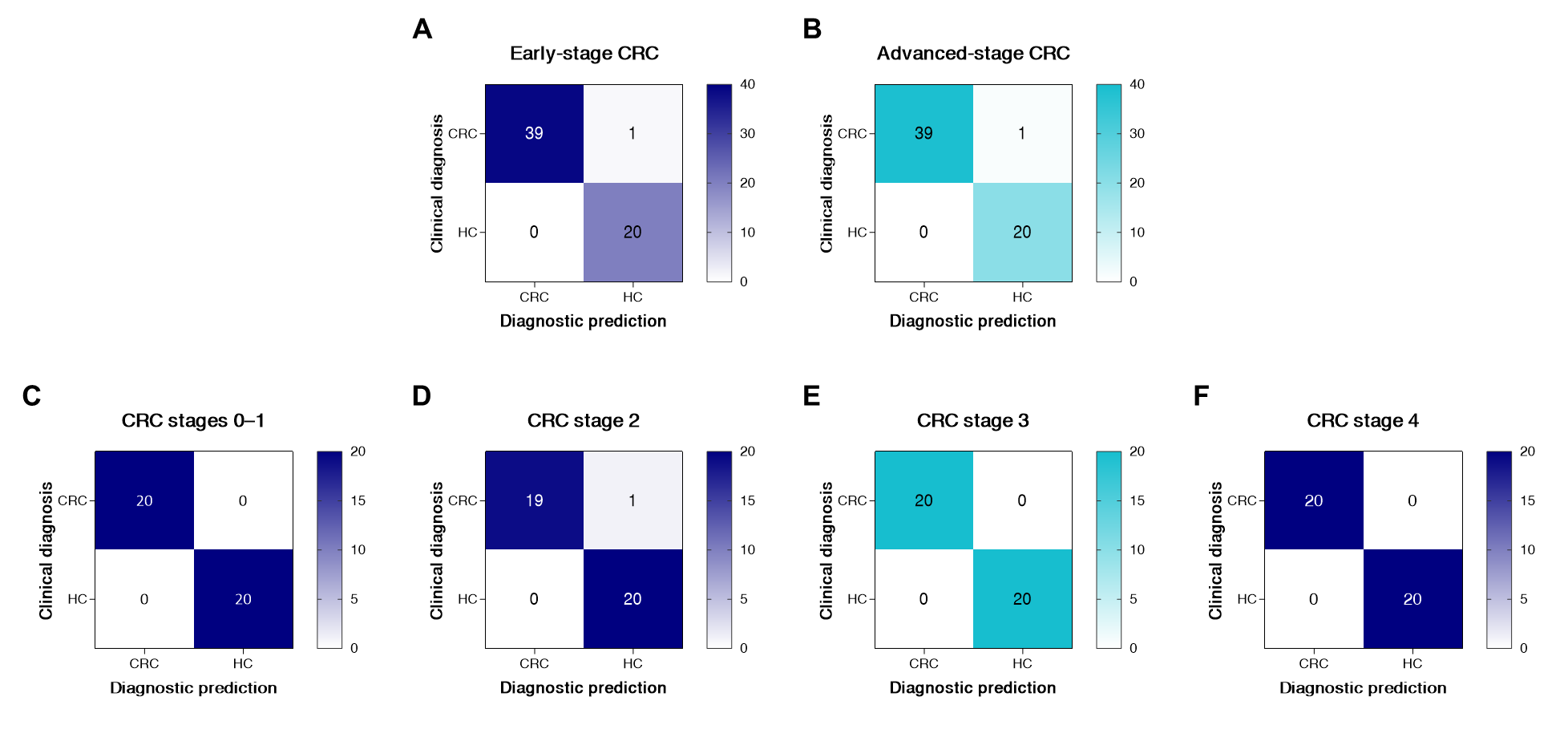


**Figure S28.** Confusion matrices for diagnostic prediction using optimal combinations. (A–F) Confusion matrices showing the diagnostic predictions for different CRC stages using the ZAHV-AI system, including early-stage CRC (A), advanced-stage CRC (B), CRC stages 0–1 (C), CRC stage 2 (D), CRC stage 3 (E), and CRC stage 4 (F).

**Table S1.** Primer sets for EV-derived biomarker candidates associated with CRC used in this study.

| **Primer list** | | **Location** | **Sequence (5'–3')** | **Length**  **(base)** |
| --- | --- | --- | --- | --- |
| **EV-derived**  **miRNAs** | **hsa-miR-19a-3p** | Forward | TGT GCA AAT CTA TGC AAA ACT GA | 23 |
|  | **hsa-miR-21-5p** | Forward | TAG CTT ATC AGA CTG ATG TTG A | 22 |
|  | **hsa-miR-23a-3p** | Forward | ATC ACA TTG CCA GGG ATT TCC | 21 |
|  | **hsa-miR-92a-3p** | Forward | TAT TGC ACT TGT CCC GGC CTG T | 22 |
|  | **hsa-miR-99b-5p** | Forward | CAC CCG TAG AAC CGA CCT TGC G | 22 |
|  | **hsa-miR-122-5p** | Forward | TGG AGT GTG ACA ATG GTG TTT G | 22 |
|  | **hsa-miR-125a-3p** | Forward | ACA GGT GAG GTT CTT GGG AGC C | 22 |
|  | **hsa-miR-141-3p** | Forward | TAA CAC TGT CTG GTA AAG ATG G | 22 |
|  | **hsa-miR-150-5p** | Forward | TCT CCC AAC CCT TGT ACC AGT G | 22 |
|  | **hsa-miR-181a-5p** | Forward | AAC ATT CAA CGC TGT CGG TGA GT | 23 |
|  | **hsa-miR-182-5p** | Forward | TTT GGC AAT GGT AGA ACT CAC ACT | 24 |
|  | **hsa-miR-200b-3p** | Forward | TAA TAC TGC CTG GTA ATG ATG A | 22 |
|  | **hsa-miR-222-5p** | Forward | CTC AGT AGC CAG TGT AGA TCC T | 22 |
|  | **hsa-miR-223-3p** | Forward | TGT CAG TTT GTC AAA TAC CCC A | 22 |
|  | **hsa-miR-1246** | Forward | AAT GGA TTT TTG GAG CAG G | 19 |
|  | **Universal** | Reverse | mRQ 3' Primer from TAKARA | . |
|  | **U6** | Forward | U6 Forward Primer from TAKARA |  |
|  |  | Reverse | U6 Reverse Primer from TAKARA |  |
| **EV-derived**  **circRNAs** | **circLONP2**  (hsa_circ_0008558) | Forward | GTG AAG GTG GCA GAA GGA CA | 20 |
|  |  | Reverse | TGG GTT GTT CAC TCC CAC AG | 20 |
|  | **circPNN**  (hsa_circ_0101802) | Forward | CCT GGA AGA ATG TGT CCA GCT A | 22 |
|  |  | Reverse | GCT TTC TCT CTT CTT CTG CCT G | 22 |
|  | **circLPAR1**  (has_circ_0087960) | Forward | GTA GTT CTG GGG CGT GTT CA | 20 |
|  |  | Reverse | TAG GTG GAT GGG GAG CTT CA | 20 |
|  | **GAPDH** | Forward | TAT CGT GAT GCT AGT CCG ATG | 21 |
|  |  | Reverse | TGC AGC TAG CTG CAT CGA TCG G | 22 |

**Table S2.** Distribution of biomarker values in training and test cohorts for CRC and HC groups using splitting method.

|  |  | **Biomarker values in training and test cohorts**  [Mean (SD)] | | | | | |
| --- | --- | --- | --- | --- | --- | --- | --- |
| **Clinical**  **group** | **Cohorts** | | miR-23a-3p  (RQ) | miR-23a-3p  (RQ) | miR-23a-3p  (RQ) | miR-150-5p  (RQ) | CEA  (ng mL^−1^) |
| **HC** | Training  (*n* = 14) | | 1.33 (1.28) | 1.52 (2.09) | 1.13 (0.54) | 1.13 (0.44) | 1.41 (0.43) |
|  | Test  (*n* = 6) | | 1.44 (0.94) | 1.64 (1.32) | 1.18 (0.71) | 1.05 (0.35) | 1.46 (0.48) |
| **Overall**  **CRC** | Training  (*n* = 56) | | 4.44 (6.44) | 5.12 (7.11) | 2.71 (3.57) | 0.55 (0.33) | 10.88 (36.47) |
|  | Test  (*n* = 24) | | 5.08 (8.42) | 5.42 (6.38) | 3.16 (3.79) | 0.55 (0.24) | 10.79 (25.16) |
| **Early-stage**  **CRC** | Training  (*n* = 28) | | 5.23 (7.15) | 6.09 (9.07) | 2.99 (4.37) | 0.50 (0.33) | 4.34 (6.03) |
|  | Test  (*n* = 12) | | 5.06 (6.65) | 6.69 (8.13) | 3.71 (5.00) | 0.57 (0.23) | 4.14 (5.92) |
| **Advanced-stage**  **CRC** | Training  (*n* = 28) | | 3.65 (5.65) | 4.15 (4.35) | 2.42 (2.60) | 0.60 (0.33) | 17.43 (50.83) |
|  | Test  (*n* = 12) | | 5.09 (10.20) | 4.14 (3.92) | 2.61 (2.10) | 0.52 (0.26) | 17.44 (34.52) |
| **CRC**  **stages 0–1** | Training  (*n* = 14) | | 5.16 (6.06) | 4.30 (8.13) | 1.99 (1.85) | 0.45 (0.22) | 1.91 (1.49) |
|  | Test  (*n* = 6) | | 4.87 (7.12) | 3.86 (3.73) | 2.06 (1.51) | 0.47 (0.10) | 2.10 (0.83) |
| **CRC**  **stage 2** | Training  (*n* = 14) | | 5.30 (8.34) | 7.87 (9.89) | 3.99 (5.83) | 0.54 (0.42) | 6.77 (7.78) |
|  | Test  (*n* = 6) | | 5.25 (6.83) | 9.53 (10.59) | 5.37 (6.80) | 0.67 (0.28) | 6.17 (8.16) |
| **CRC**  **stage 3** | Training  (*n* = 14) | | 2.53 (2.32) | 3.04 (2.38) | 1.83 (2.01) | 0.65 (0.34) | 4.82 (9.75) |
|  | Test  (*n* = 6) | | 3.07 (2.62) | 2.87 (2.17) | 1.62 (0.91) | 0.56 (0.34) | 5.03 (4.75) |
| **CRC**  **stage 4** | Training  (*n* = 14) | | 4.77 (7.63) | 5.26 (5.56) | 3.01 (3.04) | 0.54 (0.33) | 30.04 (70.21) |
|  | Test  (*n* = 6) | | 7.11 (14.58) | 5.41 (5.03) | 3.60 (2.55) | 0.48 (0.19) | 29.85 (47.21) |

**Table S3.** Diagnostic performance of 31 blood biomarker combinations by ZAHV-AI system for early-stage CRC.

| **Biomarker combinations**^a)^  (Total of 31 combinations) | | **Test set (*n* = 18)** | | | | |
| --- | --- | --- | --- | --- | --- | --- |
|  |  | **AUC**  (95% CI)^c)^ | **Sensitivity**  (%) | **Specificity**  (%) | **Accuracy**  (%) | **F1 Score**  (%) |
| **Single**  **markers** | 23a | 0.6250  (0.5000–0.7500) | 25.00 | 100 | 50.00 | 40.00 |
|  | 92a | 0.7083  (0.4286–0.9688) | 91.67 | 50.00 | 77.78 | 84.62 |
|  | 125a | 0.7361  (0.4642–0.9625) | 58.33 | 83.33 | 66.67 | 70.00 |
|  | 150 | 0.9028  (0.7231–1.0) | 75.00 | 100 | 83.33 | 85.71 |
|  | CEA | 0.7361  (0.4545–0.9500) | 50.00 | 100 | 66.67 | 66.67 |
| **2-marker combinations** | 23a + 92a | 0.6806  (0.3750–0.9231) | 41.67 | 100 | 61.11 | 58.82 |
|  | 23a + 125a | 0.8889  (0.7013–1.0) | 66.67 | 100 | 77.78 | 80.00 |
|  | 23a + 150 | 0.9306  (0.7778–1.0) | 83.33 | 100 | 88.89 | 90.91 |
|  | 23a + CEA | 0.7361  (0.4769–0.9481) | 50.00 | 100 | 66.67 | 66.67 |
|  | 92a + 125a | 0.7083  (0.4675–0.9334) | 58.33 | 83.33 | 66.67 | 70.00 |
|  | 92a + 150 | 0.9722  (0.8750–1.0) | 91.67 | 100 | 94.44 | 95.65 |
|  | 92a + CEA | 0.8889  (0.6875–1.0) | 91.67 | 83.33 | 88.89 | 91.67 |
|  | 125a + 150 | 0.9306  (0.7143–1.0) | 100 | 83.33 | 94.44 | 96.00 |
|  | 125a + CEA | 0.8333  (0.5974–1.0) | 66.67 | 100 | 77.78 | 80.00 |
|  | 150 + CEA | 0.9028  (0.7385–1.0) | 75.00 | 100 | 83.33 | 85.71 |
| **3-marker combinations** | 23a + 92a + 125a | 0.7083  (0.4444–0.9333) | 50.00 | 100 | 66.67 | 66.67 |
|  | 23a + 92a + 150 | 0.8611  (0.6429–1.0) | 75.00 | 100 | 83.33 | 85.71 |
|  | 23a + 92a + CEA | 0.8333  (0.6234–1.0) | 83.33 | 83.33 | 83.33 | 86.96 |
|  | 23a + 125a + 150 | 0.9583  (0.8499–1.0) | 91.67 | 100 | 94.44 | 95.65 |
|  | 23a + 125a + CEA | 0.8056  (0.5846–1.0) | 58.33 | 100 | 72.22 | 73.68 |
|  | 23a + 150 + CEA^b)^ | 0.9861  (0.9218–1.0) | 91.67 | 100 | 94.44 | 95.65 |
|  | 92a + 125a + 150^b)^ | 0.9861  (0.9286–1.0) | 91.67 | 100 | 94.44 | 95.65 |
|  | 92a + 125a + CEA | 0.8194  (0.6049–1.0) | 66.67 | 100 | 77.78 | 80.00 |
|  | 92a + 150 + CEA^b)^ | 0.9861  (0.9221–1.0) | 91.67 | 100 | 94.44 | 95.65 |
|  | 125a + 150 + CEA^b)^ | 0.9861  (0.9221–1.0) | 91.67 | 100 | 94.44 | 95.65 |
| **4-marker combinations** | 23a + 92a + 125a + 150 | 0.8194  (0.5844–1.0) | 58.33 | 100 | 72.22 | 73.68 |
|  | 23a + 92a + 125a + CEA | 0.7778  (0.5432–0.9741) | 58.33 | 100 | 72.22 | 73.68 |
|  | 23a + 92a + 150 + CEA^b)^ | 0.9861  (0.9249–1.0) | 91.67 | 100 | 94.44 | 95.65 |
|  | 23a + 125a + 150 + CEA^b)^ | 0.9861  (0.9167–1.0) | 91.67 | 100 | 94.44 | 95.65 |
|  | 92a + 125a + 150 + CEA | 0.9722  (0.8765–1.0) | 91.67 | 100 | 94.44 | 95.65 |
| **5-marker combinations** | 23a + 92a + 125a + 150 + CEA | 0.8472  (0.6997–1.0) | 75.00 | 100 | 83.33 | 85.71 |

^a)^The EV-derived miRNAs are labeled simply as 23a, 92a, 125a, and 150.

^b)^Six optimal combinations.

^c)^95% CI, 95% confidence interval.

**Table S4.** Diagnostic performance of 31 blood biomarker combinations by ZAHV-AI system for advanced-stage CRC.

| **Biomarker combinations**^a)^  (Total of 31 combinations) | | **Test set (*n* = 18)** | | | | |
| --- | --- | --- | --- | --- | --- | --- |
|  |  | **AUC**  (95% CI)^c)^ | **Sensitivity**  (%) | **Specificity**  (%) | **Accuracy**  (%) | **F1 Score**  (%) |
| **Single**  **markers** | 23a | 0.6389  (0.3194–0.8963) | 100 | 33.33 | 77.78 | 85.71 |
|  | 92a | 0.7500  (0.4567–0.9846) | 58.33 | 83.33 | 66.67 | 70.00 |
|  | 125a | 0.7222  (0.5357–0.8889) | 41.67 | 100 | 61.11 | 58.82 |
|  | 150 | 0.9444  (0.8154–1.0) | 83.33 | 100 | 88.89 | 90.91 |
|  | CEA | 0.8333  (0.6153–1.0) | 75.00 | 100 | 83.33 | 85.71 |
| **2-marker combinations** | 23a + 92a | 0.7778  (0.4768–1.0) | 100 | 50.00 | 83.33 | 88.89 |
|  | 23a + 125a | 0.7500  (0.4667–0.9584) | 58.33 | 100 | 72.22 | 73.68 |
|  | 23a + 150 | 0.8750  (0.6750–1.0) | 75.00 | 100 | 83.33 | 85.71 |
|  | 23a + CEA | 0.8889  (0.7000–1.0) | 83.33 | 100 | 88.89 | 90.91 |
|  | 92a + 125a | 0.7917  (0.5325–0.9883) | 75.00 | 83.33 | 77.78 | 81.82 |
|  | 92a + 150 | 0.8194  (0.5974–1.0) | 83.33 | 83.33 | 83.33 | 86.96 |
|  | 92a + CEA | 0.8750  (0.6308–1.0) | 83.33 | 83.33 | 83.33 | 86.96 |
|  | 125a + 150 | 0.9306  (0.7692–1.0) | 75.00 | 100 | 83.33 | 85.71 |
|  | 125a + CEA | 0.8750  (0.6500–1.0) | 91.67 | 83.33 | 88.89 | 91.67 |
|  | 150 + CEA^b)^ | 0.9583  (0.8329–1.0) | 91.67 | 100 | 94.44 | 95.65 |
| **3-marker combinations** | 23a + 92a + 125a | 0.7917  (0.5694–0.9740) | 66.67 | 100 | 77.78 | 80.00 |
|  | 23a + 92a + 150 | 0.8056  (0.5538–0.9877) | 75.00 | 83.33 | 77.78 | 81.82 |
|  | 23a + 92a + CEA | 0.8889  (0.6768–1.0) | 100 | 66.67 | 88.89 | 92.31 |
|  | 23a + 125a + 150 | 0.9028  (0.7083–1.0) | 75.00 | 100 | 83.33 | 85.71 |
|  | 23a + 125a + CEA | 0.9028  (0.6769–1.0) | 91.67 | 83.33 | 88.89 | 91.67 |
|  | 23a + 150 + CEA | 0.7778  (0.5357–0.9821) | 58.33 | 100 | 72.22 | 73.68 |
|  | 92a + 125a + 150 | 0.8611  (0.6470–1.0) | 83.33 | 83.33 | 83.33 | 86.96 |
|  | 92a + 125a + CEA | 0.9028  (0.6767–1.0) | 100 | 83.33 | 94.44 | 96.00 |
|  | 92a + 150 + CEA | 0.9375  (0.7917–1.0) | 83.33 | 100 | 88.89 | 90.91 |
|  | 125a + 150 + CEA | 0.9306  (0.7778–1.0) | 75.00 | 100 | 83.33 | 85.71 |
| **4-marker combinations** | 23a + 92a + 125a + 150 | 0.8611  (0.6667–1.0) | 75.00 | 100 | 83.33 | 85.71 |
|  | 23a + 92a + 125a + CEA | 0.8750  (0.6785–1.0) | 75.00 | 100 | 83.33 | 85.71 |
|  | 23a + 92a + 150 + CEA | 0.9028  (0.6785–1.0) | 75.00 | 100 | 83.33 | 85.71 |
|  | 23a + 125a + 150 + CEA | 0.8889  (0.7110–1.0) | 83.33 | 100 | 88.89 | 90.91 |
|  | 92a + 125a + 150 + CEA | 0.8889  (0.6963–1.0) | 75.00 | 100 | 83.33 | 85.71 |
| **5-marker combinations** | 23a + 92a + 125a + 150 + CEA | 0.8472  (0.6997–1.0) | 75.00 | 100 | 83.33 | 85.71 |

^a)^The EV-derived miRNAs are labeled simply as 23a, 92a, 125a, and 150.

^b)^One optimal combinations.

^c)^95% CI, 95% confidence interval.

**Table S5.** Diagnostic performance of 31 blood biomarker combinations by ZAHV-AI system for CRC stages 0–1.

| **Biomarker combinations**^a)^  (Total of 31 combinations) | | **Test set (*n* = 12)** | | | | |
| --- | --- | --- | --- | --- | --- | --- |
|  |  | **AUC**  (95% CI)^c)^ | **Sensitivity**  (%) | **Specificity**  (%) | **Accuracy**  (%) | **F1 Score**  (%) |
| **Single**  **markers** | 23a | 0.5833  (0.2222–0.9259) | 50.00 | 83.33 | 66.67 | 60.00 |
|  | 92a | 0.5278  (0.1499–0.9375) | 83.33 | 50.00 | 66.67 | 71.43 |
|  | 125a | 0.6389  (0.2812–0.9722) | 50.00 | 83.33 | 66.67 | 60.00 |
|  | 150^b)^ | 1.0  (1.0–1.0) | 100 | 100 | 100 | 100 |
|  | CEA | 0.6806  (0.3056–1.0) | 50.00 | 100 | 75.00 | 66.67 |
| **2-marker combinations** | 23a + 92a | 0.5556  (0.1875–0.8889) | 83.33 | 50.00 | 66.67 | 71.43 |
|  | 23a + 125a | 0.8056  (0.4815–1.0) | 50.00 | 100 | 75.00 | 66.67 |
|  | 23a + 150 | 0.9167  (0.6875–1.0) | 83.33 | 83.33 | 83.33 | 83.33 |
|  | 23a + CEA | 0.6944  (0.3429–1.0) | 50.00 | 100 | 75.00 | 66.67 |
|  | 92a + 125a | 0.5278  (0.1250–0.9065) | 33.33 | 100 | 66.67 | 50.00 |
|  | 92a + 150^b)^ | 1.0  (1.0–1.0) | 100 | 100 | 100 | 100 |
|  | 92a + CEA | 0.7222  (0.3437–1.0) | 50.00 | 100 | 75.00 | 66.67 |
|  | 125a + 150 | 0.9722  (0.8286–1.0) | 83.33 | 100 | 91.67 | 90.91 |
|  | 125a + CEA | 0.6389  (0.2000–1.0) | 50.00 | 100 | 75.00 | 66.67 |
|  | 150 + CEA | 0.8056  (0.4568–1.0) | 66.67 | 100 | 83.33 | 80.00 |
| **3-marker combinations** | 23a + 92a + 125a | 0.3333  (0.0–0.7500) | 16.67 | 100 | 58.33 | 28.57 |
|  | 23a + 92a + 150 | 0.9722  (0.8286–1.0) | 83.33 | 100 | 91.67 | 90.91 |
|  | 23a + 92a + CEA | 0.7500  (0.3429–1.0) | 50.00 | 100 | 75.00 | 66.67 |
|  | 23a + 125a + 150 | 0.7778  (0.4286–1.0) | 66.67 | 100 | 83.33 | 80.00 |
|  | 23a + 125a + CEA | 0.6944  (0.3054–1.0) | 50.00 | 100 | 75.00 | 66.67 |
|  | 23a + 150 + CEA | 0.7778  (0.4286–1.0) | 66.67 | 100 | 83.33 | 80.00 |
|  | 92a + 125a + 150 | 0.9444  (0.7500–1.0) | 100 | 83.33 | 91.67 | 92.31 |
|  | 92a + 125a + CEA | 0.6944  (0.3125–1.0) | 50.00 | 100 | 75.00 | 66.67 |
|  | 92a + 150 + CEA^b)^ | 1.0  (1.0–1.0) | 100 | 100 | 100 | 100 |
|  | 125a + 150 + CEA | 0.9167  (0.7140–1.0) | 83.33 | 100 | 91.67 | 90.91 |
| **4-marker combinations** | 23a + 92a + 125a + 150 | 0.7222  (0.3630–1.0) | 50.00 | 100 | 75.00 | 66.67 |
|  | 23a + 92a + 125a + CEA | 0.6944  (0.3714–1.0) | 50.00 | 100 | 75.00 | 66.67 |
|  | 23a + 92a + 150 + CEA | 0.9444  (0.7500–1.0) | 83.33 | 100 | 91.67 | 90.91 |
|  | 23a + 125a + 150 + CEA | 0.8333  (0.4855–1.0) | 83.33 | 83.33 | 83.33 | 83.33 |
|  | 92a + 125a + 150 + CEA | 0.8333  (0.5429–1.0) | 66.67 | 100 | 83.33 | 80.00 |
| **5-marker combinations** | 23a + 92a + 125a + 150 + CEA | 0.9167  (0.6667–1.0) | 83.33 | 100 | 91.67 | 90.91 |

^a)^The EV-derived miRNAs are labeled simply as 23a, 92a, 125a, and 150.

^b)^Three optimal combinations.

^c)^95% CI, 95% confidence interval.

**Table S6.** Diagnostic performance of 31 blood biomarker combinations by ZAHV-AI system for CRC stage 2.

| **Biomarker combinations**^a)^  (Total of 31 combinations) | | **Test set (*n* = 12)** | | | | |
| --- | --- | --- | --- | --- | --- | --- |
|  |  | **AUC**  (95% CI)^c)^ | **Sensitivity**  (%) | **Specificity**  (%) | **Accuracy**  (%) | **F1 Score**  (%) |
| **Single**  **markers** | 23a | 0.5833  (0.1714–0.9429) | 50.00 | 83.33 | 66.67 | 60.00 |
|  | 92a | 0.8889  (0.6296–1.0) | 83.33 | 83.33 | 83.33 | 83.33 |
|  | 125a | 0.8333  (0.5312–1.0) | 50.00 | 100 | 75.00 | 66.67 |
|  | 150 | 0.8056  (0.4857–1.0) | 50.00 | 100 | 75.00 | 66.67 |
|  | CEA | 0.6944  (0.3329–1.0) | 50.00 | 100 | 75.00 | 66.67 |
| **2-marker combinations** | 23a + 92a^b)^ | 0.9722  (0.8519–1.0) | 83.33 | 100 | 91.67 | 90.91 |
|  | 23a + 125a | 0.8889  (0.6571–1.0) | 83.33 | 83.33 | 83.33 | 83.33 |
|  | 23a + 150 | 0.8889  (0.6250–1.0) | 83.33 | 83.33 | 83.33 | 83.33 |
|  | 23a + CEA | 0.6667  (0.2500–1.0) | 50.00 | 100 | 75.00 | 66.67 |
|  | 92a + 125a | 0.8611  (0.5556–1.0) | 83.33 | 83.33 | 83.33 | 83.33 |
|  | 92a + 150^b)^ | 0.9722  (0.8744–1.0) | 83.33 | 100 | 91.67 | 90.91 |
|  | 92a + CEA | 0.9167  (0.6997–1.0) | 83.33 | 83.33 | 83.33 | 83.33 |
|  | 125a + 150^b)^ | 0.9722  (0.8432–1.0) | 83.33 | 100 | 91.67 | 90.91 |
|  | 125a + CEA | 0.9167  (0.6667–1.0) | 83.33 | 100 | 91.67 | 90.91 |
|  | 150 + CEA | 0.6944  (0.3437–1.0) | 50.00 | 83.33 | 66.67 | 60.00 |
| **3-marker combinations** | 23a + 92a + 125a | 0.8889  (0.6250–1.0) | 83.33 | 100 | 91.67 | 90.91 |
|  | 23a + 92a + 150 | 0.9444  (0.7714–1.0) | 83.33 | 100 | 91.67 | 90.91 |
|  | 23a + 92a + CEA | 0.8611  (0.5553–1.0) | 83.33 | 100 | 91.67 | 90.91 |
|  | 23a + 125a + 150 | 0.8056  (0.4720–1.0) | 50.00 | 100 | 75.00 | 66.67 |
|  | 23a + 125a + CEA | 0.8611  (0.5000–1.0) | 83.33 | 100 | 91.67 | 90.91 |
|  | 23a + 150 + CEA | 0.7778  (0.4375–1.0) | 50.00 | 100 | 75.00 | 66.67 |
|  | 92a + 125a + 150 | 0.9444  (0.7500–1.0) | 83.33 | 100 | 91.67 | 90.91 |
|  | 92a + 125a + CEA^b)^ | 0.9722  (0.8333–1.0) | 83.33 | 100 | 91.67 | 90.91 |
|  | 92a + 150 + CEA | 0.9444  (0.7498–1.0) | 83.33 | 100 | 91.67 | 90.91 |
|  | 125a + 150 + CEA | 0.8333  (0.4996–1.0) | 66.67 | 100 | 83.33 | 80.00 |
| **4-marker combinations** | 23a + 92a + 125a + 150 | 0.8611  (0.5937–1.0) | 66.67 | 100 | 83.33 | 80.00 |
|  | 23a + 92a + 125a + CEA | 0.8611  (0.5000–1.0) | 83.33 | 100 | 91.67 | 90.91 |
|  | 23a + 92a + 150 + CEA | 0.9167  (0.6664–1.0) | 83.33 | 100 | 91.67 | 90.91 |
|  | 23a + 125a + 150 + CEA | 0.8889  (0.5833–1.0) | 83.33 | 100 | 91.67 | 90.91 |
|  | 92a + 125a + 150 + CEA | 0.8611  (0.5625–1.0) | 83.33 | 100 | 91.67 | 90.91 |
| **5-marker combinations** | 23a + 92a + 125a + 150 + CEA | 0.9167  (0.6570–1.0) | 83.33 | 100 | 91.67 | 90.91 |

^a)^The EV-derived miRNAs are labeled simply as 23a, 92a, 125a, and 150.

^b)^Four optimal combinations.

^c)^95% CI, 95% confidence interval.

**Table S7.** Diagnostic performance of 31 blood biomarker combinations by ZAHV-AI system for CRC stage 3.

| **Biomarker combinations**^a)^  (Total of 31 combinations) | | **Test set (*n* = 12)** | | | | |
| --- | --- | --- | --- | --- | --- | --- |
|  |  | **AUC**  (95% CI)^c)^ | **Sensitivity**  (%) | **Specificity**  (%) | **Accuracy**  (%) | **F1 Score**  (%) |
| **Single**  **markers** | 23a | 0.5833  (0.1845–0.9171) | 83.33 | 50.00 | 66.67 | 71.43 |
|  | 92a | 0.6667  (0.3125–0.9714) | 50.00 | 83.33 | 66.67 | 60.00 |
|  | 125a | 0.5139  (0.2500–0.7188) | 16.67 | 100 | 58.33 | 28.57 |
|  | 150 | 0.8333  (0.4834–1.0) | 66.67 | 100 | 83.33 | 80.00 |
|  | CEA | 0.9722  (0.8333–1.0) | 83.33 | 100 | 91.67 | 90.91 |
| **2-marker combinations** | 23a + 92a | 0.5278  (0.1667–0.9259) | 83.33 | 50.00 | 66.67 | 71.43 |
|  | 23a + 125a | 0.5556  (0.1667–0.8892) | 33.33 | 100 | 66.67 | 50.00 |
|  | 23a + 150 | 0.7500  (0.4059–1.0) | 50.00 | 100 | 75.00 | 66.67 |
|  | 23a + CEA | 0.9444  (0.7709–1.0) | 83.33 | 100 | 91.67 | 90.91 |
|  | 92a + 125a | 0.6667  (0.3056–1.0) | 66.67 | 83.33 | 75.00 | 72.73 |
|  | 92a + 150 | 0.7222  (0.3750–1.0) | 66.67 | 83.33 | 75.00 | 72.73 |
|  | 92a + CEA | 0.9444  (0.7714–1.0) | 100 | 83.33 | 91.67 | 92.31 |
|  | 125a + 150 | 0.8889  (0.6296–1.0) | 83.33 | 83.33 | 83.33 | 83.33 |
|  | 125a + CEA | 0.8333  (0.5000–1.0) | 83.33 | 100 | 91.67 | 90.91 |
|  | 150 + CEA^b)^ | 1.0  (1.0–1.0) | 100 | 100 | 100 | 100 |
| **3-marker combinations** | 23a + 92a + 125a | 0.5000  (0.1111–0.8617) | 33.33 | 100 | 66.67 | 50.00 |
|  | 23a + 92a + 150 | 0.7222  (0.3333–1.0) | 66.67 | 100 | 83.33 | 80.00 |
|  | 23a + 92a + CEA | 0.8333  (0.5143–1.0) | 50.00 | 100 | 75.00 | 66.67 |
|  | 23a + 125a + 150 | 0.5833  (0.1667–1.0) | 50.00 | 100 | 75.00 | 66.67 |
|  | 23a + 125a + CEA | 0.9444  (0.7778–1.0) | 100 | 83.33 | 91.67 | 92.31 |
|  | 23a + 150 + CEA | 0.6944  (0.3142–1.0) | 50.00 | 100 | 75.00 | 66.67 |
|  | 92a + 125a + 150 | 0.6944  (0.2857–1.0) | 50.00 | 100 | 75.00 | 66.67 |
|  | 92a + 125a + CEA | 0.8611  (0.6000–1.0) | 83.33 | 83.33 | 83.33 | 83.33 |
|  | 92a + 150 + CEA | 0.9444  (0.7500–1.0) | 83.33 | 100 | 91.67 | 90.91 |
|  | 125a + 150 + CEA | 0.8611  (0.5714–1.0) | 83.33 | 83.33 | 83.33 | 83.33 |
| **4-marker combinations** | 23a + 92a + 125a + 150 | 0.7500  (0.3750–1.0) | 66.67 | 100 | 83.33 | 80.00 |
|  | 23a + 92a + 125a + CEA | 0.7500  (0.3714–1.0) | 66.67 | 100 | 83.33 | 80.00 |
|  | 23a + 92a + 150 + CEA | 0.9167  (0.6667–1.0) | 100 | 83.33 | 91.67 | 92.31 |
|  | 23a + 125a + 150 + CEA | 0.8333  (0.5000–1.0) | 66.67 | 100 | 83.33 | 80.00 |
|  | 92a + 125a + 150 + CEA | 0.7778  (0.4286–1.0) | 66.67 | 100 | 83.33 | 80.00 |
| **5-marker combinations** | 23a + 92a + 125a + 150 + CEA | 0.8333  (0.4996–1.0) | 83.33 | 83.33 | 83.33 | 83.33 |

^a)^The EV-derived miRNAs are labeled simply as 23a, 92a, 125a, and 150.

^b)^One optimal combinations.

^c)^95% CI, 95% confidence interval.

**Table S8.** Diagnostic performance of 31 blood biomarker combinations by ZAHV-AI system for CRC stage 4.

| **Biomarker combinations**^a)^  (Total of 31 combinations) | | **Test set (*n* = 12)** | | | | |
| --- | --- | --- | --- | --- | --- | --- |
|  |  | **AUC**  (95% CI)^c)^ | **Sensitivity**  (%) | **Specificity**  (%) | **Accuracy**  (%) | **F1 Score**  (%) |
| **Single**  **markers** | 23a | 0.5556  (0.1712–0.9067) | 100 | 33.33 | 66.67 | 75.00 |
|  | 92a | 0.8056  (0.4439–1.0) | 50.00 | 100 | 75.00 | 66.67 |
|  | 125a | 0.7778  (0.4000–1.0) | 66.67 | 100 | 83.33 | 80.00 |
|  | 150^b)^ | 1.0  (1.0–1.0) | 100 | 100 | 100 | 100 |
|  | CEA | 0.9722  (0.8519–1.0) | 83.33 | 100 | 91.67 | 90.91 |
| **2-marker combinations** | 23a + 92a | 0.7778  (0.4443–1.0) | 66.67 | 83.33 | 75.00 | 72.73 |
|  | 23a + 125a | 0.8611  (0.5623–1.0) | 83.33 | 100 | 91.67 | 90.91 |
|  | 23a + 150^b)^ | 1.0  (1.0–1.0) | 100 | 100 | 100 | 100 |
|  | 23a + CEA | 0.9444  (0.7500–1.0) | 83.33 | 100 | 91.67 | 90.91 |
|  | 92a + 125a | 0.8889  (0.6362–1.0) | 83.33 | 83.33 | 83.33 | 83.33 |
|  | 92a + 150 | 0.9167  (0.6875–1.0) | 83.33 | 100 | 91.67 | 90.91 |
|  | 92a + CEA | 0.9444  (0.7500–1.0) | 83.33 | 100 | 91.67 | 90.91 |
|  | 125a + 150 | 0.9722  (0.8286–1.0) | 83.33 | 100 | 91.67 | 90.91 |
|  | 125a + CEA^b)^ | 1.0  (1.0–1.0) | 100 | 100 | 100 | 100 |
|  | 150 + CEA^b)^ | 1.0  (1.0–1.0) | 100 | 100 | 100 | 100 |
| **3-marker combinations** | 23a + 92a + 125a | 0.9444  (0.7429–1.0) | 100 | 83.33 | 91.67 | 92.31 |
|  | 23a + 92a + 150 | 0.9167  (0.6870–1.0) | 83.33 | 100 | 91.67 | 90.91 |
|  | 23a + 92a + CEA | 0.7778  (0.3882–1.0) | 83.33 | 83.33 | 83.33 | 83.33 |
|  | 23a + 125a + 150^b)^ | 1.0  (1.0–1.0) | 100 | 100 | 100 | 100 |
|  | 23a + 125a + CEA | 0.9722  (0.8750–1.0) | 83.33 | 100 | 91.67 | 90.91 |
|  | 23a + 150 + CEA | 0.9444  (0.7500–1.0) | 100 | 83.33 | 91.67 | 92.31 |
|  | 92a + 125a + 150 | 0.9444  (0.7429–1.0) | 100 | 83.33 | 91.67 | 92.31 |
|  | 92a + 125a + CEA | 0.9722  (0.8519–1.0) | 83.33 | 100 | 91.67 | 90.91 |
|  | 92a + 150 + CEA^b)^ | 1.0  (1.0–1.0) | 100 | 100 | 100 | 100 |
|  | 125a + 150 + CEA^b)^ | 1.0  (1.0–1.0) | 100 | 100 | 100 | 100 |
| **4-marker combinations** | 23a + 92a + 125a + 150^b)^ | 1.0  (1.0–1.0) | 100 | 100 | 100 | 100 |
|  | 23a + 92a + 125a + CEA | 0.9722  (0.8518–1.0) | 83.33 | 100 | 91.67 | 90.91 |
|  | 23a + 92a + 150 + CEA | 0.9722  (0.8518–1.0) | 83.33 | 100 | 91.67 | 90.91 |
|  | 23a + 125a + 150 + CEA^b)^ | 1.0  (1.0–1.0) | 100 | 100 | 100 | 100 |
|  | 92a + 125a + 150 + CEA^b)^ | 1.0  (1.0–1.0) | 100 | 100 | 100 | 100 |
| **5-marker combinations** | 23a + 92a + 125a + 150 + CEA^b)^ | 1.0  (1.0–1.0) | 100 | 100 | 100 | 100 |

^a)^The EV-derived miRNAs are labeled simply as 23a, 92a, 125a, and 150.

^b)^Eleven optimal combinations.

^c)^95% CI, 95% confidence interval.
